# Systematic review of cnidarian microbiomes reveals insights into the structure, specificity, and fidelity of marine associations

**DOI:** 10.1038/s41467-023-39876-6

**Published:** 2023-08-14

**Authors:** M. McCauley, T. L. Goulet, C. R. Jackson, S. Loesgen

**Affiliations:** 1 Department of Chemistry, Whitney Laboratory for Marine Bioscience, University of Florida, St. Augustine, FL USA; 2 Department of Biology, University of Mississippi, University, MS USA; 3 Present Address: U.S. Geological Survey, Wetland and Aquatic Research Centre, Gainesville, FL USA

**Keywords:** Data processing, Marine biology, Microbiome

## Abstract

Microorganisms play essential roles in the health and resilience of cnidarians. Understanding the factors influencing cnidarian microbiomes requires cross study comparisons, yet the plethora of protocols used hampers dataset integration. We unify 16S rRNA gene sequences from cnidarian microbiome studies under a single analysis pipeline. We reprocess 12,010 cnidarian microbiome samples from 186 studies, alongside 3,388 poriferan, 370 seawater samples, and 245 cultured Symbiodiniaceae, unifying ~6.5 billion sequence reads. Samples are partitioned by hypervariable region and sequencing platform to reduce sequencing variability. This systematic review uncovers an incredible diversity of 86 archaeal and bacterial phyla associated with Cnidaria, and highlights key bacteria hosted across host sub-phylum, depth, and microhabitat. Shallow (< 30 m) water Alcyonacea and Actinaria are characterized by highly shared and relatively abundant microbial communities, unlike Scleractinia and most deeper cnidarians. Utilizing the V4 region, we find that cnidarian microbial composition, richness, diversity, and structure are primarily influenced by host phylogeny, sampling depth, and ocean body, followed by microhabitat and sampling date. We identify host and geographical generalist and specific *Endozoicomonas* clades within Cnidaria and Porifera. This systematic review forms a framework for understanding factors governing cnidarian microbiomes and creates a baseline for assessing stress associated dysbiosis.

## Abstract (web summary)

This study unified cnidarian microbiome data from 186 studies (~ 6.5 billion sequence reads), providing novel insights into cnidarian microbial communities and highlighting key bacteria across sub-phylum, geography, depth and microhabitat. Understanding factors governing microbiome health will support ongoing and future coral preservation efforts.

## Introduction

Cnidarians (e.g., corals, sea anemones, jellyfish) face anthropogenic stressors that are increasing in severity and frequency. Globally, the ecosystem services that corals and other cnidarians provide have fallen by approximately 50%^1^, with further decreases forecasted in the future^2^. Since many organisms reside within and around cnidarians, loss of cnidarian species has resulted in a 63% decline in coral reef associated biodiversity, including macroinvertebrates and fish^1^.

In addition to macroorganisms, cnidarians host a multitude of microorganisms, which together constitute their microbiome^3–6^. Research on the mutualistic symbiosis between cnidarians and dinoflagellate algae (family Symbiodiniaceae), including the bleaching phenomenon (loss of Symbiodiniaceae and/or their chlorophyll content), overshadows the knowledge on the Archaea, Bacteria, Fungi, microalgae, protists, and viruses of the cnidarian microbiome^7–11^. Nevertheless, the non-Symbiodiniaceae members of cnidarian microbiomes play important roles within the holobiont^12^ (host and microbiota). To understand cnidarians under current climate conditions, and cnidarian capacity to withstand environmental change, it is imperative to understand the fundamental factors influencing the microbiome composition of cnidarians.

The cnidarian microbiome functions in host nutrient cycling (C, N, P, S) (reviewed in^13–16^), homeostasis (reviewed in^17,18^), protection, including antimicrobial production and competitive exclusion (reviewed in^19–21^), development (reviewed within^22^), health (reviewed in^23,24^), and response to environmental fluctuations (reviewed in^25,26^), in addition to contributing to reef processes (reviewed in^27,28^), and ecosystem resilience (reviewed in^16,29^). These findings were made possible with the increasing access to DNA sequencing and prompted by the increased concern for coral reef health^30–32^.

The majority of cnidarian microbiome research has utilized 16S rRNA amplicon sequencing of scleractinian coral microbiomes to identify primarily bacterial assemblages. As the main frame builders of coral reefs, scleractinian corals (class Hexacorallia, sub-phylum Anthozoa^33^), have been the main emphasis of cnidarian microbiome research. This focus has framed our current understanding of cnidarian microbiomes. Research into non-scleractinian cnidarians, however, has identified significantly different structuring of cnidarian microbial symbioses^34–36^. Unfortunately, the lack of standardized sampling, storage, processing, and analytical protocols across 16S rRNA gene amplicon studies, hinders the ability to synthesize published data to create a baseline for cnidarian microbiomes^37–39^. Thus, the scientific community often relies on literature reviews in lieu of meta-analyses or systematic reviews^37,40,41^. The methodological variability not only challenges data integration but can lead to different conclusions due to identifying significantly different microbial communities from the same coral sample^42^.

In addition to variability resulting from different processing protocols, the majority of cnidarian datasets published to date have been processed by clustering (typically to 97% similarity) their sequences into operational taxonomic units (OTUs), with the remaining datasets denoised to produce amplicon sequence variants (ASVs). Clustering within datasets limits detailed examination of trends across available literature, as OTUs from one study are inherently different from those from another. Further, while both OTU and ASV approaches have their strengths and weaknesses^43,44^, the denoising steps in ASV pipelines can increase the precision of measuring environmental specificity^45^ and, importantly, allow for direct comparison of sequences across studies. However, the production of ASVs across different denoising pipelines, or different parameters, can also introduce sequence variation.

**Fig. 1:**
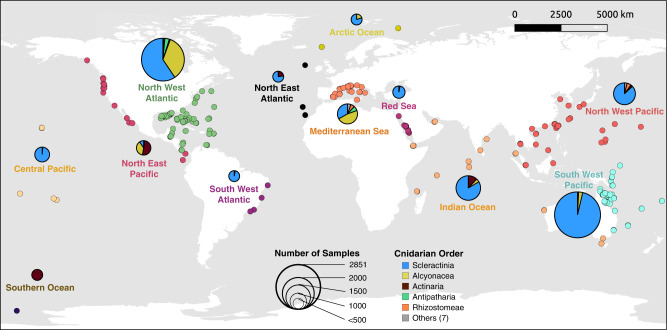
Global distribution of the 12,010 cnidarian microbial (16S rRNA gene) samples included in the systematic review. Regional pie chart diameters indicate sample abundance, with colors representing the relative abundance of five cnidarian orders and an ‘other’ category which includes seven additional cnidarian orders. Smaller circles represent studies with at least one cnidarian microbiome sample, with colors specific to water bodies. Made with QGIS.

In this study we unify available cnidarian 16S rRNA sequences (Fig. 1, Supplementary Data 1) under a single analysis pipeline, thereby creating a global baseline for cnidarian microbiomes, and elucidating patterns and factors that govern the assemblage of cnidarian microbiomes. Importantly, this database is freely available (Figshare) to serve as a community resource for further exploration into cnidarian microbiomes, and to support global conservation strategies. We assemble all available 16S rRNA sequences from nearly 200 studies, each of which preserved, extracted, amplified, processed, and analyzed samples using distinct techniques. Partitioning these samples according to host health, sampling effort, hypervariable region, and sequencing platforms, we reduce the methodological and sequencing variability present for cross-study analyses. We compare the diversity, structure, and richness of scleractinian coral prokaryotic microbial communities to other members of the phylum Cnidaria, to determine how phylogeny, geography, and depth influence cnidarian microbiomes. We investigate the relative abundance of microbiota as a function of depth, and identify the taxonomic ‘core microbiome’ (microbiota present in all samples within a species per site), as this microbial component often serves as a proxy for holobiont health and resilience when facing anthropogenic stressors^20,46^. Further, we examine differences in the microbial relative abundance in three different coral microhabitats (tissue, skeleton, and mucus) by using unique sequence and clustering-based techniques. Finally, we document microbial genera (including *Endozoicomonas*) that are highly prevalent and abundant within the different microhabitats of cnidarian species, serving as core members across cnidarian classes.

## Results

### Available 16S rRNA sequences

A total of 12,010 cnidarian 16S rRNA sequences were processed, alongside 3388 poriferan, 370 seawater, and 245 cultured Symbiodiniaceae, to produce a final database of 16,012 samples. This database was partitioned according to sample metadata, including host health status, sampling effort per site, amplified hypervariable region, and sequencing chemistry (Table 1). Primarily, this included six ASV libraries that span the amplified hypervariable regions: V1-V2, V3, V4, V5-V6, V7-V8, alongside overlapping V4 cnidarian and poriferan samples (V4_p; Table 1). The cnidarian V4 library was further partitioned by sequencing chemistry and cnidarian classes, to reduce the effects of the MiSeq processing and scleractinian sampling bias. Additionally, the relative abundances of microbial communities were investigated within cnidarian tissue, skeletal, and mucus samples across all hypervariable regions, whereby a minimum of four ʻhealthyʼ (no record of observed stress) cnidarian individuals were sampled per site (Table 1).

**Table 1:** The number of studies, species, samples, and amplicon sequence variants (ASVs) included in the various datasets analyzed in the systematic review

	Studies	Species	Samples	ASVs
Entire Database	186	495	16,012	
-Cnidaria	177	212	12,010	
--V1-V2 Library	19	46	1949	7934
--V3 Library	43	102	2106	212,316
--V4 Library	85	233	5979	360,006
--V4_p Library	86	467	9737	493,157
--V5-V6 Library	44	52	3751	128,686
--V7-V8 Library	9	13	469	11,893
---V4_MiSeq_'healthy'_cnidarian_unrarified	63	196	4243	167,227
---V4_MiSeq_'healthy'_cnidarian_rarified	49	150	2964	131,761
---V4_MiSeq_'healthy'_scleractinian_unrarified	53	149	3546	163,004
---V4_MiSeq_'healthy'_scleractinian_rarified	34	103	2275	127,384
---V4_MiSeq_'healthy'_nonscleractinian_unrarified	24	47	697	31,550
---V4_MiSeq_'healthy'_nonscleractinian_rarified	18	37	495	24,400
----Relative Abundance Analyses	143	131	7067	588,776

### Taxonomic variability in cnidarian 16S rRNA sequences

**Fig. 2:**
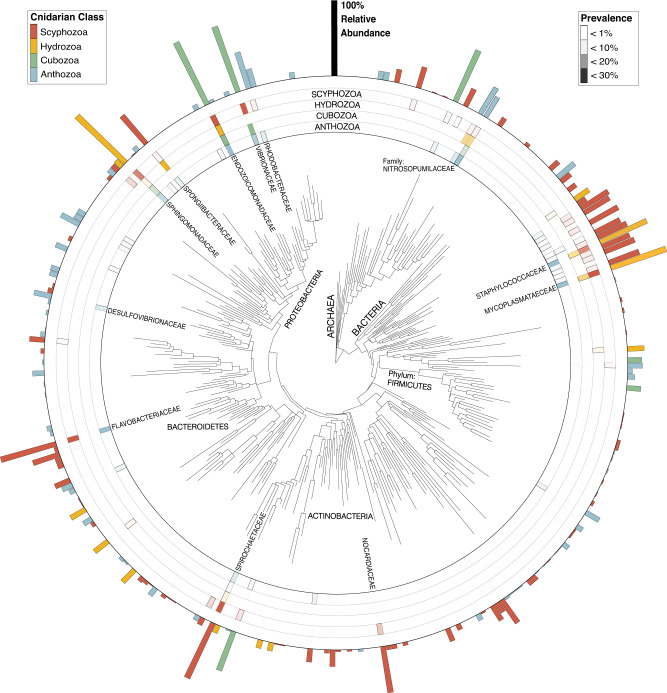
Phylogeny, prevalence, and relative abundance of the most prevalent archaeal and bacterial families identified in the V4 library. The heat maps represent the prevalence (% of total samples detected in) of different microbial families across the cnidarian classes, while the outer bar charts display the cnidarian class with the greatest average relative abundance. The most prevalent and relatively abundant microbial families are annotated.

Across the phylum Cnidaria, 12 archaeal and 74 bacterial phyla (22 and 185 classes, respectively) were identified from 720,835 ASVs present in libraries of five regions of 16S rRNA cnidarian genes (V1-V2, V3, V4, V5-V6, V7-V8). Out of 697,284 ASVs classified as bacterial, the family Endozoicomonadaceae (Gammaproteobacteria) exhibited the greatest prevalence in each of the four cnidarian classes: Anthozoa, Cubozoa, Hydrozoa, and Scyphozoa. Vibrionaceae (Gammaproteobacteria) was prevalent in three of the four cnidarian classes, with the exception of Hydrozoa (visualized with the V4 library, Fig. 2). Both of these bacterial families also presented the highest average relative abundance in the microbial samples that they were present in. Strikingly, the vast majority of cnidarian associated microbiota were rare (<1% prevalence) and contributed little to the relative abundance of their host microbiome (visualized with the V4 library, Fig. 2).

**Fig. 3:**
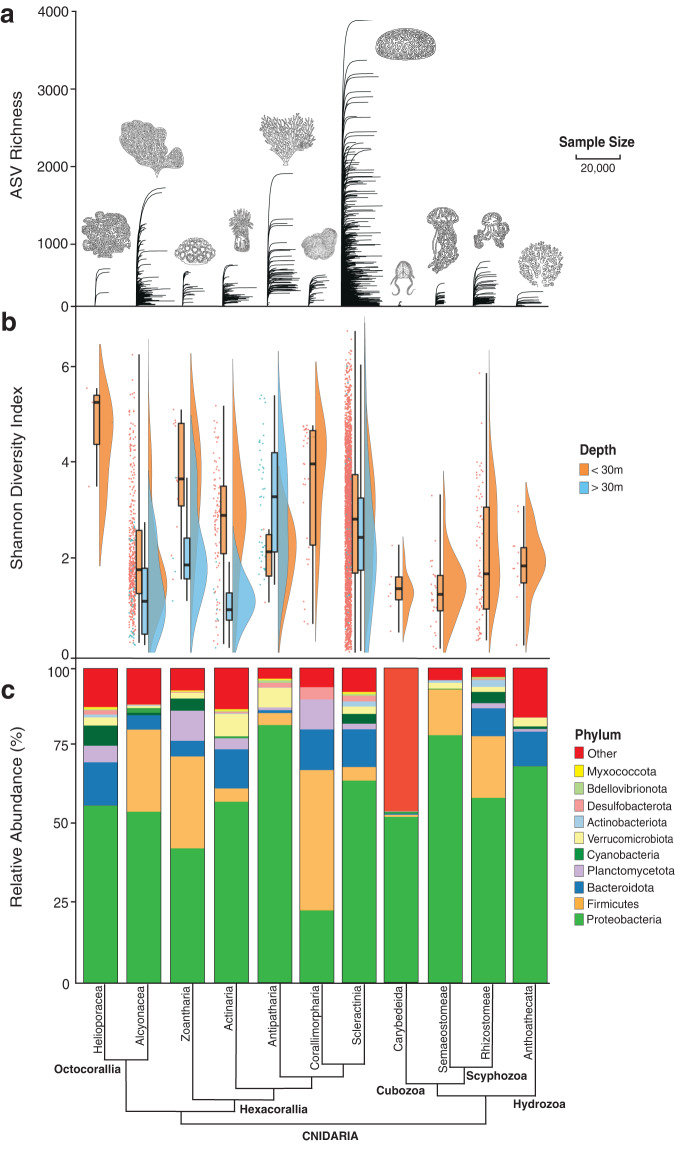
Microbial diversity and abundance in the phylum Cnidaria. **a** Rarefaction plot of amplicon sequence variants (ASV) richness (ASVs observed) illustrating the high sequencing coverage across the 11 cnidarian orders within the V4 library. Sequence depth was trimmed to 20,000 for visual purposes only. **b** Sample Shannon index values (dots), averages (bar plot), and distributions (half violin) are colored by depth per cnidarian order (*n* = 3840 samples). Box plot whiskers represent the minimum and maximum values, with bounds of the box representing the 25th and 75th percentile, and the median in the center. **c** The relative abundance of the 10 most abundant bacterial phyla per cnidarian order. Cnidarian orders are arranged phylogenetically^119,120^.

Proteobacteria (currently ascribed as Pseudomonadota) was the dominant bacterial phyla (40.3%), followed by Firmicutes (currently ascribed as Bacillota) constituting 16.1%, and Bacteroidetes (currently ascribed as Bacteroidota) at 8.3% (Fig. 3c). Archaea were dominated by Nanoarchaeota (59.6%), followed by Crenarchaeota (22.6%).

### The diversity of cnidarian microbiomes (α-diversity)

For the subset of ‘healthy’ Illumina MiSeq samples (V4_MiSeq_healthy_cnidarian library), microbial diversity was significantly correlated with cnidarian phylogeny, life stage, hosting Symbiodiniaceae, as well as collection depth, and location (observed ASVs, Shannon Diversity and Inverse Simpson index; Supplementary Tables 1–2). Consistent across unrarefied and rarefied data sets, sampling water body was often the strongest influence, explaining up to 43% of the microbial diversity within scleractinian corals, and up to 33% in non-scleractinians (Supplementary Tables 1–2).

With and without rarefaction, class Hexacorallia contained a significantly greater microbial diversity than Discomedusae, Hydroidolina, and Octocorallia (*p adj.*<0.02, Supplementary Fig. 1). Cnidarian orders were separated into three groups based on the diversity indices of microbial ASVs identified within the Illumina MiSeq V4 library. The first, representing only Scleractinia, yielded the greatest average (894) and maximum (3794) number of ASVs per sample. Scleractinia also hosted the greatest range of microbial richness, Shannon and Inverse Simpson index values, although this is partially a result of sampling bias (Fig. 3a, b, Supplementary Figs. 2–3). The second group contained both Alcyonacea and Antipatharia, hosting an average of 427 ASVs per sample and a maximum of 2000 ASVs, with index values generally higher than the remaining seven cnidarian orders (Actiniaria, Anthoathecata, Corallimorpharia, Helioporacea, Rhizostomeae, Semaeostomeae, and Zoantharia), although these differences were not as consistently significant as observed with Scleractinia (Fig. 3a, Supplementary Fig. 2). The third group consisted of the seven least diverse remaining cnidarian orders, with an average of 103 ASVs per sample.

Increased microbial diversity was significantly correlated with shallower sampling sites in alcyonaceans, scleractinians, and zoanthids, (98.3%, 82.7%, 100% depth metadata available per samples, respectively) whereas the opposite trend was present in Antipatharia (99.6% depth metadata available, *p adj.*<0.02, Fig. 3b, Supplementary Figs. 4–7). Scleractinians from the ‘complex’ lineage (often creating more porous skeletal structures)^47^ hosted a significantly (*p adj*.<0.02) more diverse microbiome in comparison to species of the ‘robust’ lineage (often creating more solid skeletal structures) (Supplementary Fig. 8). A trend toward a greater microbial diversity in scleractinian mucus and skeleton compared to tissue was also identified, suggesting a highly species-specific relationship (*p* = 0.09). The microhabitat trend, however, did not occur in non-scleractinian cnidarians, potentially indicating a more niche dependent microbiome in scleractinian species (Supplementary Tables 1–2).

### The structure of cnidarian microbiomes (β-diversity)

Dispersion of microbial dissimilarities within the V4_MiSeq_ʻhealthyʼ_cnidarian library was significantly different between scleractinian and non-scleractinian cnidarians, which may be partially due to the oversampling of the former (*p* < 1 × 10^43^, Supplementary Tables 3–6). Subsequently, scleractinian corals were separated from non-scleractinian cnidarians, and all diversity metrics were repeated on the separated data sets (Supplementary Tables 1–2). All tested parameters including, host phylogeny, life-stage, collection depth, hosting Symbiodiniaceae, original water body, and DNA extraction method were significantly correlated to microbial structure (Bray-Curtis, and Jaccard dissimilarity indices) of scleractinian and non-scleractinian cnidarians (*p* < 0.004, Supplementary Tables 1–2). The strongest influencers of both the unrarefied and rarefied Illumina MiSeq subset, were phylogenetic history, followed by original water body, although the strengths of their correlations varied between scleractinian and non-scleractinian data subsets (Supplementary Figs. 9–10, Supplementary Tables 1–2).

By hierarchically clustering the Bray-Curtis dissimilarity index values of the V4_MiSeq_healthy_cnidarian library, species within Octocorallia, Scyphozoa, and Hydrozoa formed numerous clusters that were often geographically distinct, similar to tight clusters of species within Actinaria, Antipatharia, Corallimorpharia, and Zoantharia. Strong clustering of scleractinian microbiome samples within family and water bodies occurred alongside geographically closer sampling sites also clustered closer together (Supplementary Fig. 11). These geographical signals were observed again within the V4_p library (combining cnidarian and poriferan samples), with scleractinian microbiome samples spanning almost the entire dissimilarity distance, with numerous small groupings of robust corals, within the wider array of complex species (Supplementary Fig. 12). The majority of high and low microbial abundance sponges represented the extremes of the dissimilarity dendrogram (Supplementary Fig. 12). While some scleractinians and actinarians hosted microbiomes similar to those of low microbial abundance sponges, the majority of cnidarian microbiomes fell in-between these sponge types or were more similar to high microbial abundance sponges (Supplementary Fig. 12).

### The specificity of cnidarian microbiomes

**Fig. 4:**
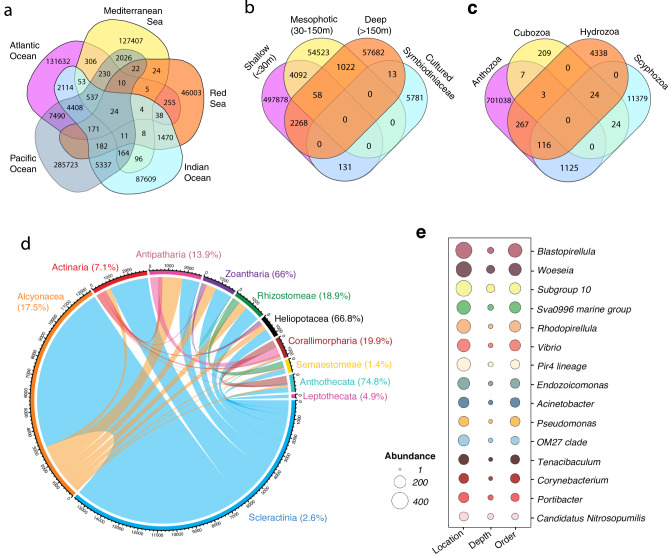
Distribution of shared microbial sequences within the phylum Cnidaria. Absolute abundance of archaeal and bacterial amplicon sequence variants (ASVs) identified in, and shared across, **a** major water bodies, **b** sampling depth, **c** cnidarian classes. **d** ASVs identified in at least one cnidarian order, with arc width representing the absolute abundance of shared ASVs. The relative abundance of shared sequences (within all sequences identified in that order) is presented as a %. **e** The absolute abundances of the 15 most shared bacterial genera, with ASVs identified across major water bodies (location), depth, and cnidarian order, representing the most globally distributed cnidarian-associated bacteria.

Of the 697,284 bacterial ASVs present in the five cnidarian hypervariable libraries (Table 1), fewer than 10% were shared across cnidarian orders, with the majority (71.1%) identified within Scleractinia, followed by 7.6% identified in Alcyonacea and 5.3% to Actinaria, regardless of geographic location (Table 1; Supplementary Fig. 13). Nevertheless, cnidarian microbiomes were structured strongly across water bodies, with 93.6% of all ASVs identified in a single water body, and less than 3.6% shared across more than two water bodies (Fig. 4a). There was also intense structuring with depth, with just 1% of ASVs shared across depths (Fig. 4b). Of the ASVs identified in shallow waters (<30 m), 98.7% were found only at that depth range, followed by 90.1% of those identified in mesophotic depths (30–150 m) unique to that range, and 94.4% of those identified in deeper waters (>150 m) (Fig. 4b). Further, only 2.6% of ASVs identified in cultured Symbiodiniaceae microbiomes were identified in any cnidarian microbiome, and primarily from those sampled in shallow waters (V3, V4 libraries; Fig. 4b). While Scyphozoa shared the greatest number of sequences with Anthozoa when compared to Cubozoa and Hydrozoa, they constituted less than 0.001% of all ASVs (Fig. 4c).

Over two thirds (67.3%) of the sequences shared across cnidarian families were sequenced from Scleractinia and Alcyonacea (Fig. 4d). While Scleractinia hosted 35.6% of all shared sequences, they accounted for only 2.6% of microbes exclusive to Scleractinia, as opposed to the 31.7% of shared sequences found in Alcyonacea accounting for 17.5% of their entire microbiome. The absolute abundance of shared sequences was not solely a result of biased sampling effort, as a similar number of Actinaria and Alcyonacea sequences were included in this study, with Actinaria hosting fewer than 10% of shared sequences. The relative abundance of shared sequences varied widely across cnidarian orders from Semaestomeae sharing the least (1.4% of their entire microbiome), to the Anthoathecata sharing the most (74.8% of their entire microbiome), even with a similar number of samples in the library. Alcyonaceans shared more sequences with cnidarians outside of their order (17.5%) than within (10.2%), whereas the Scleractinia shared more sequences within their order (12.3%) than outside of it (2.6%).

Across all scleractinian microbiomes, 86.1% of sequences were exclusive to cnidarian tissue samples, 6.7% to mucus, and 5.4% to skeleton, with 1.8% found in other tissue types (Supplementary Data 1). Only 2.4% of the scleractinian microbiome was shared between tissue and mucus samples, which was greater than the 1.2% shared between the skeleton and tissue. Relatively few ASVs were identified in both skeleton and mucus samples (0.2%), while even fewer were identified in tissue, mucus, and skeleton samples (0.1%). ASVs were more likely to be shared between the tissue and mucus from non-Hexacorallia and Hexacorallia samples (0.2% and 0.1%, respectively), than between skeletons (<0.01%). Surprisingly, the identities of shared bacterial genera across cnidarian orders were also those most likely to be shared across depth and geography (Fig. 4e). Because the data are inherently dependent on the techniques used to partition samples from the different microhabitats, many previous studies have inadvertently mixed these location specific microbes during processing. Given the distinct communities present within microhabitats within individual cnidarians, future studies investigating the functional role of these microbiota should utilize methods that minimize overlap in microorganisms derived from the mucus, tissue, and skeleton microhabitats of the coral holobiont^48^.

### Core microbiota of ʻhealthyʼ cnidarians

**Fig. 5:**
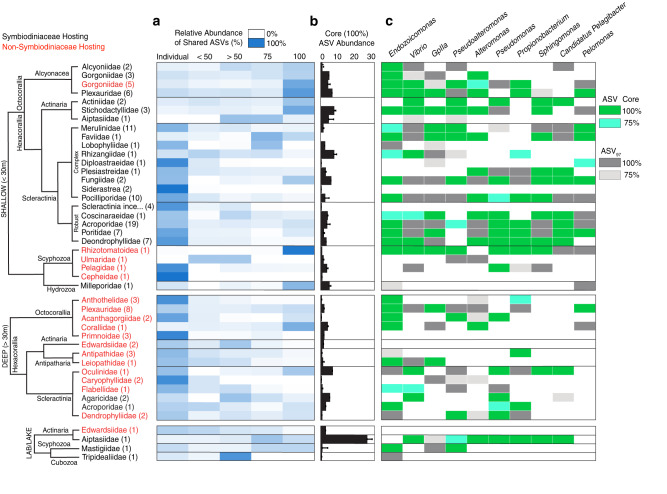
Abundance and composition of the cnidarian core microbiome across depth. **a** The relative abundance of archaeal and bacterial sequences identified within tissue samples per site, averaged to cnidarian family. The number of species present within each family is included in parenthesis. Columns represent the relative abundance of Amplicon Sequence Variants (ASVs) present within a single (Individual), fewer than 50% (<50), more than 50% (>50), at least 75% (75), or present in all (100) cnidarians sampled per site. **b** The average absolute abundance of ASVs identified in 100% of individuals sampled per site, with standard error. **c** The identity of the 10 most commonly present bacteria in 75 and 100% of individuals sampled per site, as an ASV or an ASV_97_ (clustered at 97% similarity). Colored boxes indicate if bacteria were identified as a core member in at least one site per species. Cnidarian species were only included if there was a minimum of four ʻhealthyʼ individuals per site.

To examine the relative distribution of microbes within Cnidaria, an additional dataset was created from all studies in the original Cnidaria database, specifically including all ‘healthy’ cnidarians, for which at least four individuals were collected per sampling site (Table 1). Cnidarian order and sampling depth were significantly correlated with the proportion of bacteria present in all cnidarian tissue samples at a site (*p* < 0.001, *p* < 0.05, respectively) and those identified only in a single cnidarian sample per site (*p* < 0.005, *p* < 0.05, respectively), with deeper cnidarians hosting a more individualized microbiome, whereas shallow water Cnidaria (primarily Alcyonacea and Actinaria) generally hosting a more abundant core microbiome (Fig. 5). Hosting Symbiodiniaceae significantly influenced the proportion of the microbiota identified in all cnidarians sampled per site (*p* < 0.05), but not that identified in a single cnidarian (*p* > 0.05).

Comparing the average relative abundances of both the clustered and unique sequence analyses revealed cnidarian and microhabitat variability (Supplementary Figs. 14–16). Use of clustered sequences instead of unique sequences, when examining colony-specific microbiota in scleractinian tissue and skeletal samples, resulted in a 61.2% and 63.8% increase in their average relative abundances. A similar comparison with scleractinian mucus samples resulted in only a 45.4% increase (Supplementary Figs. 14–15). Further, using clustered instead of unique sequences yielded a 55% increase in the relative abundance of what constituted a ‘core microbiota’ in all samples for scleractinian Acroporidae tissues, while clustering resulted in a 21% reduction of the ‘core microbiota’ in the alcyonacean Plexauridae (Supplementary Fig. 16). The use of clustered sequences may therefore be problematic when comparing microbial communities of different cnidarians or different microhabitats.

Within their tissue microhabitat, scleractinian coral colonies hosted a significantly greater proportion of microbes (ASVs) identified in a single colony compared to shallow water (<30 m) actinarians, alcyonaceans, and hydrozoans, (*p* < 0.05, for all, Fig. 5a). However, there was a significant shift to a greater proportion of microbiota identified only in a single cnidarian sample with actinarian and alcyonaceans sampled deeper than 30 m (*p* < 0.05, Fig. 5a). All ambient collected cnidarian samples hosted an average of <10 core (100%) ASVs, although this varied significantly within species sampled at different sites (*p* < 0.05, Fig. 5b). The major exception to this were cultured sea anemones, *Exaiptasia pallida*, which hosted on average six times the number of core ASVs of field-collected *E. pallida*.

Core microbiota within mucus samples presented a similar pattern, with the majority of scleractinians hosting a highly colony-specific community, while concurrently hosting a group of commonly shared microbial genera (Supplementary Fig. 14). Of all microhabitats however, skeletal microbiomes demonstrated not only the greatest relative abundance of ASVs present within a single scleractinian colony (68.7%, compared to 48.6% in mucus, and 53.0% in tissues), they also shared few core microbiota across species (Supplementary Fig. 15).

The most commonly occurring microbiota within cnidarian tissues, including GpIIa, *Vibrio, Pseudoalteromonas*, *Alteromonas, Pseudomonas*, and *Propionibacterium* were generally present in the core microbiome of most cnidarian orders across depth, while *Sphingomonas, Pelomonas*, and *Candidatus Pelagibacter* were more commonly abundant in shallow waters (<30 m) (Fig. 5c). Of the 143 studies that examined the relative abundance of microbiota, only 19 included either PCR, and/or extraction kit controls. Nevertheless, after we excluded bacterial sequences identified as potential contaminants we still detected core communities containing *Alteromonas*, *Pseudoalteromonas*, and *Propionibacterium*, suggesting that these taxa are not kit contaminants^49,50^

*Endozoicomonas* was the most commonly identified core bacterial genus across all cnidarians, including shallow water non-Symbiodiniaceae hosting cnidarian species, and cnidarians living deeper than 30 m (Fig. 5c). Additionally, while GpIIa, *Candidatus Pelagibacter, Vibrio*, and *Pseudoalteromonas* were identified as common core microbiota across both tissue and mucus samples, *Endozoicomonas* was the only bacterial genus that commonly occurred across tissue, mucus, and skeletal microhabitats (Fig. 5c, Supplementary Figs. 14–16).

### *Endozoicomonas* case study

**Fig. 6:**
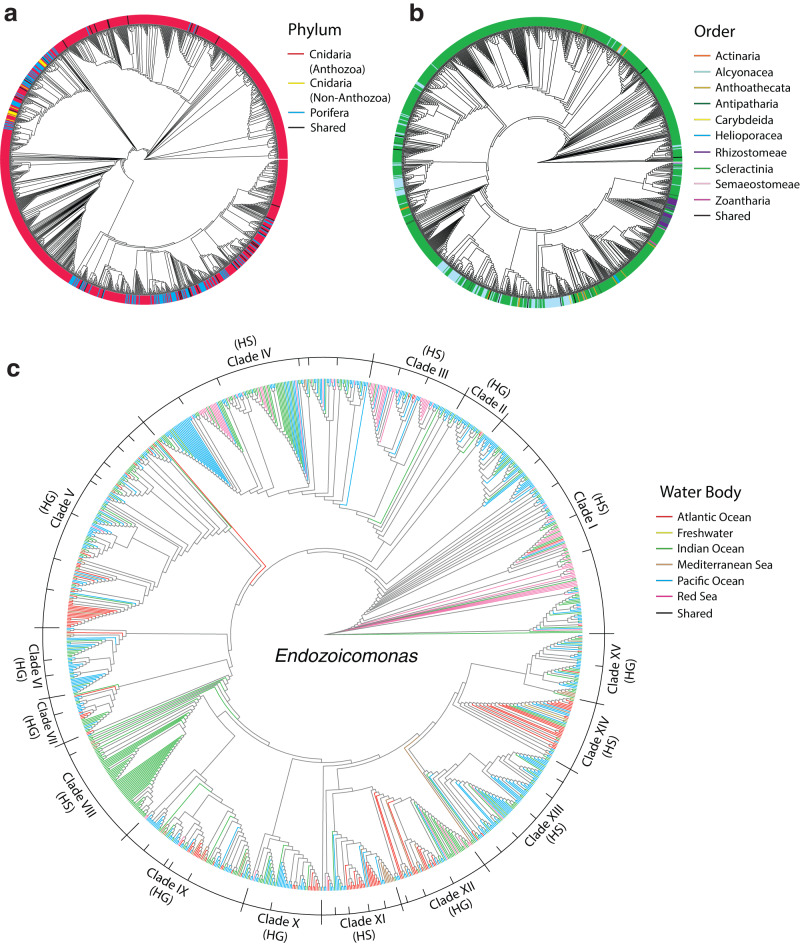
Phylogenies of *Endozoicomonas* sequences isolated from the phyla Cnidaria and Porifera. **a** *Endozoicomonas* phylogeny of amplicon sequence variants (ASVs) identified within Cnidaria and Porifera (V4_p library). **b** ASVs identified within Cnidaria (V4 library) with tips colored by cnidarian order. **c** branches colored by water body. Major clades were identified according to branch length and ASV clustering and labelled as Host Specific (HS) or Host Generalist (HG). Tree branch lengths are normalized for visual purposes only.

To further examine the bacterial genus that was the most prevalent across cnidaria, globally ubiquitous, and also the most commonly identified in the core microbiome, we examined *Endozoicomonas* ASVs within all cnidarian libraries (V1-V2, V3, V4, V5-V6, and V7-V8), as well as within both Cnidaria and Porifera (V4_p; Table 1). Sequence variation in these ASVs suggested 15 possible major clades of this genus, that revealed host and geographical distribution (Fig. 6, Supplementary Fig. 17).

Major clades were generally identified across all libraries, with a few minor clades expanding and collapsing depending on sampling effort. Neither major nor minor clades were singularly attributable to sequencing platform, abundance available reads or extraction techniques. 98.6% of *Endozoicomonas* ASVs identified in Porifera and non-anthozoan cnidarians were present in only a few closely related major clades, whereas all major clades included *Endozoicomonas* ASVs hosted within Anthozoans (Fig. 6a). Within Cnidaria, *Endozoicomonas* ASVs were identified in eight orders (Actinaria, Alcyonacea, Antipatharia, Corallimorpharia, Helioporacea, Rhizostomeae, Scleractinia, Zoantharia), including many non-Symbiodiniaceae hosting species (Fig. 6b). Further, non-scleractinian *Endozoicomonas* ASVs were often closely related and displayed distinct geographical separation (Fig. 6c).

Of the 4138 *Endozoicomonas* ASVs present in all cnidarian libraries, the majority 74.3% were identified in Scleractinia, 15.8% identified in Alcyonacea, 3.8% to Actinaria, with 3.5% identified in the remaining orders and only 2.6% shared across orders. Of all *Endozoicomonas* ASVs identified within scleractinian corals, 88.5% were exclusively identified in tissue samples, with 5.5% found only in mucus samples, 2.1% in skeletal samples, while 3.9% were shared across body compartments.

Within the V4 library specifically, 1,489 *Endozoicomonas* ASVs were present with 58.4% identified only in a single cnidarian sample, and the majority of minor clades were highly specific to either water body or host family (Fig. 6c). There was a range of host specificity, with some highly specific clades primarily amplified only from specific cnidarian families, including Clade 2 identified only in Indian Ocean Acroporidae colonies, Clade 13 in Pacific Pocilloporidae colonies, and Clade 14 primarily identified in Atlantic Rhizostomeae (Fig. 6b, c). On the other hand, highly generalist clades including Clades 5, 10, and 12 were identified in globally distributed Cnidaria and Porifera. Further, these host generalist clades were more likely to be present across multiple tissue types, compared to the most specialist clades which were generally identified primarily in scleractinian tissue. Clade 11 was highly distinctive, having the greatest average abundance within samples, as well as being identified in the greatest number of cnidarian samples (Supplementary Figs. 18–19). Clade 11 was also a geographic generalist, found globally, and a host specialist, being detected primarily in Octocorallia (Fig. 6c, Supplementary Figs. 18–19).

### Nonbacterial members of cnidarian microbiota

Fewer than 5% of the 720,835 ASVs identified across the five hypervariable libraries were classified as non-bacterial (Supplementary Fig. 13), this is likely because the majority of studies analyzed in our data set aimed to specifically amplify bacteria, and nonbacterial members of the cnidarian microbiota are underreported. Host cnidarian sequences were common, with 47.2% of these identified using the modified Silva dataset and the remaining through BLAST searches. Numerous highly ubiquitous non-bacterial symbionts were more prevalent than previously documented, including Apicomplexan Related Lineages V (ARL-V), that were present in seven cnidarian orders globally, and *Ostreobium* present not only in many scleractinian corals and octocorals, but also in black corals, Antipatharia. A total of 11,995 ASVs (1.5%) were obtained from cnidarians that could not be identified across all hypervariable libraries, even after BLAST searches (Supplementary Fig. 13).

## Discussion

### Cnidarian microbiota diversity

This systematic review revealed that cnidarians host an extraordinary array of microbial phyla (12 archaeal and 74 bacterial phyla, 22 and 185 classes, respectively, Fig. 2). This substantially expands previous totals of the 42 microbial phyla identified in the Coral Microbial Database and the Coral Microbiome Portal^41^, and the 63 bacterial phyla identified in the Sponge Global microbiome dataset^51^. Remarkably, cnidarian microbial phyla diversity also rivals the 85 phyla identified in the Earth Microbiome Project, which examined 2.2 billion free-living and host-associated 16S rRNA sequences^45^. This incredible diversity suggests that cnidarians are one of Earth’s most diverse microbial reservoirs, supporting substantial prokaryotic metacommunities.

### Shared Cnidarian microbiota

The integration of microbiome data across Cnidaria, which we performed in this systematic review, is critical for a greater understanding of cnidarian-microbiome structure and plasticity, as well as prokaryotic interactions with other coral reef invertebrates. Establishing a microbial baseline will also allow for the assessment of stressor-associated dysbiosis. For example, even with the current sampling bias of scleractinian corals, Alcyonacea and Scleractinia each hosted approximately 30% of all shared cnidarian microbiota, suggesting that both orders may act as reservoirs for other cnidarian species (Fig. 4d). Conversely, these shared sequences represented <3% of the entire scleractinian microbiome, of which 12.5% was shared within the order, suggesting that scleractinian corals might not only serve as an important reservoir for scleractinians, but they may also play a key role in the reacquisition of microbiota following stressor-associated dysbiosis.

### Complexity of Cnidarian microbiomes

Across Cnidaria, host phylogeny plays a key role in microbial assembly, influencing the structure, complexity, and diversity of cnidarian microbiomes. Our cnidarian-wide comparisons greatly expand previous findings of phylogenetic influence on coral microbiomes^52–54^, and are similar to processes identified in poriferan holobionts^55,56^. We posit that increased body plan complexity, e.g., the evolution of skeletons in Alcyonacea, Antipatharia, Helioporacea, and Scleractinia, increased the number of internal niches, driving niche partitioning, and facilitating increases in the richness and/or diversity of specific microbial communities (Fig. 3). More diverse microbial communities may lead to diversification of the holobiont phenotype, functional capacities, and metabolic capabilities, thereby enabling expansion of the host into novel habitats^57^. This diversification would help explain the significant changes in microbial richness correlated with the radiation of modern scleractinian families^52^. With Cubozoa, Hydrozoa, Scyphozoa, and Octocorallia hosting significantly less complex microbiomes than Hexacorallia, and less diverse microbiomes than Scleractinia, we propose that less complex microbiomes were the ancestral state of Cnidaria, with more complex microbiomes evolving more recently, similar to that of poriferan holobionts^56^.

### Geographic and phylogenetic influence of Cnidarian microbiomes

We found strong clustering by ocean basin of the microbiomes of closely related cnidarians (Supplementary Figs. 11–12), potentially suggesting that phylosymbiosis (microbial patterns that mirror host phylogeny) may have occurred multiple times in cnidarian holobiont evolution, although this has yet to be tested. While bacterial phylosymbiosis of Scleractinia and Octocorallia has been documented throughout the South-West Pacific^52–54^ we posit that it may have occurred not only throughout Cnidaria, but repeatedly across geographically distinct ocean basins. Additionally, there are significant community signals of cnidarians residing across shallow and deeper depths, with deeper colonies hosting less diverse microbial assemblages. Further, depth strongly influenced microbial dissimilarity not only in Scleractinia, but across Cnidaria.

### Relative abundance of Cnidarian microbiota

Core communities of microbial genera were repeatedly identified across Cnidaria, with their relative abundance primarily influenced by host phylogeny and depth (Fig. 5a). Given that the ‘core microbiome’ has been proposed as important for holobiont resilience^20,58^, we identified smaller proportions of ‘core microbiota’ in shallow water scleractinian corals, that are often more susceptible to environmental changes, than in shallow water Actinarians and Alcyonaceans, which can be more resilient. Shallow Octocorallia exhibited greater microbial richness and higher core relative abundances when compared to phylogenetically older and deeper lineages, suggesting that these core microbiomes were repeatedly assembled across shallow water lineages^57,59^. Surprisingly, tissue core microbiomes (averaged to cnidarian family) consisted of only 0–10 ASVs that were present in all colonies per site (Fig. 5b). The taxonomic identity of these ASVs was highly conserved, exhibiting depth and cnidarian order fidelity, suggesting potentially important functional roles for these microbial families across Cnidaria (Fig. 5c). This expands previous findings that scleractinian microbiomes are highly individualistic^60,61^, and are dominated by rare sequences^23,62^, suggesting that this finding is common for many cnidarians, who potentially exhibit a high degree of functional redundancy across their stable microbial component.

### Prevalence and specificity of *Endozoicomonas*

Of all microbial families detected across Cnidaria, *Endozoicomonas* was not only the most prevalent, it was also one of the most ubiquitous globally and consistently present at different depths (Figs. 2−5). Therefore, *Endozoicomonas* may play an important role in healthy cnidarian microbiomes. *Endozoicomonas* are metabolically versatile^63–65^ and may translocate vitamins to their host^66^. While *Endozoicomonas* occur in close proximity to Symbiodiniaceae in coral tissue^67^, 21.4% of the *Endozoicomonas* sequences in our dataset were identified from cnidarian samples that did not host Symbiodiniaceae, with 28.7% of all sequences identified isolated from non-scleractinian samples. Further, comparing the Illumina MiSeq subset of sequences (V4 library), demonstrated that octocorals in the Caribbean, Mediterranean, and Indo-Pacific not only hosted the greatest absolute abundance of *Endozoicomonas*, but these sequences were shared across more samples, compared to any other cnidarian. Both specialist and generalist strains of *Endozoicomonas* exist within Anthozoa, suggesting a variable genetic component that has the potential for coevolution with their hosts^41,52–54,62^ similar to that of Symbiodiniaceae^68^. We expanded this observation and revealed patterns of switching in host specificity between Anthozoa and non-Anthozoa (including Cubozoa, Hydrozoa, Scyphozoa, and Porifera; Fig. 6a), as well as within Anthozoa, specifically forming multiple highly conserved associations with Alcyonacea and Rhizostomeae (Fig. 6b), in addition to fidelity to body compartment and ocean body (Fig. 6c).

### Potentially beneficial core microbiota

We identified additional common core microbiota across both tissue, skeletal, and mucus samples with potential functional importance. *Pseudoalteromonas*, a genus well-known as a producer of antimicrobials^17^, exhibits co-phylogeny with Scleractinia, as does *Alteromonas*^62^. Spirochaetes dominate some octocoral communities and may be involved in nutrient cycling^69^. While these bacteria have been previously identified as being relatively abundant or core members of many scleractinian and octocoral species, here we demonstrate that other cnidarians host these bacteria, hinting at their potential functional importance across Cnidaria. Additionally, we identified other bacteria that may be of functional importance but require further examination. For example, *Synechococcus* and *Prochlorococcus*, in the cyanobacterial genus GpIIa, commonly occur in cnidarian microbiomes^60^, as well as being abundant in marine water columns^70^, and therefore may be a sampling artifact. It is also important to experimentally determine whether *Propionibacterium* is a naturally abundant microbe or a sequencing kit contamination^49,50^. Although, by comparing these core microbiota to commonly found methodological contaminants i.e., *Propionibacterium*, we have increased the confidence that these bacterial genera may not be contaminants.

### Potentially detrimental core microbiota

In this study, we analyzed microbiomes sampled from putatively ʻhealthyʼ cnidarians, focusing on original datasets that did not mention observation of stress or anthropogenic disturbance(s), although this does not discount the possibility that some data may have come from stressed corals or emerging dysbiosis. Many of the bacterial genera that appeared as core members of ‘healthy’ tissue and skeletal microbiomes have been connected to anthropogenic stress, or are potential disease-causing microbes, including *Vibrio, Mycoplasma, Sphingomonas*, *Candidatus* Pelagibacter, and *Pelomonas*^34,71–74^. The presence of these genera was significantly increased with clustered sequence identification, indicating greater variability in these bacteria between samples. The prevalence and abundance of these microbes could potentially indicate non-virulent symbioses that could become rapidly virulent depending on microbe-microbe competition or fluctuation^74,75^, or that many phenotypically ʻhealthyʼ corals are experiencing underlying dysbiosis^76^.

### Symbiodiniaceae microbiota

Few of the microbiota from cultured Symbiodiniaceae also occurred in the cnidarian samples. *Pseudomonas*, for example, which was identified as a core bacterial genus in cnidarians, also occurred as a core intracellular microbe of Symbiodiniaceae^77^. *Pseudomonas* co-occurs with Symbiodiniaceae globally^78^, indicating a functional, or conserved symbiosis with both cnidarians and Symbiodiniaceae. This could explain the coculturing benefits of Symbiodiniaceae with *Pseudomonas* in laboratory studies^79^. Conversely, most other microorganisms that were identified as being part of the core microbiome of cultured Symbiodiniaceae^77,80,81^ were not detected in cnidarian samples. Potentially, the microbiomes of cultured algae differ from those of non-cultured Symbiodiniaceae, or there may be a minimal introduction of novel bacteria through the acquisition of Symbiodiniaceae on coral reefs. This incongruity reiterates the urgency for exploration into Symbiodiniaceae phycospheres and microbiomes^82,83^, especially given the critical role of Symbiodiniaceae in the survival of many cnidarians.

### Summary

This study provides important and fundamental insights into our understanding of how cnidarian microbiomes are structured globally. Cnidarian microbiomes are incredibly complex and integrating datasets can potentially enable identifying specific prokaryotic communities to focus future research on their functional role in cnidarian holobionts. The analysis also expanded our knowledge of prokaryotic communities across non-scleractinian cnidarians, which complements our understanding of the role and relevance of microbes within scleractinian corals. Because our data analysis demonstrated that clustering-based sequence pipelines are variable and depend on the cnidarian and microhabitat examined, we recommend that future cnidarian microbiome studies utilize unique sequence analysis. The current review focused primarily on a subset of the available data. Utilizing the entire database requires future investigations into the potential bias in sample storage, sequencing, and processing protocols. Given the current threats posed to cnidarians worldwide, our study provides a global, speciose, microbiome baseline for ‘healthy’ corals. With this baseline, we can begin to elucidate the impacts of stress-associated dysbiosis, in addition to addressing a myriad of ecological questions regarding the structuring of cnidarian microbiomes.

## Methods

### Sample and metadata collection

A literature search was conducted to obtain culture-independent 16S rRNA gene sequences available from any cnidarian. The literature search culminated in August 2021 with 257 datasets which identified sequences of the partial or full 16S rRNA genes of the microbial community from at least one cnidarian species (Supplementary Fig. 20). An additional seven projects had partially sequenced the 16S rRNA gene from cultured Symbiodiniaceae, seawater samples, and a standardized collection of globally distributed Porifera. Of the 257 studies, 88 contained sequence data that were either not publicly available, were multiplexed without index tags, lacked critical metadata, or could not be located online. Communication with corresponding authors resulted in 23 of these datasets being made available, which we gratefully acknowledge. From the 192 available data sets, 15 were further excluded due to sequencing files that could not be matched to their original samples, or continued to lack critical metadata.

The remaining 177 datasets provided 12,010 cnidarian associated 16 S rRNA sequencing files from 212 cnidarian species (Fig. 1, Supplementary Data 1), with an additional 54 sample groups identified at a classification broader than species. Microbiome sequences from cnidarians included samples from members of all cnidarian classes: Anthozoa, Cubozoa, Hydrozoa, and Scyphozoa, and 12 orders (Actinaria, Alcyonacea, Anthoathecata, Antipatharia, Carybdeida, Corallimorpharia, Helioporacea, Leptothecata, Rhizostomeae, Scleractinia, Semaestomeae, and Zoantharia). Nevertheless, sampling was highly biased toward Scleractinia (8754 samples), whereas Alcyonacea (1431), Actiniaria (1305) and the remaining orders (520) had far fewer samples (Supplementary Data 1).

Of the sequenced microbiome samples, 7575 were collected from cnidarians at 0–15 m depth, 473 from 16–30 m depth, 505 from 31–150 m depth, and 834 from >150 m depth (Supplementary Data 1). Geographically, microbiome samples were predominantly obtained from the South Pacific Ocean (3777), the North Atlantic Ocean (3376), the Northwest Pacific Ocean (1204), the Indian Ocean (1111), the Mediterranean Sea (799), and the remaining from nine additional water bodies (Fig. 1, Supplementary Data 1).

To compare cnidarian microbiomes to the microbiomes of their mutualistic endosymbiotic algae (family Symbiodiniaceae), and the environment (water column), we also integrated 4003 microbial sequence files from seawater, poriferan, and cultured Symbiodiniaceae samples, resulting in a final dataset of ~6.5 billion sequences. Furthermore, the inclusion of ‘The Sponge Microbiome Project’^51^ allowed us to compare across non-bilaterian marine invertebrates that often cohabitate the same reefs.

Up to 33 parameters from metadata, including host, environmental, sequencing, and experimental variables, were collected for each sample, either from the original publication, supplementary information, or sequencing data (Supplementary Data 1).

### Sequence curation

FastQ files were retrieved from online databases, primarily from Genbank (http://www.ncbi.nlm.nih.gov/genbank) and the European Nucleotide Archive (http://www.ebi.ac.uk/ena), or directly provided by the published papers’ authors. The majority of these were sequenced using an Illumina MiSeq platform (12,808), with most downloaded in their raw unmerged format (8022), followed by 454 (1216), Illumina HiSeq (651), and additional platforms (1338). Idemp^84^ was used to demultiplex files where required. Given the variety of sequencing platforms, each file was initially previewed with FastQC (v0.11.19)^85^ to determine sequence quality scores and to locate any remaining barcodes, adaptors, or primers, which were subsequentially removed with Trimmomatic (v0.39)^86^ where present.

### Creation of shared sequence libraries

All edited sequences were visually observed and aligned to a full-length *Escherichia coli* sequence (Accession no. J01859) with GeneiousPrime (build 2020-11-05). From this, overlapping hypervariable regions were mapped across the entire dataset and the ranges for five cnidarian and one that included poriferan sequences (V4_p) 16S rRNA libraries were generated: V1-V2 (30-360 bp, 1949 samples), V3 (350–500 bp, 2106 samples), V4 (520–780 bp, 5979 samples), V5-V6 (820–1050 bp, 3751 samples), V7-V8 (1040–1400 bp, 469 samples), and V4_p (550–660 bp, 9737 samples). Sequences from each study were filtered and trimmed separately using the DADA2 R-package (v1.18-1.22)^87^ in RStudio (v1.4.1103-4)^88^, allowing for the calculation of a distinct error rate per study. Single and merged Illumina sequences, 454, Ion Torrent, and PacBio sequences were all denoised with unique parameters, according to the DADA2 SOP (https://benjjneb.github.io/dada2/tutorial.html). All sequences were processed with the filtering qualifications of (maxN = 0, maxEE = 4, truncQ = 10), merged if appropriate, sequence tables constructed, and chimeras removed. Once sequence tables for each project had been created within a library, they were collapsed together before taxonomic classification with a naïve Bayesian method using DADA2. A total of 720,835 ASVs were present across the five cnidarian libraries, with 493,157 ASVs present in the V4_p library. Sequences were then merged with their metadata and exported using the ‘phyloseq’ R-package (v1.38.0)^89^ for further analysis.

To increase the detection rate of host mitochondrial sequences, the Silva training dataset (version 138.1^90^) was adapted by inserting 133 partial and full-length mitochondrial sequences representing 82% of the cnidarian and 60% of the poriferan genera found in this study^91^. Additional sequences were added to further identify recently identified cnidarian symbionts, including 120 sequences from eukaryotic ARL (Apicomplexan-Related-Lineages)^92,93^, and 12 from ‘*Candidatus* Aquarickettsia rhoweri’^72^.

### Non-archaeal and bacterial sequences ASVs

ASVs classified as “Eukaryote”, “Mitochondria”, “Chloroplast”, or unassigned at the kingdom level, were removed from all six shared libraries and from each core microbiome dataset. To test the suitability of this common contamination removal process, the 34,220 ASVs excluded from the five cnidarian libraries, were investigated using BLAST + ^94^. A local megablast search was conducted, with the top hit automatically chosen for sequences above 90% sequence similarity (Supplementary Fig. 13).

### Analyzing the structure and diversity of cnidarian microbiomes

As the V4 library included the greatest abundance of cnidarian specific sequences (360,006), and the broadest phylogenetic variability (11 cnidarian orders) of samples in our dataset, it was selected for further analysis. Sequences were aligned with the ‘DECPIHER’ R-package v2.22.0^95^, subset to family and used to generate phylogenetic trees. These neighbor-joining trees were optimized with a GTR + G substation model (model determined by modelTest) to estimate a maximum-likelihood trees, using the ‘*phangorn’* R-package (v2.8.1)^96^ and merged into phyloseq objects with the “ape” R-package (v5.6-1)^97^. Prevalence of microbial families and relative abundances within the four cnidarian classes were plotted and visualized (Fig. 2) using the ‘ggtree’ R-package (v3.2.1)^98^.

Given the current debate concerning the applicability of unrarefied vs. rarefied data^99,100^, we conducted our analyses on both an unrarefied and rarefied (minimum 5000 reads) V4 library, subset only to ‘healthy’, non-experimental samples sequenced on a MiSeq (4243, and 2964, respectively). From these samples, four additional subsets were created and tested, including only scleractinian, and all non-scleractinian orders.

Shannon diversity, ASVs observed, and Inverse-Simpson metrics were tested on these three data subsets with a linear mixed-effects model, treating the original study as a random blocking effect, using the ‘lme4’ R-package (v1.1-27.1)^101^. R^2^ values corrected with false discovery rates were then calculated with the ‘*emmeans’* R-package (v1.7.2)^102^ and the *MuMIn* R-package (v1.43.17)^103^. Significant factors were further tested with Kruskal Wallis analyses, accompanied by Dunn post hoc tests and Bonferonni corrections (Supplementary Figs. 1–2, 4–8) using the ‘dunn.test’ R-package (v1.3.5)^104^, and the ‘ranacapa’ R-package (v0.1.0)^105^. Rarefaction plots were created using the ‘*vegan’ R-*package (v2.5-7)^106^, and the Shannon diversity was plotted (Fig. 3), with the ‘ggplot2’ R-package v3.3.5^107^. Extrapolation of diversity was conducted using the ‘*iNEXT’* R-package (v3.0.0)^108^

Bray-Curtis and Jaccard dissimilarity indices and dispersions were calculated for the three unrarefied and rarefied subsets of the V4 library, using a PERMANOVA (999 permutations) with the original study treated as a random blocking effect (Supplementary Tables 1–6), using the ‘*vegan’ R-*package v2.5-7^106^ (Supplementary Figs. 9–10). Both the V4 and V4_p libraries were hierarchically clustered (complete linkage) using Bray-Curtis dissimilarities and plotted with the *‘stats’* R-package (v4.1.2)^88^.

### Identifying the shared and distinctive microbiota

The absolute abundance of ASVs from each of the five cnidarians libraries that were shared across cnidarian phyla, individual microhabitat, depth, and ocean bodies were calculated and visualized (Fig. 4a–c) with the *‘*MiscMetabar’ R-package (v0.21)^109^, while shared sequences between cnidarian orders were calculated and visualized (Fig. 4d) using the ‘circlize’ R-package (v0.4.13)^110^.

### Creation of core microbiome datasets

A total of 143 studies sequenced a minimum of four ‘healthy’ (no record of observed stress) cnidarian (tissue, skeletal, mucus) samples per species per sampling site, collected from ambient conditions (no environmental or experimental stressors documented). The raw untrimmed sequences from these studies were reprocessed separately using DADA2. While the denoising parameters were the same as above, these sequences were not merged with other studies, allowing for the analysis of their full length of sequences, and sequence tables were classified separately. Sequences were then merged with their metadata and exported using the ‘phyloseq’ R-package for further analysis.

### Calculating variability in the ‘core’ microbiota

The 588,776 ASVs from the core microbiota sequence tables were additionally clustered into 198,710 ASVs_97_ sequences with 97% similarity (approximating OTUs) for comparison, using the ‘DECPIHER’ R-package v2.22.0^95^. Both ASV and ASVs_97_ sequences from each of the 143 sequence tables were then tested for the relative abundance of shared sequences across each cnidarian species at each sampling site. Sequences were determined as being present across sampled individuals, including every sample per site (100%), more than 75%, more than 50%, less than 50%, and only in a single cnidarian individual (Fig. 5a), using the ‘microbiome’ R-package (v1.16.0)^111^. These values were averaged per site for each cnidarian species and were averaged again within cnidarian families. Kruskal Wallis analyses tested the relative abundances of bacteria against the cnidarian order, symbiotic status, and across sampling depth, and were accompanied by Dunn post hoc tests followed by Bonferroni corrections. The absolute abundance of ASVs present in 100% of samples (Core ASVs) per cnidarian species was averaged across families and plotted (Fig. 5b) using the ‘ggplot2’ R-package^107^. Further, the identities of the ASV and ASVs_97_ identified in 100% of individuals, and greater than 75% of individuals of at least one cnidarian species was also visualized (Fig. 5c). Potential PCR and kit contaminants were identified and removed using the ‘decontam’ R-package v1.14.0^112^.

### *Endozoicomonas* specificity, abundance, and fidelity in Cnidaria

*Endozoicomonas* sequences and abundances were isolated from all six libraries using the ‘phyloseq’ R-package^89^, aligned with the ‘DECPIHER’ R-package v2.22.0^95^ and were used to generate phylogenetic trees as previously detailed. The absolute and average abundances of *Endozoicomonas* sequences for each clade were plotted against the average number of samples that *Endozoicomonas* were identified within the phyla Cnidaria and Porifera (Fig. 6a), within cnidarian orders (Fig. 6b), and within water bodies and whether the *Endozoicomonas* was host specific or generalist (Fig. 6c).

### Supplementary information

Supplementary Information

Peer Review File

Description of Additional Supplementary Files

Supplementary Data 1

## Data Availability

All six 16S rRNA gene libraries generated in this study (V1-V2^113^, V3^114^, V4^115^, V5-V6^116^, V7-V8^117^, V4_p^118^) have been deposited in the ‘Cnidarian Microbiome Database Project’ at Figshare (https://figshare.com/projects/Cnidarian_Microbiome_Database/158957), alongside the cnidarian specific RDP annotation file. All original metadata are provided in Supplementary Data 1, alongside sequence accession numbers for each sample, where available.
