# Supplementary material for: Systematic review of cnidarian microbiomes reveals insights into the structure, specificity, and fidelity of marine associations
Source: Nat Commun. 2023 Aug 14;14:4899. doi: 10.1038/s41467-023-39876-6 (PMC10425419; doi:10.1038/s41467-023-39876-6)
Supplement: Supplementary file 1 — Supplementary Information [file 41467_2023_39876_MOESM1_ESM.pdf]

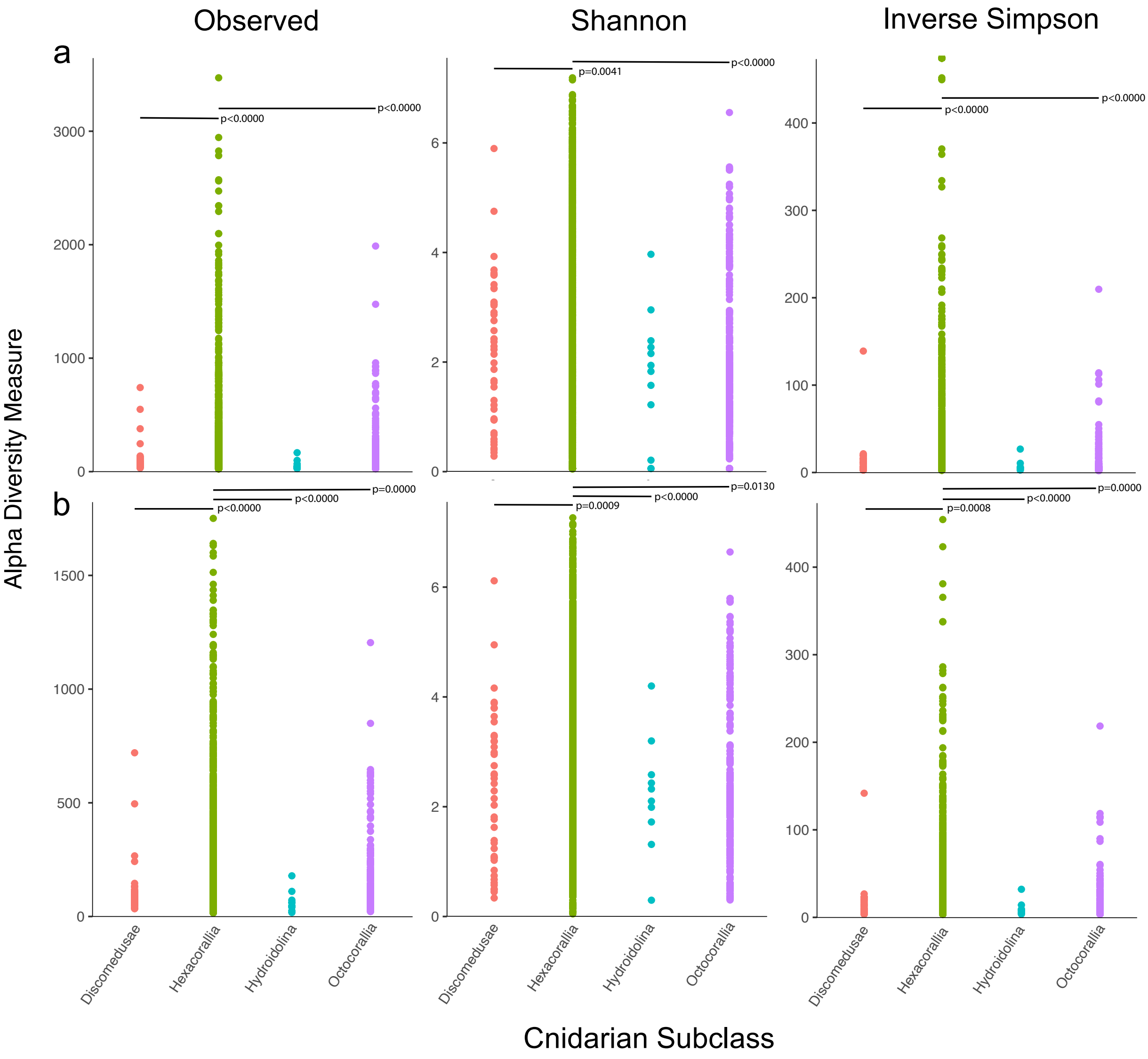

Supplementary Figure 1. Alpha diversity indices for cnidarian subclasses with a, unrarefied, and b, rarefied (minimum 5000 reads) Illumina Mi-Seq samples (V4_MiSeq_healthy_cnidarian library). Lines indicate significant differences between cnidarian subclasses after performing a Kruskal-Wallis test with Dunn's post hoc test (p adjusted<0.02).

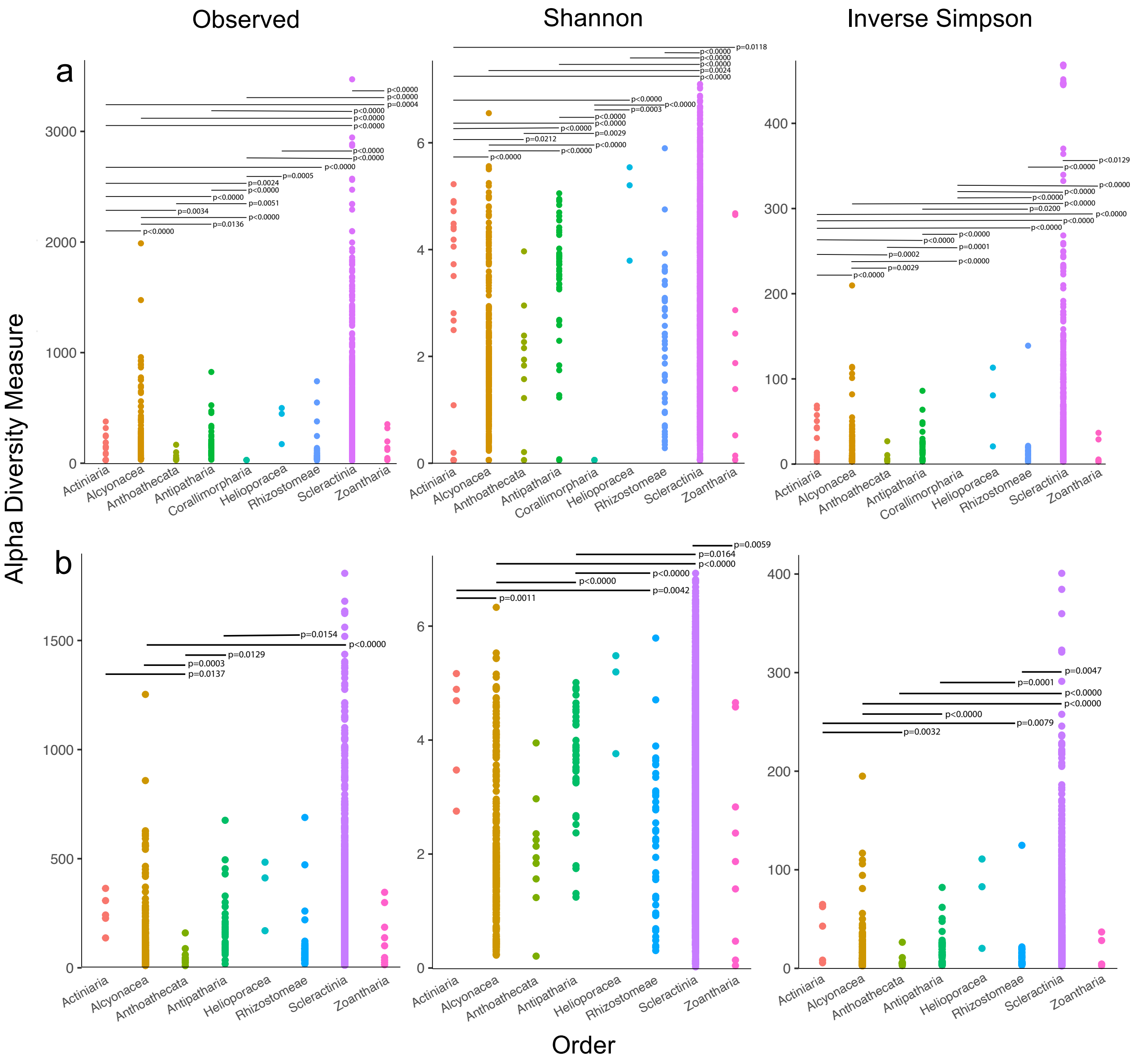

Supplementary Figure 2. Alpha diversity indices for cnidarian orders with a, unrarefied, and b, rarefied (minimum 5000 reads) Illumina Mi-Seq samples (V4_MiSeq_healthy_cnidarian library). Lines indicate significant differences between cnidarian orders after performing a Kruskal-Wallis test with Dunn's post hoc test (p adjusted<0.02).

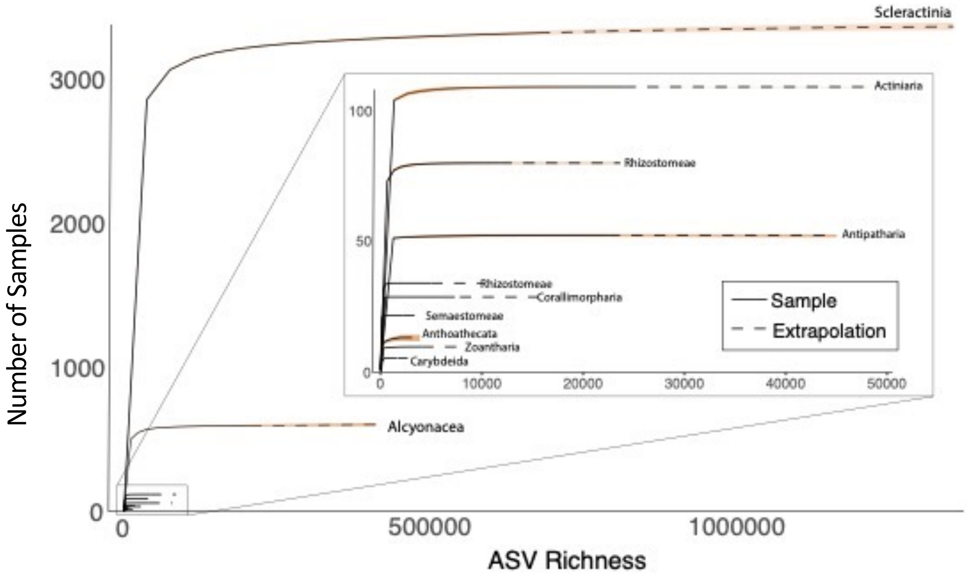

Supplementary Figure 3. Amplicon sequence variant (ASV) richness for samples within the V4_MiSeq_healthy_cnidarian library (solid lines), alongside extrapolated increasing sampling efforts (dotted lines) and 95% confidence intervals (yellow lines).

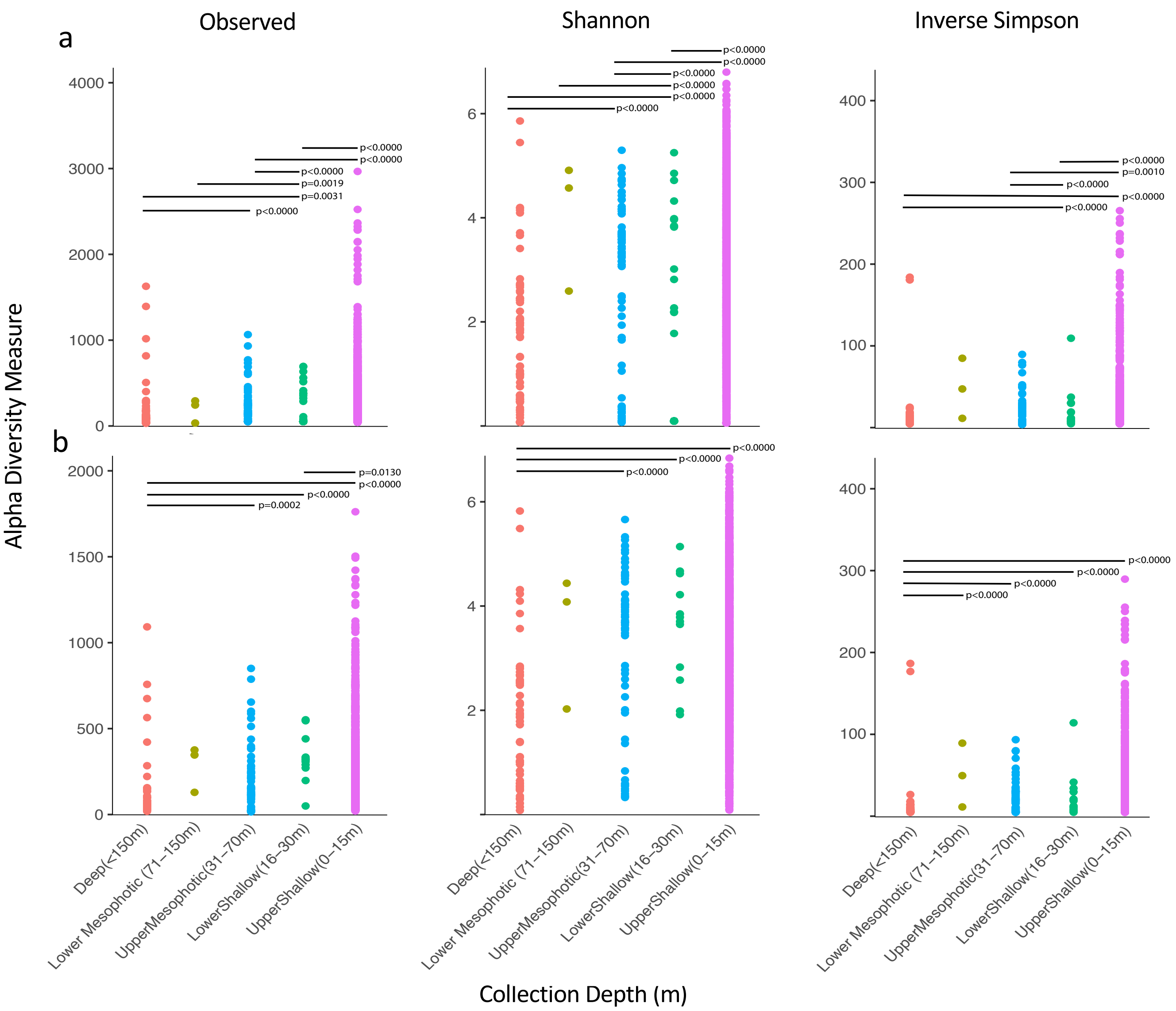

Supplementary Figure 4. Alpha diversity indices for cnidarian samples collected across various depths with a, unrarefied, and b, rarefied (minimum 5000 reads) Illumina Mi-Seq samples (V4_MiSeq_healthy_cnidarian library). Lines indicate significant differences between depths after performing a Kruskal-Wallis test with Dunn's post hoc test (p adjusted<0.02).

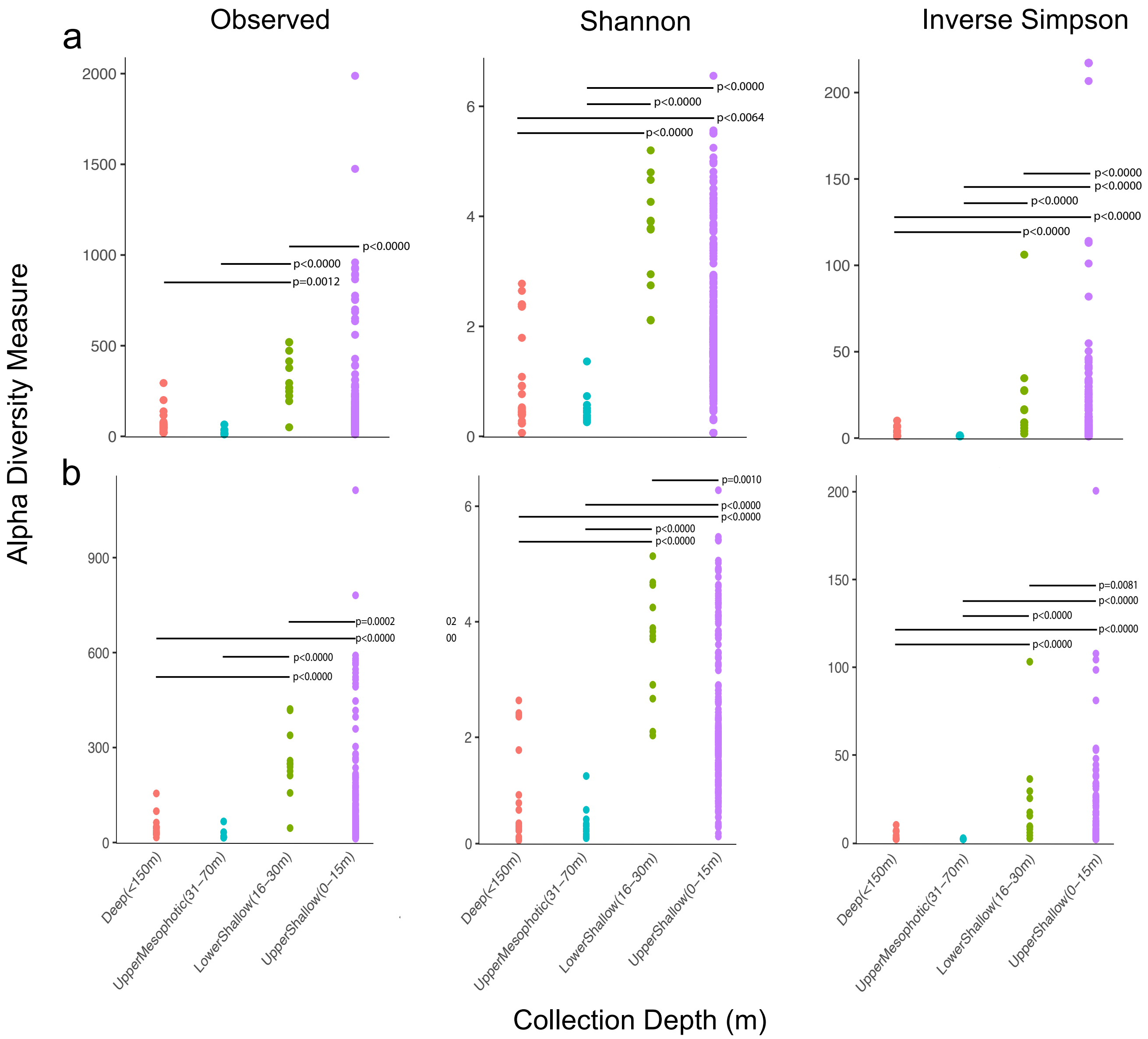

Supplementary Figure 5. Alpha diversity indices for alcyonacean samples collected across various depths with a, unrarefied, and b, rarefied (minimum 5000 reads) Illumina Mi-Seq samples (V4_MiSeq_healthy_cnidarian library). Lines indicate significant differences between depths after performing a Kruskal-Wallis test with Dunn's post hoc test (p adjusted<0.02).

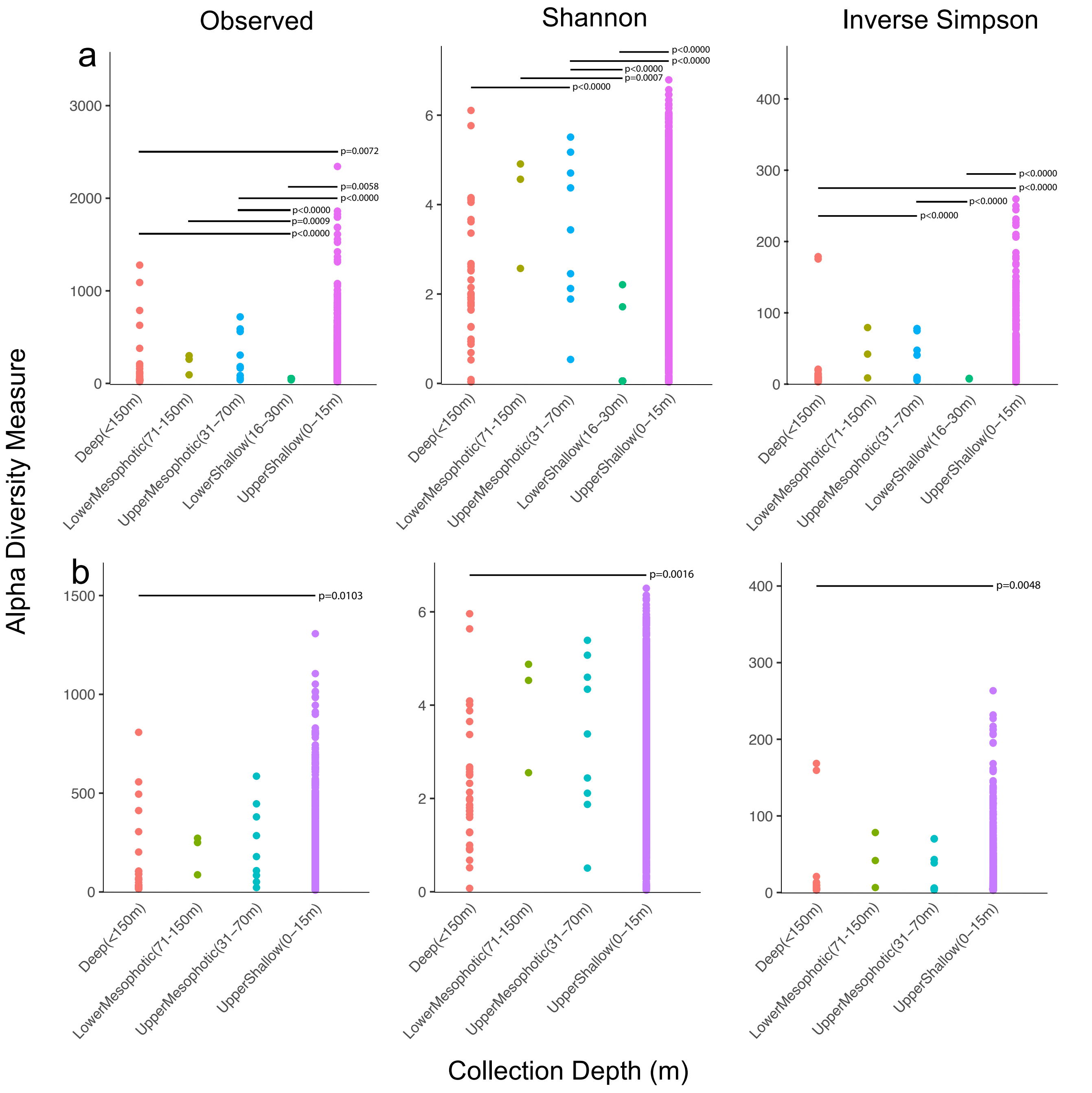

Supplementary Figure 6. Alpha diversity indices for scleractinian samples collected across various depths with a, unrarefied, and b, rarefied (minimum 5000 reads) Illumina Mi-Seq samples (V4_MiSeq_healthy_cnidarian library). Lines indicate significant differences between depths after performing a Kruskal-Wallis test with Dunn's post hoc test (p adjusted<0.02).

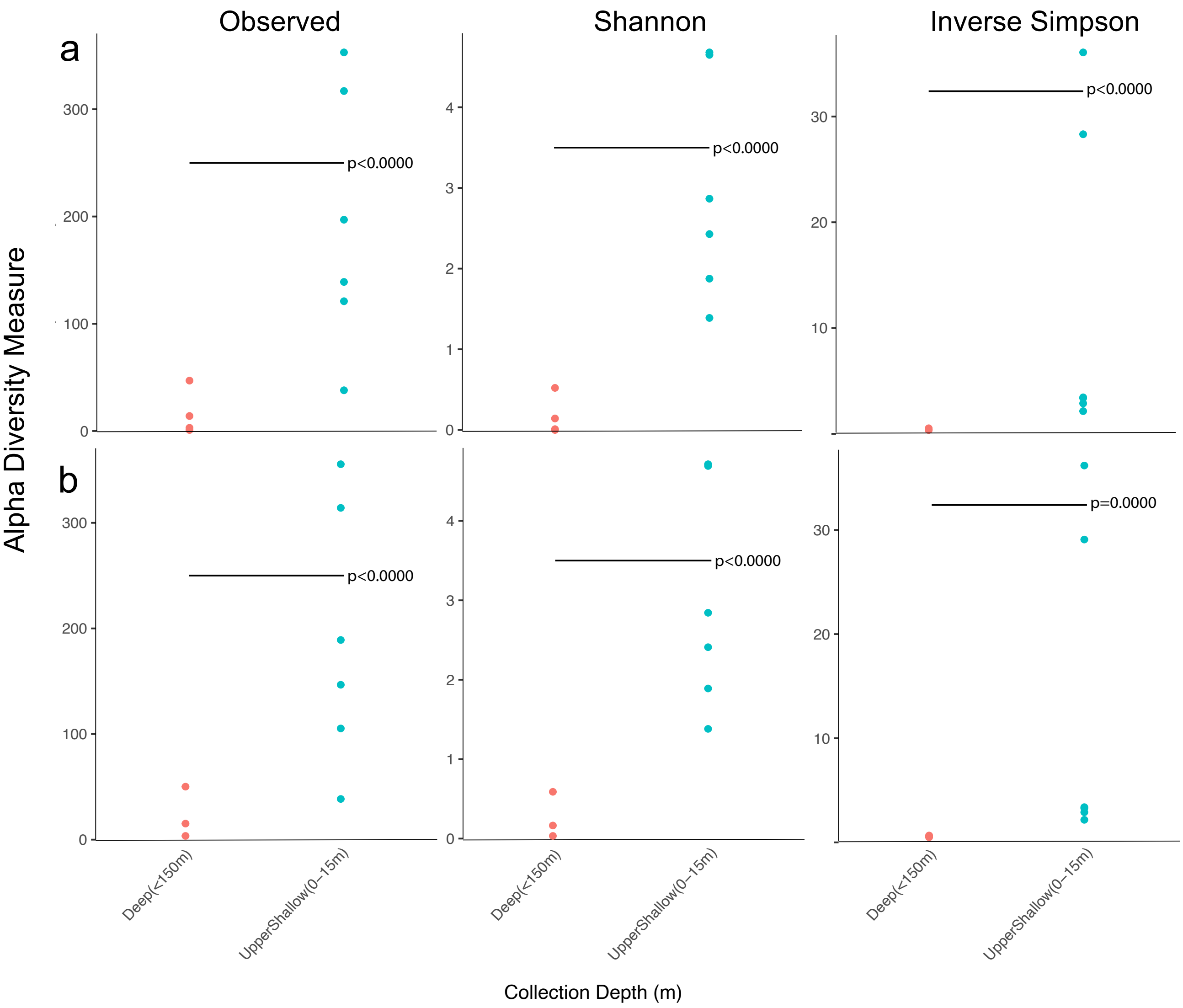

Supplementary Figure 7. Alpha diversity indices for zoantharian samples collected in upper shallow (0-15m) and deep (<150 m) depths with a, unrarefied, and b, rarefied (minimum 5000 reads) Illumina Mi-Seq samples (V4_MiSeq_healthy_cnidarian library). Lines indicate significant differences between depths after performing a Kruskal-Wallis test with Dunn's post hoc test (p adjusted<0.02).

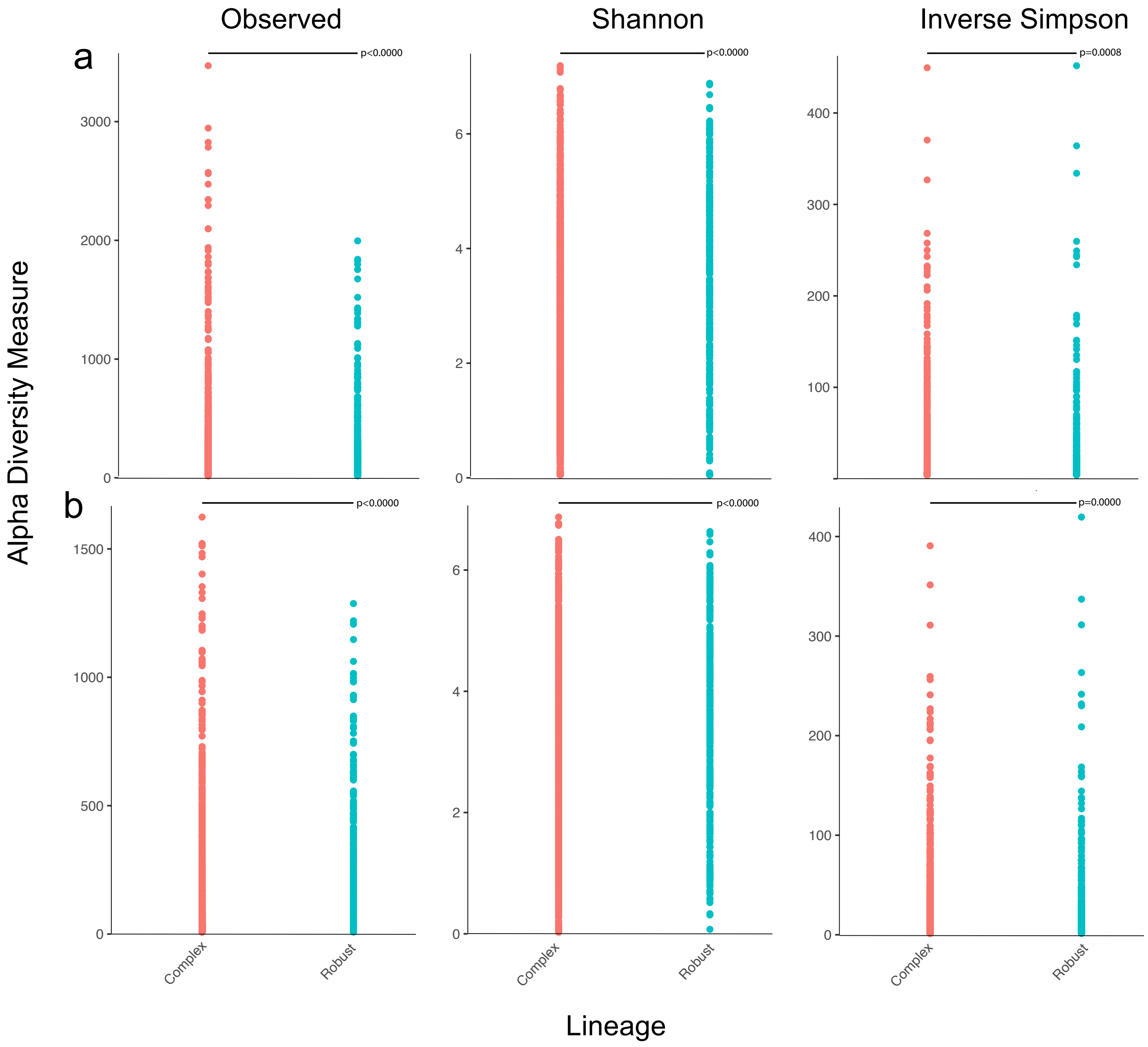

Supplementary Figure 8. Alpha diversity indices for scleractinian lineages with a, unrarefied, and b, rarefied (minimum 5000 reads) Illumina Mi-Seq samples (V4_MiSeq_healthy_cnidarian library). Lines indicate significant differences between lineages after performing a Kruskal-Wallis test with Dunn's post hoc test (p adjusted<0.02).

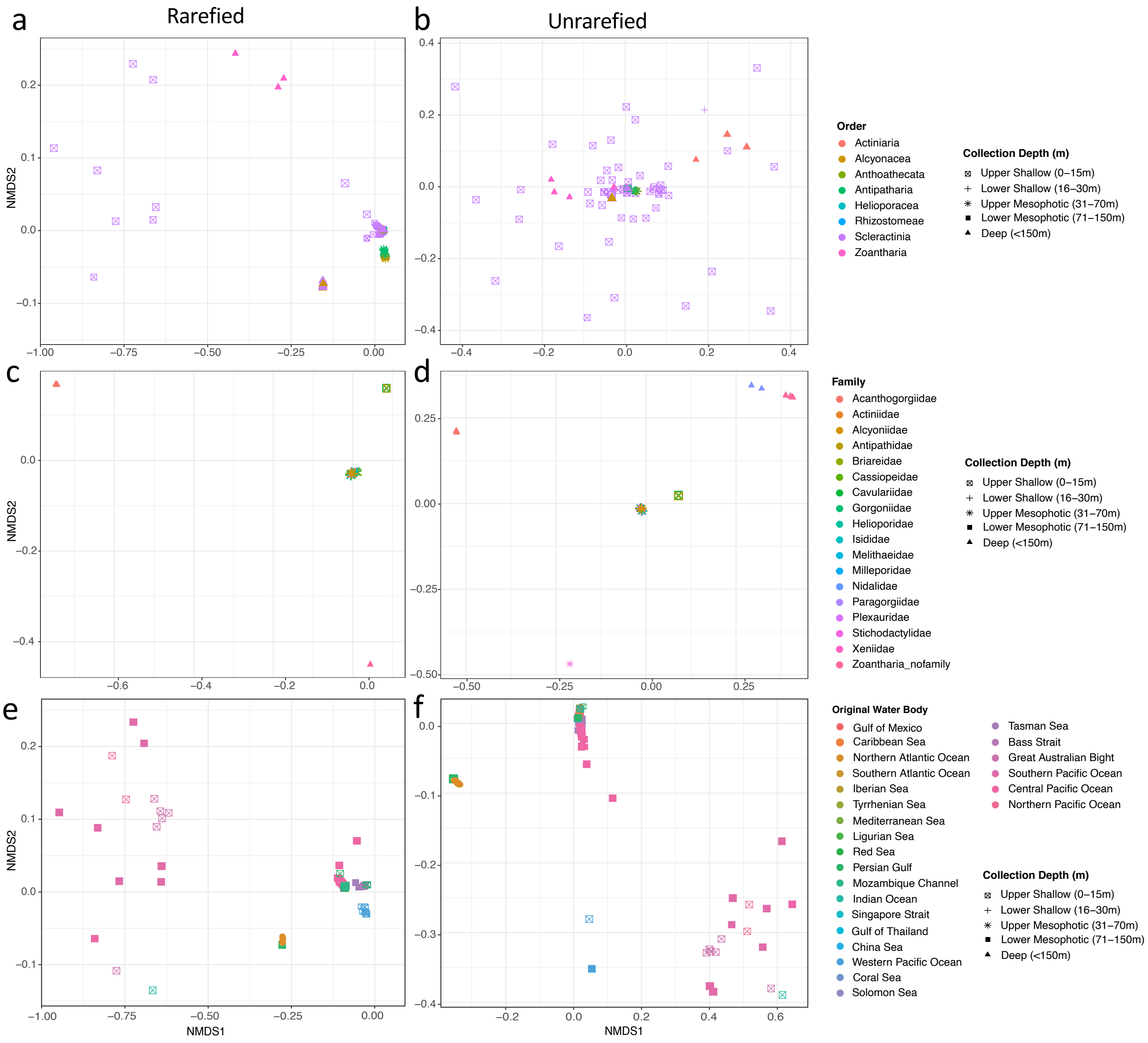

Supplementary Figure 9. Bacterial beta diversity (V4_MiSeq_healthy_cnidarian library), shown as non-metric multidimensional scaling ordination based on Bray-Curtis dissimilarity between a,b, cnidarian orders, c,d, non-scleractinian cnidarian families, e,f, sampling locations for scleractinian samples. Sequences were rarefied (a,c,e), or unrarefied (b,d,f).

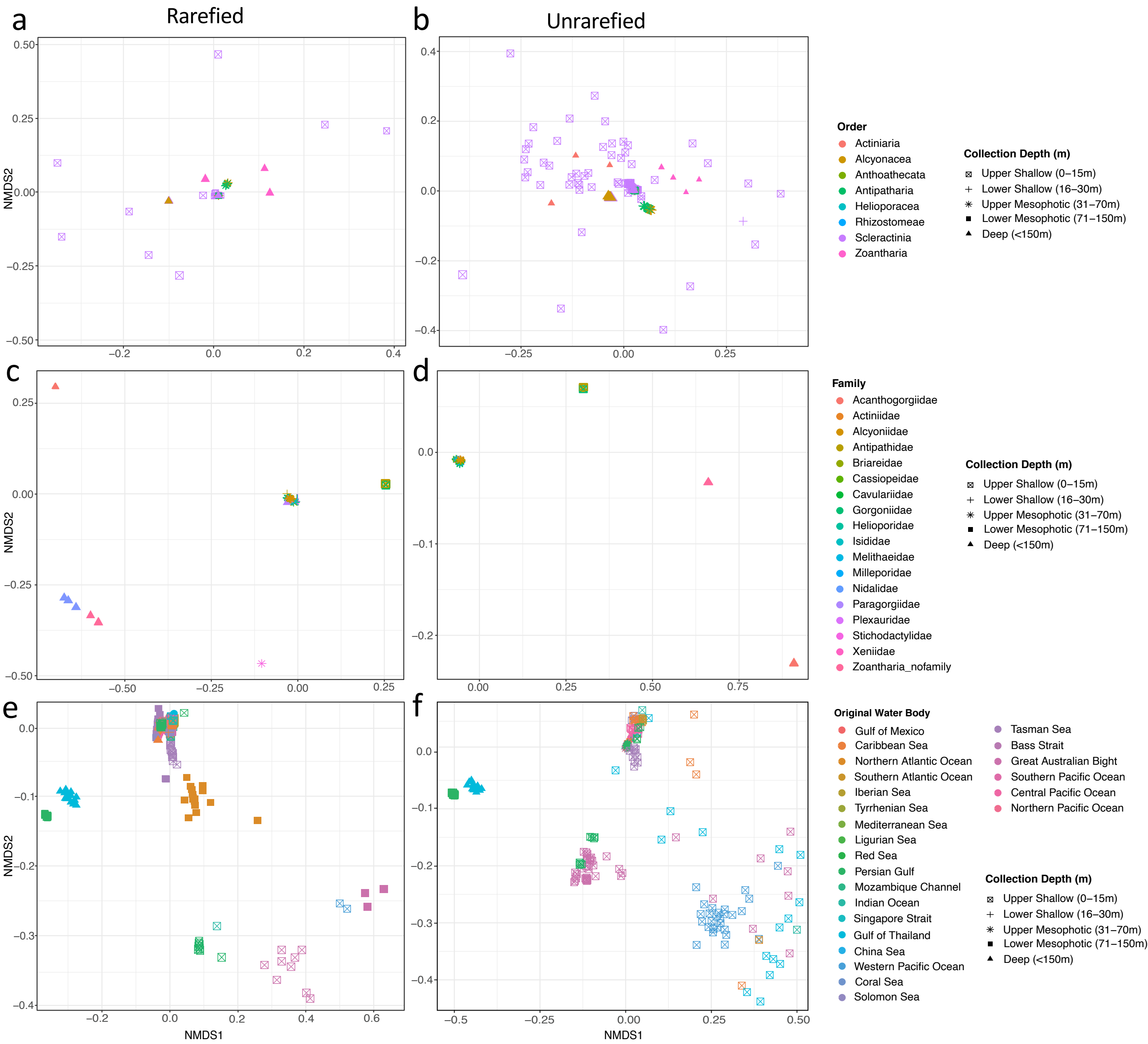

Supplementary Figure 10. Bacterial beta diversity (V4_MiSeq_healthy_cnidarian library), shown as non-metric multidimensional scaling ordination based on Jaccard dissimilarity between a,b, cnidarian orders, c,d, non-scleractinian cnidarian families, e,f, sampling locations for scleractinian samples. Sequences were rarefied (a,c,e), or unrarefied (b,d,f).

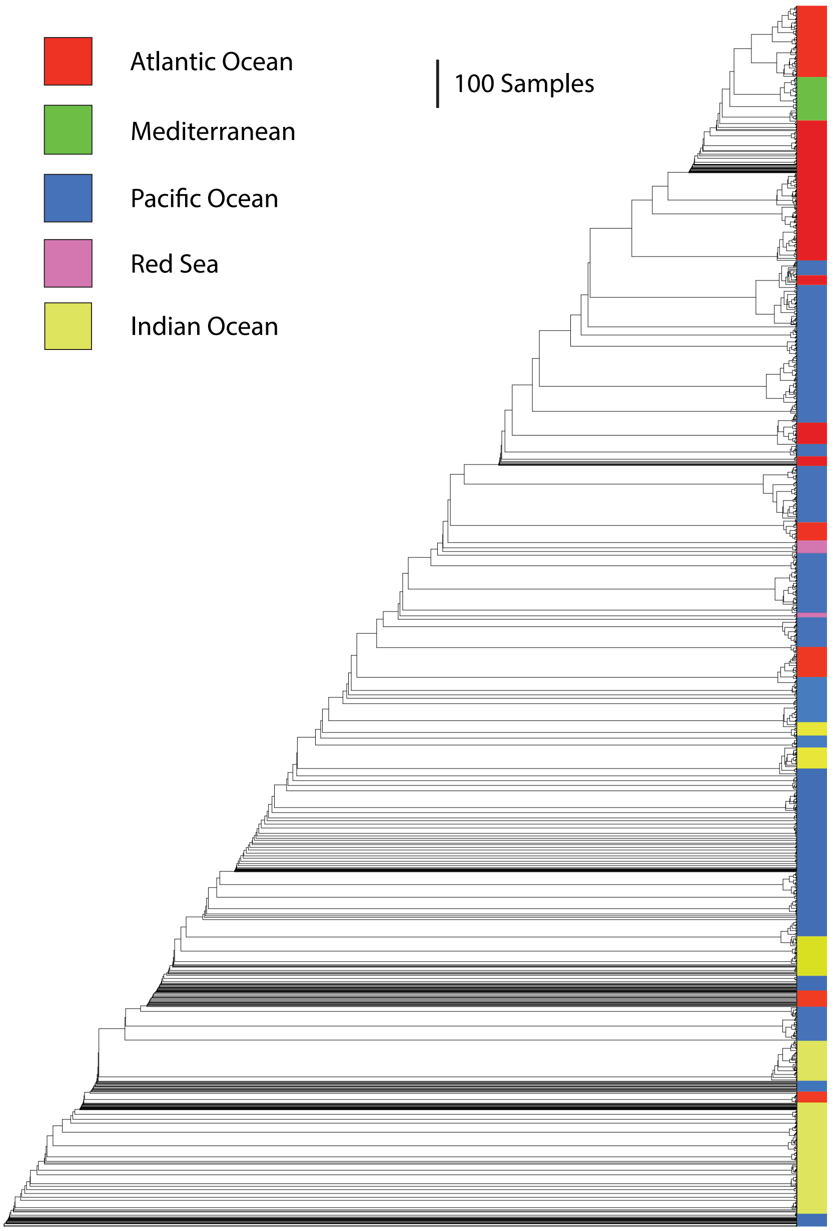

Supplementary Figure 11. Hierarchical clustering of the Bray-Curtis dissimilarity for 16S rRNA samples for 2012 'healthy' scleractinian colonies (V4_MiSeq_healthy_scleractinian). Colored bars group samples with a minimum of 75% of samples from each ocean body. Dendrogram is rooted for visual purposes only

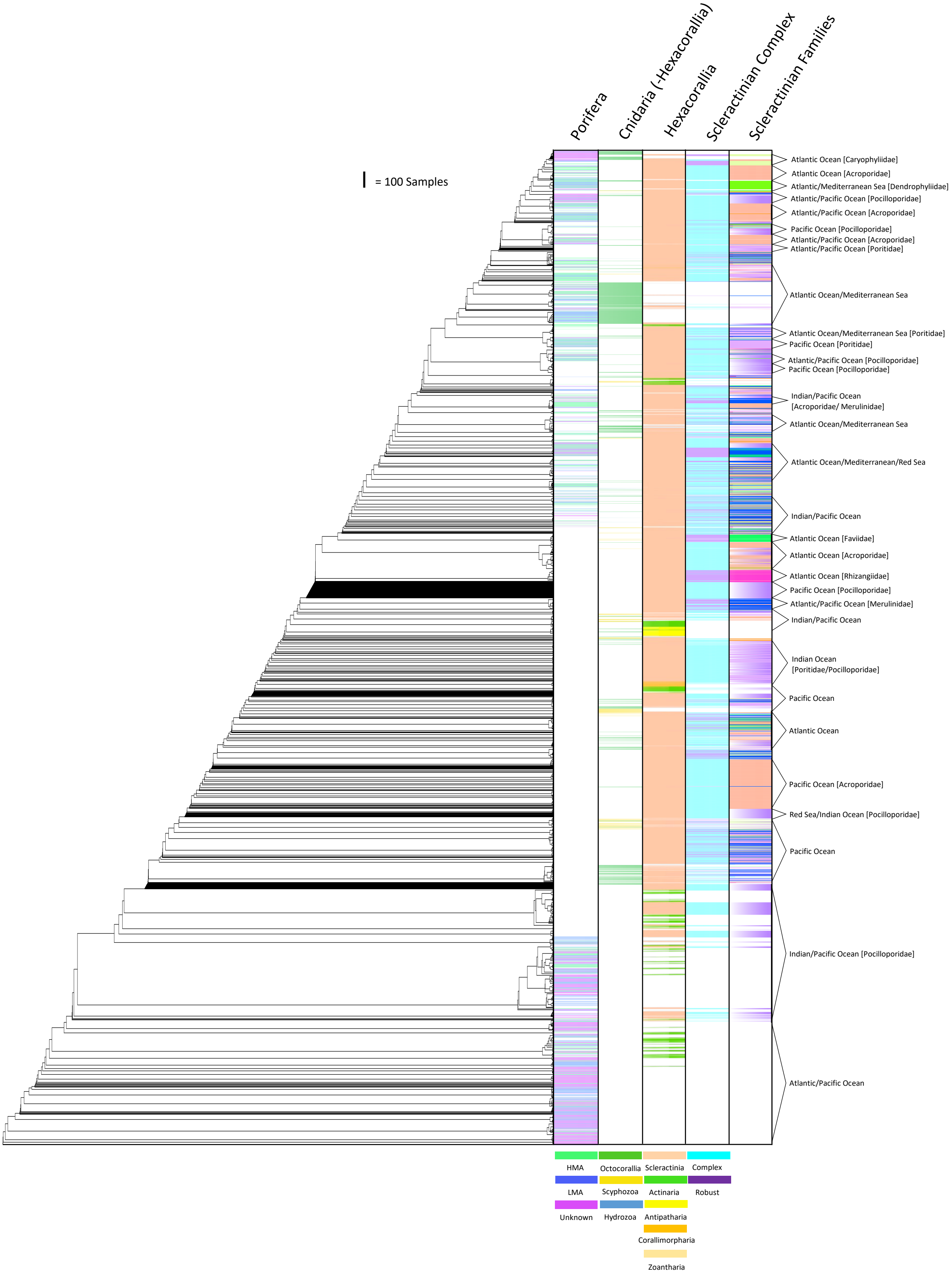

Supplementary Figure 12. Hierarchical clustering of the Bray-Curtis dissimilarity for cnidarian and porifera 16S rRNA gene samples (V4_p library). The first column represents Porifera, the second non-Hexacorralia cnidarians, the third Hexacorallia samples, the fourth scleractinian complexes, and the fifth scleractinian families. Labels represent at least 75% of samples from that location/family. Dendrogram is rooted for visual purposes only.

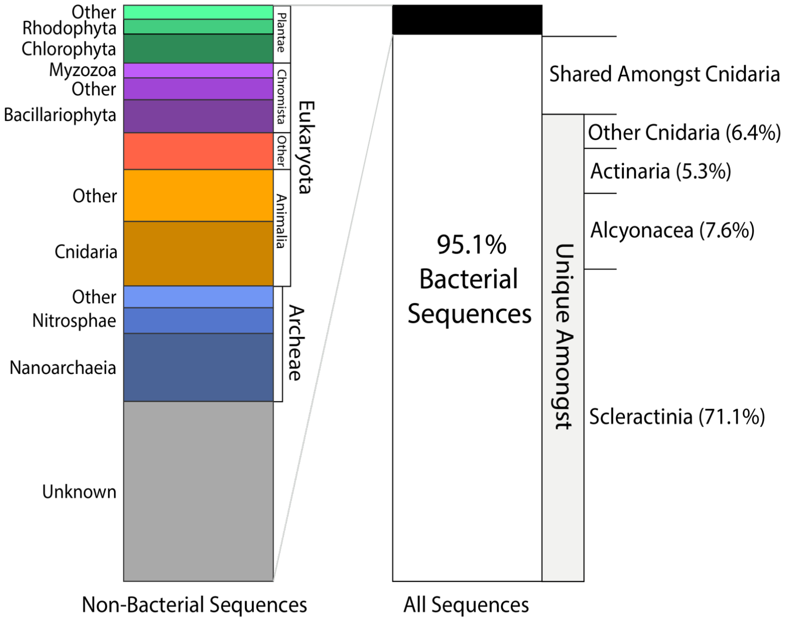

Supplementary Figure 13. The relative abundance of bacterial, archaeal, eukaryote, and unknown sequences identified across all cnidarian libraries in this dataset, as well as presence within cnidarian orders.

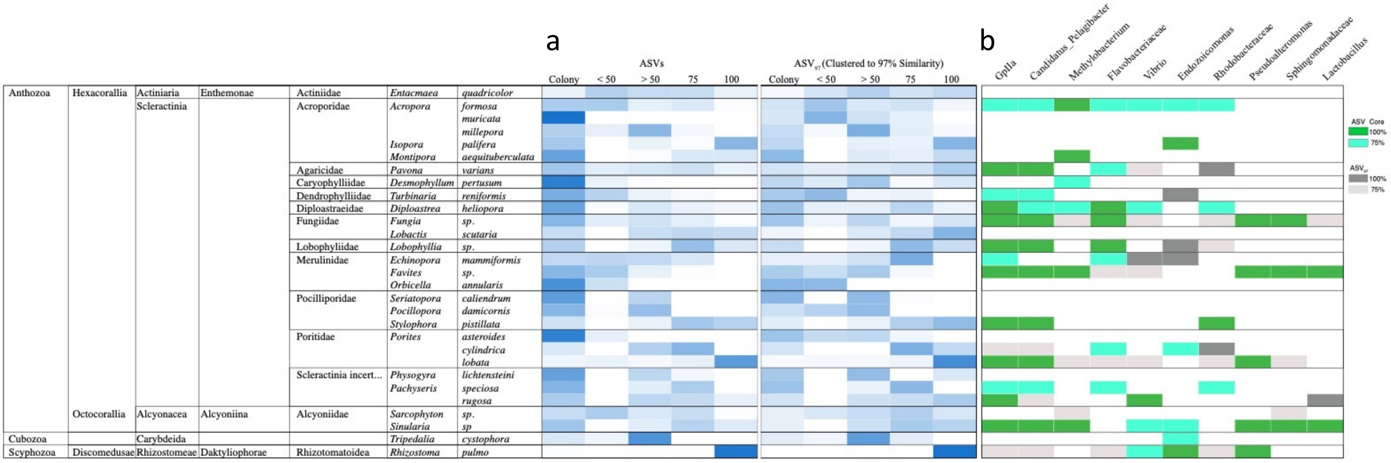

Supplementary Figure 14. The relative abundance of archaeal and bacterial sequences identified within mucus samples per site, averaged to cnidarian family. The number of species present within each family is included in parenthesis. Columns represent the relative abundance of Amplicon Sequence Variants (ASVs) present within fewer than 50% (<50), more than 50% (>50), at least 75% (75), or present in all (100) individual cnidarians (Colony) sampled per site. b, The identity of the 10 most commonly present bacteria in 75% and 100% of individuals sampled per site, as an ASV or an ASV_{97} (clustered at 97% similarity). Color indicates if they were identified as a core member in at least one site per species. Cnidarian species were only included if there was a minimum of four healthy individuals per site.

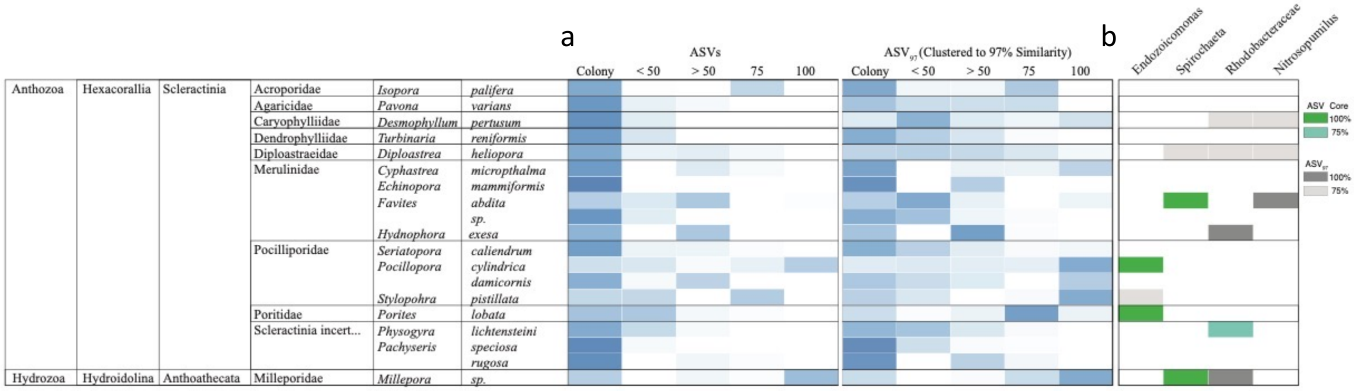

Supplementary Figure 15. The relative abundance of archaeal and bacterial sequences identified within skeletal samples per site, averaged to cnidarian family. Columns represent the relative abundance of Amplicon Sequence Variants (ASVs) present within fewer than 50% (<50), more than 50% (>50), at least 75% (75), or present in all (100) individual cnidarians (Colony) sampled per site. b, The identity of the four most commonly present bacteria in 75% and 100% of individuals sampled per site, as an ASV or an ASV_{97} (clustered at 97% similarity). Color indicates if they were identified as a core member in at least one site per species. Cnidarian species were only included if there was a minimum of four healthy individuals per site.

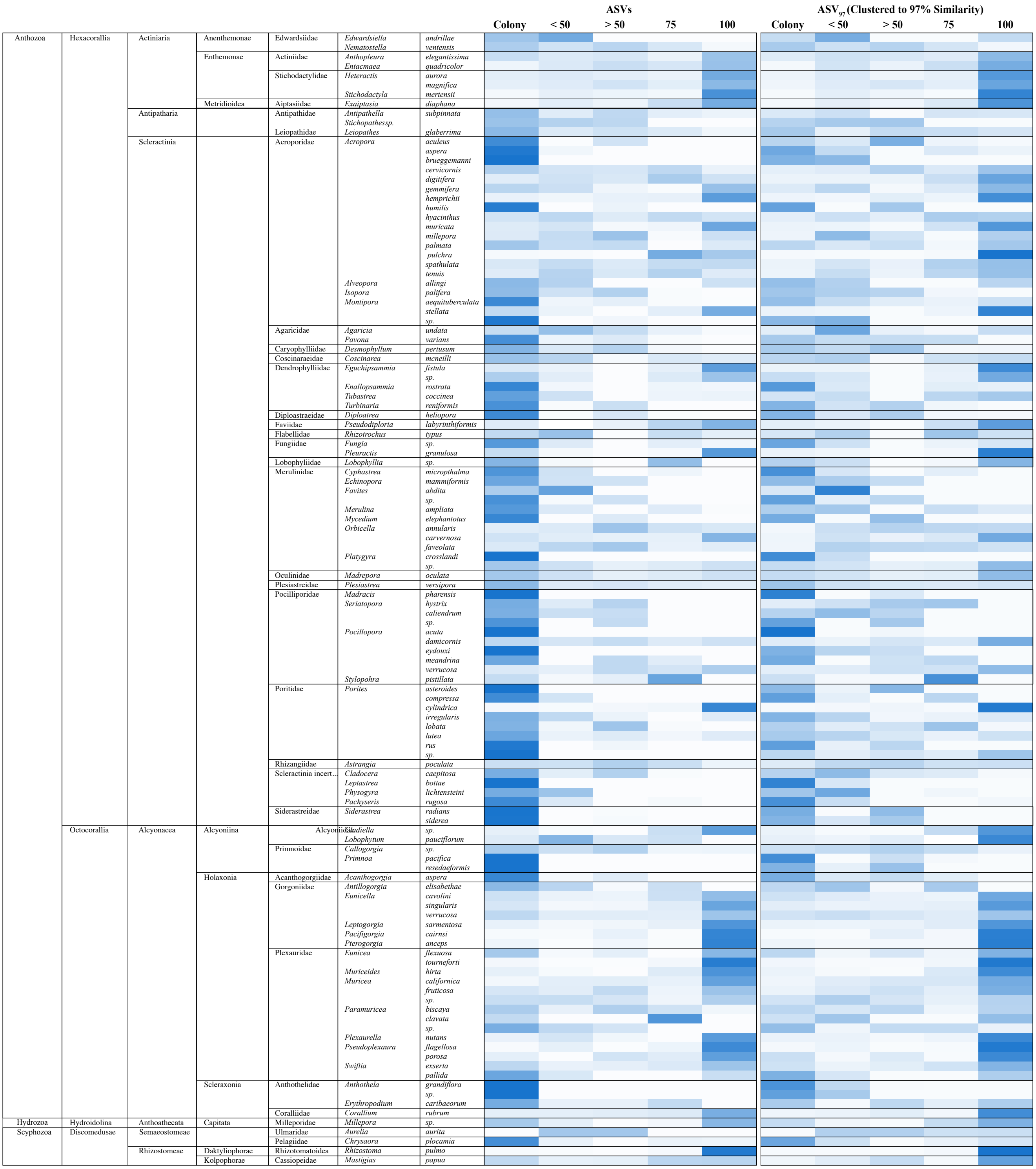

Supplementary Figure 16. The relative abundance of archaeal and bacterial sequences identified within tissue samples per site, averaged to cnidarian family. Columns represent the relative abundance of Amplicon Sequence Variants (ASVs) present within fewer than 50% (<50), more than 50% (>50), at least 75% (75), or present in all (100) individual cnidarians (Colony) sampled per site.

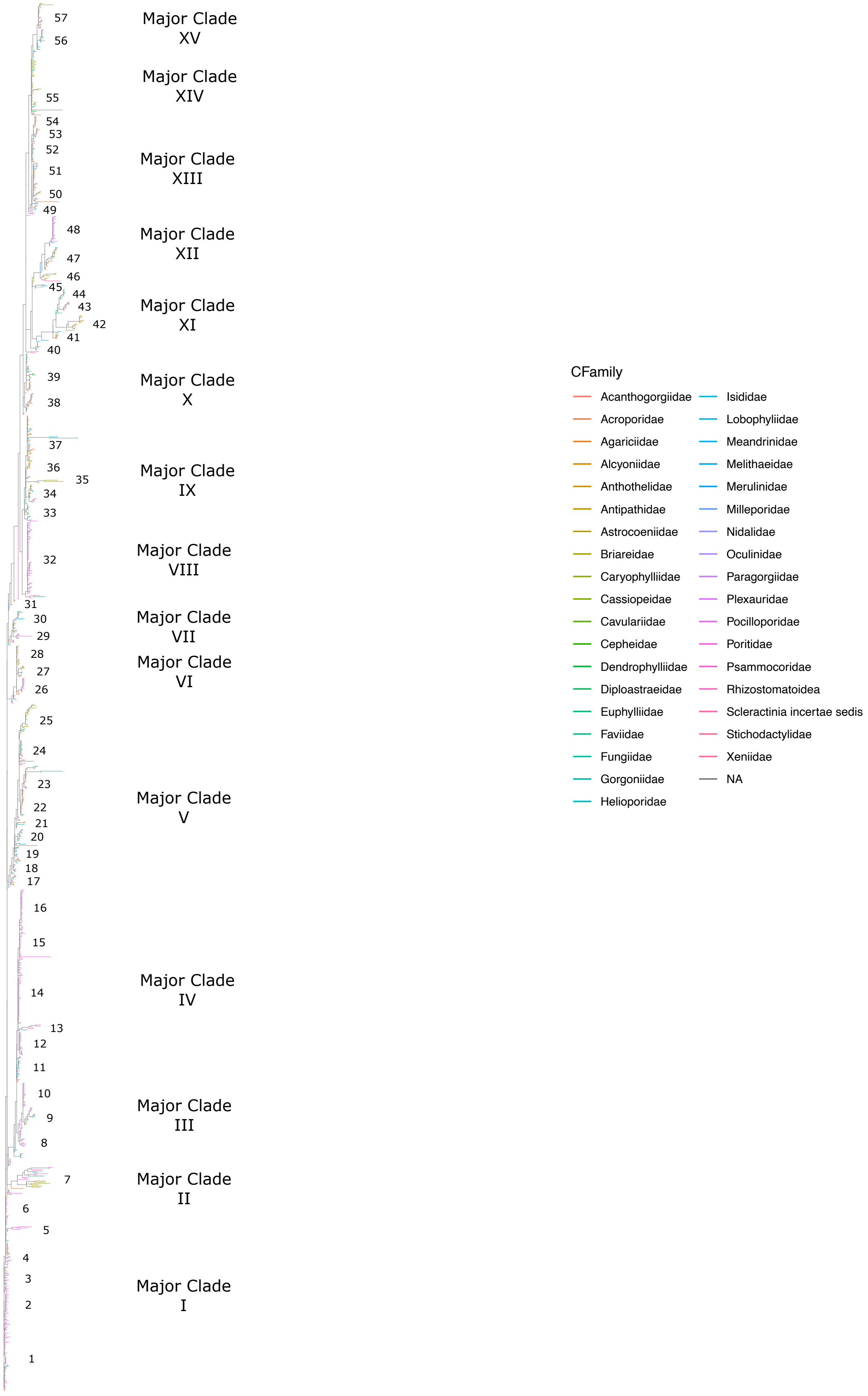

Supplementary Figure 17. Maximum likelihood phylogeny based on the 16S rRNA sequences of *Endozoicomonas* ASVs identified within host families (Illumina Mi-Seq V4 region library). Branch length represents nucleotide variation.

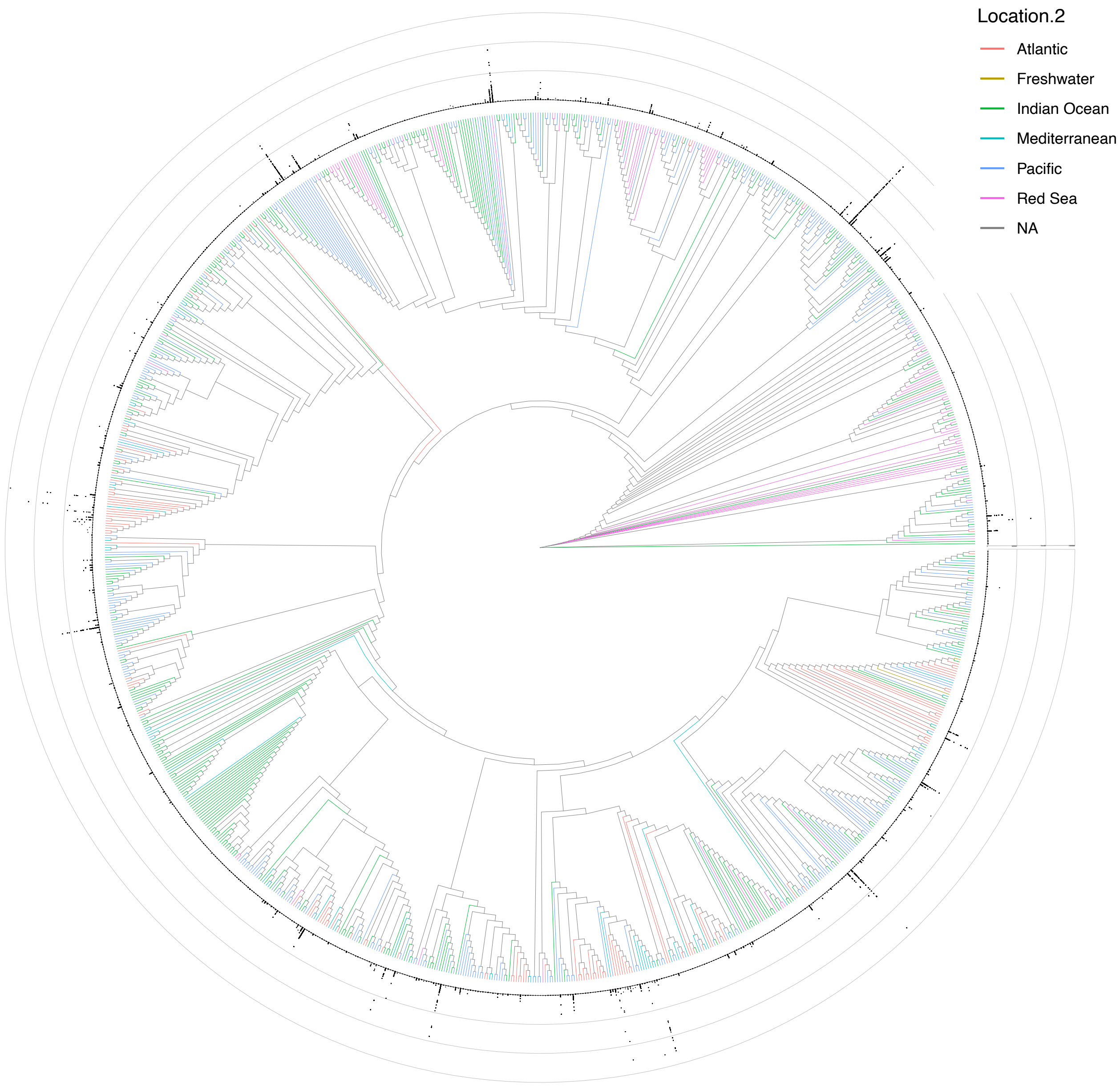

Supplementary Figure 18. ASVs identified within and shared between cnidarian orders (V4 library), with branch lengths colored by water body. Absolute abundance of *Endozoicomonas* ASVs identified per sample, and the number of samples they were identified in are represented by small red circles on the outside of the tree. Branch lengths are standardized for visual purposes only.

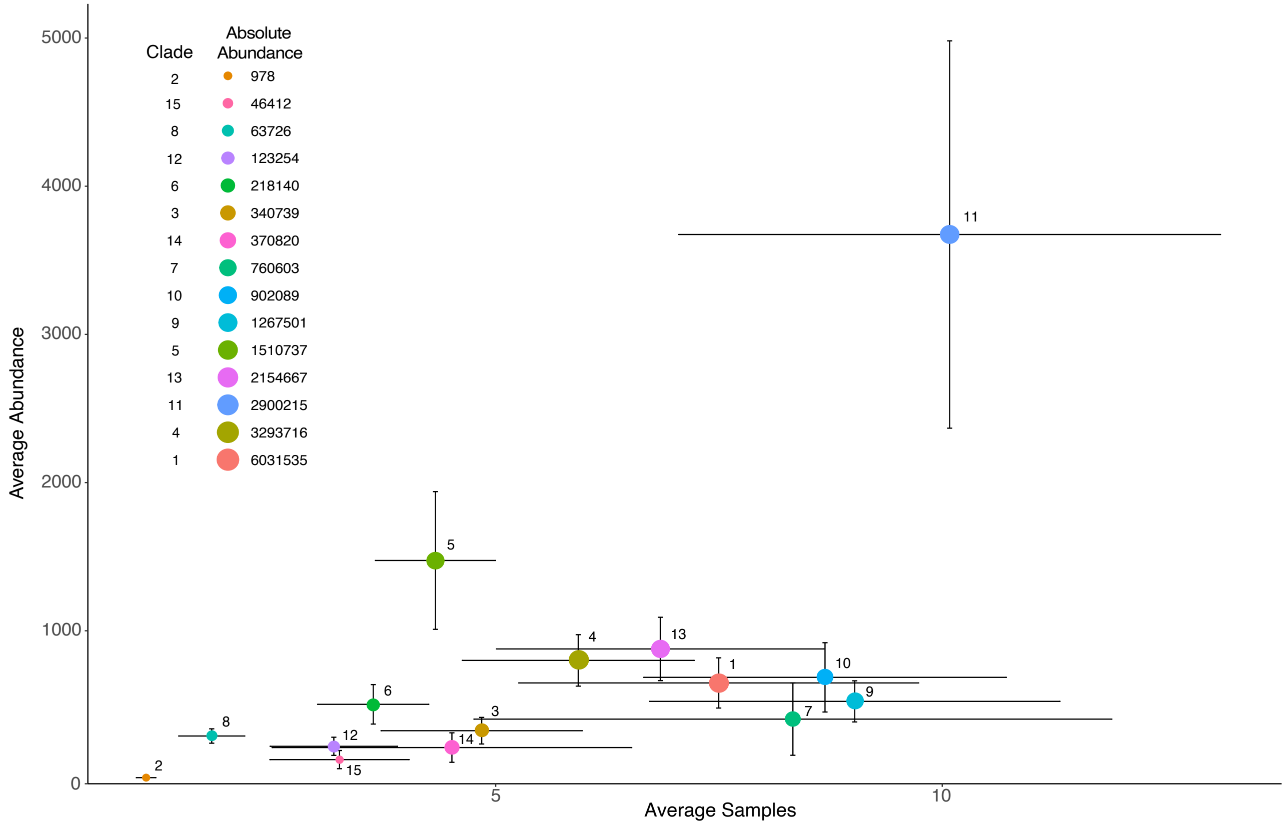

Supplementary Figure 19. Average sequence read counts, and the average number of cnidarian samples that each of the major *Endozoicomonas* ASV clades were identified in (Illumina Mi-Seq V4 library, n=1,634 samples). Error bars represent standard error. Absolute abundance of ASVs within each clade is represented by the diameter of the circle.

PRISMA 2020 flow diagram for new systematic reviews which included searches of databases, registers and other sources

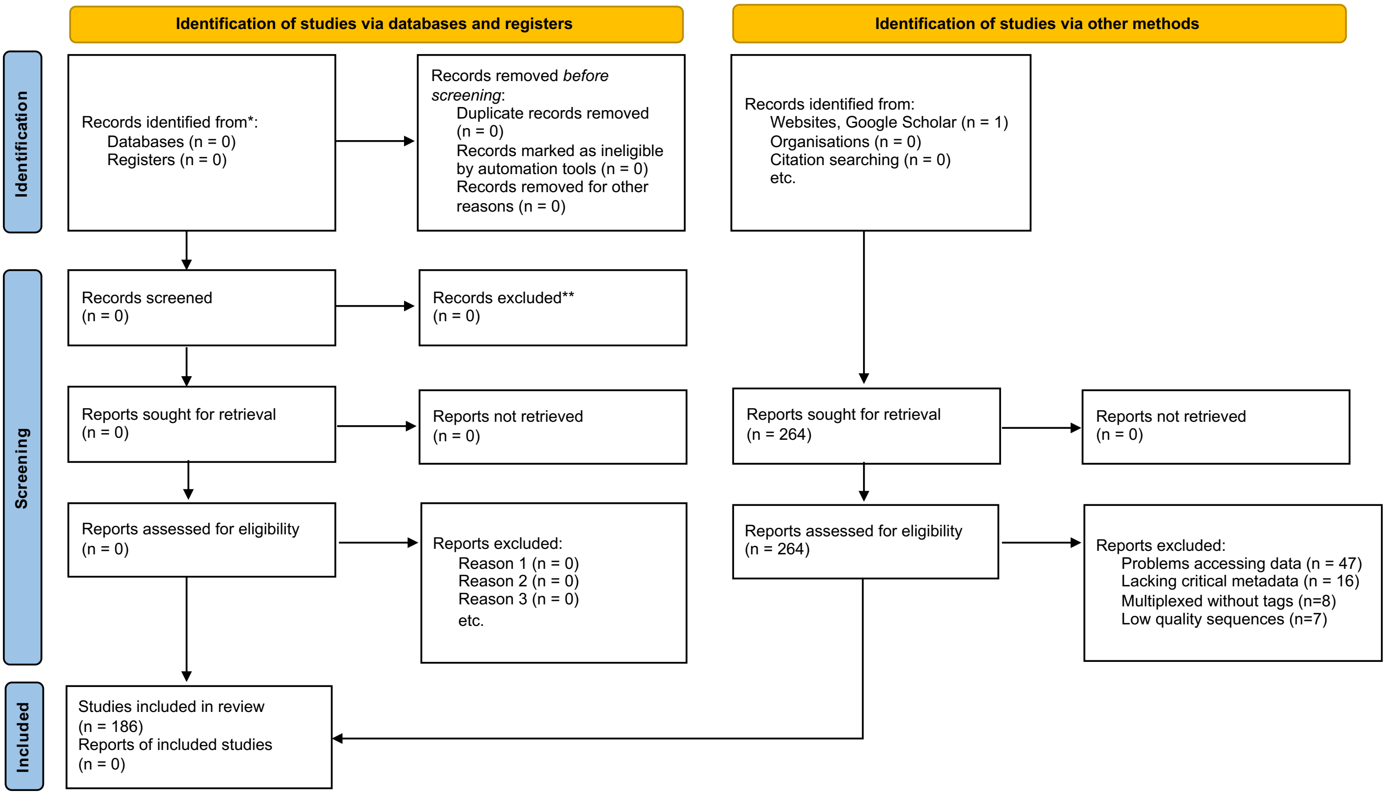

*Consider, if feasible to do so, reporting the number of records identified from each database or register searched (rather than the total number across all databases/registers).
**If automation tools were used, indicate how many records were excluded by a human and how many were excluded by automation tools.

From: Page MJ, McKenzie JE, Bossuyt PM, Boutron I, Hoffmann TC, Mulrow CD, et al. The PRISMA 2020 statement: an updated guideline for reporting systematic reviews. BMJ 2021;372:n71. doi: 10.1136/bmj.n71. For more information, visit: http://www.prisma-statement.org/

Supplementary Figure 20. PRISMA flow diagram for this systematic review.

Supplementary Table 1. Analysis of the effect of different variables on the structure (Bray-Curtis, Jaccard) and diversity (Observed, Shannon, Inverse-Simpson) on rarified (5000 reads) samples (V4_MiSeq_healthy__rarified libraries) using a PERMANOVA (999 permutations) with the original study treated as a random blocking effect. These include the analysis of all cnidarian samples simultaneously (all), all non-scleractinian cnidarians (non-scleractinia), and solely scleractinians (Scleractinia). Emboldened p-values represent those significant after adjusting for false discovery rate.

		Bray-Curtis			Jaccard			ASVs Observed			Shannon			Inverse Simpson		
	df	R^2	*F*	*p*	R^2	*F*	*p*	R^2	*t*	*p*	R^2	*t*	*p*	R^2	*t*	*p*
Cnidaria (all)																
Class	2	<0.01	9.6	**<0.001**	0.05	6.6	**<0.001**	<0.01	6.8	**<0.001**	<0.01	14.9	**<0.001**	<0.01	6.6	**<0.001**
Order	5	0.02	11.6	**<0.001**	0.01	8.2	**<0.001**	0.07	1.3	0.19	0.1	3.9	**<0.001**	0.04	1.4	0.2
Family	28	0.1	10.9	**<0.001**	0.08	7.8	**<0.001**	0.2	2.8	**0.004**	0.03	0.9	0.4	0.2	1.9	0.05
Genus	44	0.08	5.7	**<0.001**	0.07	4.2	**<0.001**	0.02	3.4	**<0.001**	0.3	7.2	**<0.001**	0.2	2.7	**0.006**
Tissue Type	6	0.02	8.5	**<0.001**	0.01	5.6	**<0.001**	0.02	1.2	0.02	0.01	2.5	0.01	0.01	0.5	5.9
Lifestage	4	0.02	12.2	**<0.001**	0.03	7.6	**<0.001**	<0.01	6.8	**<0.001**	0.01	15.2	**<0.001**	<0.01	6.4	**<0.001**
Collection Depth	4	0.02	11.8	**<0.001**	0.01	6.6	**<0.001**	0.1	4.5	**<0.001**	0.2	3.2	**0.002**	0.06	1.7	0.09
Collection Month	11	0.04	10.8	**<0.001**	0.01	6.3	**<0.001**	0.06	5.3	**<0.001**	0.1	11.3	**<0.001**	0.05	4.7	**<0.001**
Symbiodiniaceae?	2	<0.01	2.6	**0.004**	0.01	2.4	**0.004**	0.08	2.4	0.02	0.2	4.5	**<0.001**	0.05	1.2	0.2
WaterBody	21	0.06	8.9	**<0.001**	0.04	5.6	**<0.001**	0.03	1.6	0.1	0.3	2.9	**0.008**	0.2	1.7	0.09
Extraction Kit	6	0.02	8.2	**<0.001**	0.01	5.2	**<0.001**	<0.01	1.7	0.1	0.1	3.9	**<0.001**	0.07	1.2	0.2
Cnidaria (Non_scleractinia)																
Class	2	0.04	15.4	**<0.001**	0.03	9.5	**<0.001**	0.03	6.1	**<0.001**	0.02	7.9	**<0.001**	0.02	4.5	**<0.001**
Order	4	0.05	9.4	**<0.001**	0.03	5.8	**<0.001**	0.1	2.3	0.02	0.2	2.9	**0.004**	0.1	3.6	**<0.001**
Family	11	0.19	16.2	**<0.001**	0.18	10.8	**<0.001**	0.4	0.5	0.6	0.3	1.6	0.1	0.4	0.9	0.4
Genus	11	0.17	12.9	**<0.001**	0.15	9.1	**<0.001**	0.5	0.5	0.6	0.3	1.4	0.2	0.3	0.5	0.6
Tissue Type	5	0.03	4.2	**<0.001**	0.02	3.1	**<0.001**	0.01	1.1	0.3	0.01	2.3	0.02	0.02	0.8	0.4
Lifestage	1	<0.01	5.5	**<0.001**	<0.01	4.2	**<0.001**	<0.01	6.5	**<0.001**	<0.01	8.9	**<0.001**	<0.01	5.1	**<0.001**
Collection Depth	2	0.01	4.5	**<0.001**	0.01	3.5	**<0.001**	0.07	3.3	**0.003**	0.1	5.3	**<0.001**	0.06	4.6	**<0.001**
Collection Month	8	0.03	4.1	**<0.001**	0.03	3.3	**<0.001**	0.13	0.8	0.4	0.09	2.3	0.02	0.1	0.4	0.7
Symbiodiniaceae?	2	0.02	3.8	**<0.001**	0.02	3.4	**<0.001**	<0.01	3.9	**0.001**	<0.01	5.3	**<0.001**	0.08	2.5	0.02
WaterBody	7	0.04	4.2	**<0.001**	0.06	3.9	**<0.001**	0.2	3.2	**<0.001**	0.3	4.9	**0.002**	0.2	4.8	**<0.001**
Extraction Kit	6	<0.01	4.1	**<0.001**	<0.01	2.8	**<0.001**	0.05	1.5	0.2	0.1	2.5	0.05	0.02	0.9	0.4
Cnidaria (Scleractinia)																
Family	17	0.08	10.05	**<0.001**	0.06	6.9	**<0.001**	0.12	5.3	**<0.001**	0.2	12.1	**<0.001**	0.09	3.9	**<0.001**
Genus	33	0.06	4.3	**<0.001**	0.05	3.1	**<0.001**	0.2	3.4	**<0.001**	0.2	7.4	**<0.001**	0.1	2.5	0.01
Tissue Type	3	0.02	13.3	**<0.001**	0.01	8.23	**<0.001**	0.01	4.5	**<0.001**	0.01	11.6	**<0.001**	<0.01	3.9	**<0.001**
Lifestage	4	0.02	11.6	**<0.001**	0.02	7.6	**<0.001**	<0.01	5.8	**<0.001**	0.02	12.9	**<0.001**	<0.01	5.5	**<0.001**
Collection Depth	2	0.02	19.2	**<0.001**	0.01	13.6	**<0.001**	0.12	1.5	0.12	0.2	0.4	0.7	0.04	2.1	0.04
Collection Month	11	0.04	8.8	**<0.001**	0.03	7.4	**<0.001**	0.05	4.6	**<0.001**	0.07	10.7	**<0.001**	0.04	4.4	**<0.001**
Symbiodiniaceae?	2	<0.01	2.4	**<0.001**	<0.01	1.9	**0.002**	0.04	2.4	0.02	0.08	4.4	**<0.001**	0.02	1.1	0.2
WaterBody	18	0.08	9.03	**<0.001**	0.06	6.5	**<0.001**	0.4	5.6	**<0.001**	0.3	6.5	**<0.001**	0.2	4.4	**<0.001**
Extraction Kit	5	0.02	8.9	**<0.001**	0.02	6.3	**<0.001**	0.02	1.7	0.1	0.2	4.1	**<0.001**	0.1	1.3	0.2

Supplementary Table 2. Analysis of the effect of different variables on the structure (Bray-Curtis, Jaccard) and diversity (Observed, Shannon, Inverse-Simpson) on unrarified samples (V4_MiSeq_healthy__unrarified libraries) using a PERMANOVA (999 permutations) with the original study treated as a random blocking effect. These include the analysis of all cnidarian samples simultaneously (all), all non-scleractinian cnidarians (non-scleractinia), and solely scleractinians (Scleractinia). Emboldened p-values represent those significant after adjusting for false discovery rate.

		Bray-Curtis			Jaccard			ASVs Observed			Shannon			Inverse Simpson		
	df	R^2	*F*	*p*	R^2	*F*	*p*	R^2	*t*	*p*	R^2	*t*	*p*	R^2	*t*	*p*
Cnidaria (all)																
Class	2	<0.01	7.7	**<0.001**	0.003	5.1	**<0.001**	<0.01	5.5	**<0.001**	<0.01	11.7	**<0.001**	<0.01	6.1	**<0.001**
Order	5	0.02	11.04	**<0.001**	0.01	7.3	**<0.001**	0.05	1.2	0.3	0.08	3.7	**<0.001**	0.04	1.14	0.3
Family	31	0.08	8.8	**<0.001**	0.06	6.1	**<0.001**	0.2	2.9	**0.004**	<0.01	0.4	0.7	0.1	2.06	0.04
Genus	51	0.08	4.9	**<0.001**	0.06	3.4	**<0.001**	0.2	2.7	**0.008**	0.2	6.5	**<0.001**	0.1	2.5	**0.001**
Tissue Type	6	0.01	7.3	**<0.001**	0.01	4.7	**<0.001**	0.01	0.9	0.4	0.02	2.05	0.04	0.01	0.5	0.6
Lifestage	6	0.02	11.3	**<0.001**	0.03	6.5	**<0.001**	<0.01	5.6	**<0.001**	0.03	12.8	**<0.001**	<0.01	6.1	**<0.001**
Collection Depth	4	0.01	8.5	**<0.001**	0.01	6.2	**<0.001**	0.07	0.8	0.4	0.1	2.9	**0.007**	0.05	1.6	0.1
Collection Month	11	0.05	4.6	**<0.001**	<0.01	4.4	**<0.001**	0.06	4.5	**<0.001**	0.09	10.1	**<0.001**	0.05	4.7	**<0.001**
Symbiodiniaceae?	3	<0.01	2.07	**0.004**	<0.01	1.9	**<0.001**	0.04	1.9	0.05	0.1	4.4	**<0.001**	0.04	1.2	0.2
WaterBody	24	0.05	6.4	**<0.001**	0.03	4.2	**<0.001**	0.4	1.2	0.2	0.25	2.8	**<0.001**	0.2	1.8	0.08
Extraction Kit	6	0.02	8.3	**<0.001**	0.01	5.2	**<0.001**	0.02	1.3	0.2	0.1	2.7	**0.008**	0.09	1.2	0.2
Cnidaria (Non_scleractinia)																
Class	2	0.03	11.7	**<0.001**	0.18	7.1	**<0.001**	0.02	5.7	**<0.001**	0.02	7.4	**<0.001**	0.02	4.5	**<0.001**
Order	4	0.04	8.2	**<0.001**	0.25	4.9	**<0.001**	0.04	1.7	0.09	0.2	2.9	**0.004**	0.09	2.4	0.02
Family	12	0.2	14.2	**<0.001**	0.14	9.1	**<0.001**	0.05	1.2	0.7	0.3	1.5	0.1	0.04	1.8	0.5
Genus	11	0.2	13.4	**<0.001**	0.13	9.2	**<0.001**	0.05	1.5	0.6	0.3	1.4	0.2	0.03	4.7	0.6
Tissue Type	5	0.02	3.3	**<0.001**	0.02	2.5	**<0.001**	<0.01	1	0.3	<0.01	2.1	0.04	0.01	1.8	0.5
Lifestage	1	<0.01	4.3	**<0.001**	<0.01	3.3	**<0.001**	0.2	5.9	**<0.001**	<0.01	8.3	**<0.001**	<0.01	5.1	**<0.001**
Collection Depth	2	<0.01	3.7	**<0.001**	<0.01	2.6	**<0.001**	0.04	2.1	0.04	0.2	5.09	**<0.001**	0.1	4.7	**<0.001**
Collection Month	8	0.02	4.3	**<0.001**	0.29	2.7	**<0.001**	0.1	0.8	0.4	0.09	2.4	**0.008**	0.1	0.6	0
Symbiodiniaceae?	2	0.01	3.4	**<0.001**	0.01	4.1	**<0.001**	<0.01	3.6	**0.002**	<0.01	5.07	**<0.001**	<0.01	2.6	0.5
WaterBody	8	0.03	3.7	**<0.001**	0.03	6.4	**<0.001**	0.2	1.9	0.1	0.3	4.2	**0.007**	0.2	5.2	**<0.001**
Extraction Kit	7	<0.01	3.5	**<0.001**	0.02	2.5	**<0.001**	0.07	1.1	0.9	0.1	1.1	0.7	0.02	1.3	0.9
Cnidaria (Scleractinia)																
Family	19	0.06	7.2	**<0.001**	0.04	4.8	**<0.001**	0.07	4.8	**<0.001**	0.1	9.9	**<0.001**	0.08	3.9	**<0.001**
Genus	40	0.05	3.4	**<0.001**	0.04	2.4	**<0.001**	0.1	2.5	0.01	0.2	6.7	**<0.001**	0.1	2.4	0.02
Tissue Type	3	0.01	11.5	**<0.001**	<0.01	6.9	**<0.001**	<0.01	3.8	**<0.001**	0.02	9.3	**<0.001**	<0.01	3.9	**<0.001**
Lifestage	6	0.03	10.8	**<0.001**	0.02	7.3	**<0.001**	<0.01	4.9	**<0.001**	0.04	10.5	**<0.001**	<0.01	5.2	**<0.001**
Collection Depth	4	0.01	7.5	**<0.001**	<0.01	5.3	**0.005**	0.08	1.2	0.2	0.1	0.7	0.5	0.04	2.3	0.02
Collection Month	11	0.03	7.5	**<0.001**	<0.01	4.7	**<0.001**	0.04	4.2	**<0.001**	0.07	8.9	**<0.001**	0.05	3.9	**<0.001**
Symbiodiniaceae?	3	<0.01	1.9	**<0.001**	0.04	1.6	**<0.001**	0.03	1.9	0.05	0.04	4.1	**<0.001**	0.02	1.2	0.2
WaterBody	19	0.05	6.9	**<0.001**	0.01	4.8	**<0.001**	0.43	6.7	**<0.001**	0.3	5.8	**<0.001**	0.2	4.9	**<0.001**
Extraction Kit	5	0.02	8.9	**<0.001**	0.01	6.2	**<0.001**	0.1	1.6	0.1	0.2	3.6	**0.001**	0.1	1.3	0.2

Supplementary Table 3. Analysis of the Bray-Curtis dispersion (distance from centroid) for all cnidarian orders (V4 library). These include the analysis of all rarefied data (minimum 5000 reads), and unrarefied data.

**Cnidaria (rarified)**								
	Actiniaria	Alcyonacea	Anthoathecata	Antipatharia	Helioporacea	Rhizostomeae	Scleractinia	Zoantharia
Actiniaria		2.00E-03	9.98E-01	9.99E-04	5.05E-01	1.70E-02	9.99E-04	0.4675
Alcyonacea	3.03E-06		9.99E-04	1.70E-02	5.00E-03	2.00E-03	9.99E-04	0.016
Anthoathecata	9.96E-01	8.94E-07		9.99E-04	4.27E-01	1.30E-02	9.99E-04	0.4366
Antipatharia	2.83E-04	2.07E-02	1.08E-04		4.00E-03	1.39E-01	9.99E-04	0.015
Helioporacea	4.72E-01	8.09E-06	4.32E-01	4.74E-06		2.10E-02	9.99E-04	0.1139
Rhizostomeae	1.41E-02	1.56E-04	9.19E-03	1.32E-01	1.74E-03		9.99E-04	0.2208
Scleractinia	4.69E-165	6.29E-43	6.55E-183	6.26E-143	2.16E-169	8.98E-167		0.001
Zoantharia	4.01E-01	2.56E-03	3.66E-01	8.48E-03	1.22E-01	2.32E-01	1.49E-121	
**Cnidaria (unrarified)**								
Actiniaria		9.99E-04	4.28E-01	2.70E-02	1.06E-01	1.20E-01	9.99E-04	0.8042
Alcyonacea	1.40E-10		9.99E-04	9.99E-04	9.99E-04	9.99E-04	9.99E-04	0.001
Anthoathecata	3.77E-01	3.55E-15		2.00E-03	2.23E-01	5.00E-03	9.99E-04	0.1698
Antipatharia	2.13E-02	1.05E-05	1.88E-05		2.00E-03	3.10E-01	9.99E-04	0.032
Helioporacea	1.16E-01	6.19E-15	2.61E-01	1.35E-06		5.00E-03	9.99E-04	0.05
Rhizostomeae	1.17E-01	1.04E-08	2.20E-03	3.01E-01	2.19E-04		9.99E-04	0.2498
Scleractinia	5.28E-162	2.49E-82	2.99E-271	3.15E-199	1.84E-249	5.51E-201		0.001
Zoantharia	7.76E-01	4.41E-06	1.85E-01	3.07E-02	5.46E-02	2.72E-01	7.74E-151	

Supplementary Table 4. Analysis of the Bray-Curtis dispersion (distance from centroid) for all non-scleractinian orders (V4 library). These include the analysis of all rarefied data (minimum 5000 reads), and unrarefied data.

**Cnidaria_nonscleractinia (rarified)**							
	Actiniaria	Alcyonacea	Anthoathecata	Antipatharia	Helioporacea	Rhizostomeae	Zoantharia
Actiniaria		9.99E-04	3.99E-01	2.40E-02	1.19E-01	1.42E-01	0.7712
Alcyonacea	1.38E-10		9.99E-04	9.99E-04	9.99E-04	9.99E-04	0.001
Anthoathecata	3.78E-01	3.72E-15		9.99E-04	2.80E-01	5.00E-03	0.1798
Antipatharia	2.13E-02	1.04E-05	1.98E-05		9.99E-04	3.10E-01	0.032
Helioporacea	1.16E-01	6.04E-15	2.62E-01	1.35E-06		9.99E-04	0.0519
Rhizostomeae	1.17E-01	1.06E-08	2.33E-03	3.02E-01	2.29E-04		0.2677
Zoantharia	7.76E-01	4.38E-06	1.87E-01	3.08E-02	5.46E-02	2.74E-01	
**Cnidaria_nonscleractinia (unrarified)**							
Actiniaria		9.99E-04	9.72E-01	9.99E-04	5.20E-01	1.60E-02	0.3766
Alcyonacea	2.62E-06		9.99E-04	3.50E-02	9.99E-04	9.99E-04	0.003
Anthoathecata	9.61E-01	1.37E-06		9.99E-04	4.69E-01	1.90E-02	0.3916
Antipatharia	2.42E-04	2.28E-02	1.61E-04		9.99E-04	1.34E-01	0.009
Helioporacea	4.87E-01	9.52E-06	4.37E-01	5.11E-06		6.99E-03	0.1199
Rhizostomeae	1.37E-02	1.56E-04	1.21E-02	1.25E-01	2.03E-03		0.2218
Zoantharia	3.96E-01	2.51E-03	3.95E-01	7.81E-03	1.27E-01	2.34E-01	

Supplementary Table 5. Analysis of the Jaccard dispersion (distance from centroid) for all cnidarian orders (V4 library). These include the analysis of all rarefied data (minimum 5000 reads), and unrarefied data.

**Cnidaria (rarified)**								
	Actiniaria	Alcyonacea	Anthoathecata	Antipatharia	Helioporacea	Rhizostomeae	Scleractinia	Zoantharia
Actiniaria		1.99E-03	9.82E-01	9.99E-04	2.23E-01	2.39E-02	9.99E-04	0.86
Alcyonacea	4.31E-05		1.99E-03	2.67E-01	1.99E-03	2.99E-03	9.99E-04	0.01
Anthoathecata	9.82E-01	2.26E-05		9.99E-04	2.03E-01	2.19E-02	9.99E-04	0.89
Antipatharia	3.09E-06	2.51E-01	5.75E-06		9.99E-04	8.99E-03	9.99E-04	0.01
Helioporacea	2.12E-01	4.80E-06	2.15E-01	1.05E-09		7.99E-03	9.99E-04	0.23
Rhizostomeae	1.61E-02	7.86E-04	1.59E-02	1.59E-02	3.61E-04		9.99E-04	0.06
Scleractinia	1.37E-187	1.17E-44	1.50E-188	2.59E-118	5.57E-217	6.82E-169		0.01
Zoantharia	3.38E-01	3.38E-04	8.30E-01	2.14E-04	2.18E-01	6.29E-02	1.04E-143	
**Cnidaria (unrarified)**								
Actiniaria		9.99E-04	4.02E-01	1.79E-02	1.18E-01	1.12E-01	9.99E-04	0.82
Alcyonacea	1.40E-10		9.99E-04	9.99E-04	9.99E-04	9.99E-04	9.99E-04	0.01
Anthoathecata	3.77E-01	3.55E-15		2.99E-03	2.43E-01	8.99E-03	9.99E-04	0.19
Antipatharia	2.13E-02	1.05E-05	1.88E-05		2.99E-03	3.23E-01	9.99E-04	0.03
Helioporacea	1.16E-01	6.18E-15	2.61E-01	1.35E-06		8.99E-03	9.99E-04	0.06
Rhizostomeae	1.16E-01	1.04E-08	2.20E-03	3.01E-01	2.19E-04		9.99E-04	0.27
Scleractinia	5.28E-162	2.48E-82	2.99E-271	3.15E-199	1.84E-249	5.51E-201		0.01
Zoantharia	7.76E-01	4.41E-06	1.84E-01	3.07E-02	5.45E-02	2.72E-01	7.74E-151	

Supplementary Table 6. Analysis of the Jaccard dispersion (distance from centroid) for all non-scleractinian orders (V4 library). These include the analysis of all rarefied data (minimum 5000 reads), and unrarefied data.

**Cnidaria_nonscleractinia (rarified)**							
	Actiniaria	Alcyonacea	Anthoathecata	Antipatharia	Helioporacea	Rhizostomeae	Zoantharia
Actiniaria		9.99E-04	9.63E-01	9.99E-04	2.44E-01	1.09E-02	0.82
Alcyonacea	3.89E-05		9.99E-04	2.82E-01	9.99E-04	2.99E-03	0.01
Anthoathecata	9.58E-01	2.68E-05		9.99E-04	2.37E-01	2.59E-02	0.87
Antipatharia	2.64E-06	2.62E-01	8.56E-06		9.99E-04	1.79E-02	0.01
Helioporacea	2.36E-01	5.38E-06	2.23E-01	1.07E-09		2.99E-03	0.22
Rhizostomeae	1.51E-02	7.94E-04	1.79E-02	1.48E-02	3.77E-04		0.06
Zoantharia	8.13E-01	3.26E-04	8.49E-01	2.14E-14	2.27E-01	6.18E-02	
**Cnidaria_nonscleractinia (unrarified)**							
Actiniaria		9.99E-04	2.53E-01	6.99E-03	2.19E-02	1.56E-01	0.75
Alcyonacea	3.26E-08		9.99E-04	3.99E-03	9.99E-04	9.99E-04	0.01
Anthoathecata	2.43E-01	4.93E-12		9.99E-04	7.69E-02	3.99E-03	0.45
Antipatharia	3.17E-03	4.69E-03	1.81E-07		9.99E-04	5.99E-02	0.01
Helioporacea	1.72E-02	9.11E-16	8.47E-02	2.06E-10		9.99E-04	0.05
Rhizostomeae	1.52E-01	3.91E-07	2.69E-03	6.63E-02	1.51E-05		0.09
Zoantharia	7.44E-01	4.17E-07	4.19E-01	2.88E-04	4.14E-02	8.03E-02	
