# Supplementary material for: Systematic review of cnidarian microbiomes reveals insights into the structure, specificity, and fidelity of marine associations
Source: Nat Commun. 2023 Aug 14;14:4899. doi: 10.1038/s41467-023-39876-6 (PMC10425419; doi:10.1038/s41467-023-39876-6)
Supplement: Supplementary file 3 — Description of Additional Supplementary Files [file 41467_2023_39876_MOESM3_ESM.pdf]

Description of Additional Supplementary Files

File Name: Supplementary Data 1

Description: Metadata for the 16,012 microbial samples included in this dataset. Metadata was collated from the originally published studies, available supplementary information, and from online databases.
