# Supplementary material for: Systematic review of cnidarian microbiomes reveals insights into the structure, specificity, and fidelity of marine associations
Source: Nat Commun. 2023 Aug 14;14:4899. doi: 10.1038/s41467-023-39876-6 (PMC10425419; doi:10.1038/s41467-023-39876-6)
Supplement: Supplementary file 2 — Peer Review File [file 41467_2023_39876_MOESM2_ESM.pdf]

nature portfolio

Peer Review File

Systematic review of cnidarian microbiomes reveals insights into the structure, specificity, and fidelity of marine associations

Open Access This file is licensed under a Creative Commons Attribution 4.0 International License, which permits use, sharing, adaptation, distribution and reproduction in any medium or format, as long as you give appropriate credit to the original author(s) and the source, provide a link to the Creative Commons license, and indicate if changes were made. In the cases where the authors are anonymous, such as is the case for the reports of anonymous peer reviewers, author attribution should be to 'Anonymous Referee' followed by a clear attribution to the source work. The images or other third party material in this file are included in the article's Creative Commons license, unless indicated otherwise in a credit line to the material. If material is not included in the article's Creative Commons license and your intended use is not permitted by statutory regulation or exceeds the permitted use, you will need to obtain permission directly from the copyright holder. To view a copy of this license, visit http://creativecommons.org/licenses/by/4.0/.

REVIEWER COMMENTS</B>

Reviewer #1 (Remarks to the Author):

Title: Systematic review of cnidarian microbiomes reveals insights into the structure, specificity, and fidelity of marine associations

Authors: McCauley et al.

The authors have made a herculean effort to examine a large volume of 16S rRNA gene amplicon data available from cnidarians and sponges via a bioinformatic meta-analysis with the goal of uncovering trends to better understand the microbial symbioses occurring within these holobionts. These data were derived from relevant studies between 2011–2021 and the metadata curated manually from the publications (since in general, metadata are poorly or inconsistently included with raw DNA sequence submissions to databases). Amassing useful metadata as has been done here is a HUGE amount of work and is why these types of reviews are rare.

Lines 69-75 correctly brings up the issue of how different protocols (e.g., preservation, extraction, amplification primers, sequencing platform, bioinformatic pipeline, combinations thereof) complicate or confound cross-study comparisons. Of the multiple versions of datasets examined in this manuscript, the largest (which I'll call #1 = 'maximum cnidarians') includes not only different sequencing platforms (e.g., Roche 454, Illumina MiSeq, HiSeq,), but different primers/variable region targets). However, the bulk of the analyses use slightly smaller datasets (V4, V4_p, subsets thereof) which are restricted to one platform (Illumina MiSeq) and one variable region (V4), limiting two of the major factors (a wise and necessary choice). It should be made a little more explicit in the introduction that this happened and why (e.g., totally valid choice to avoid the difficult-to-quantity amounts of methodological variability introduced by different platforms and variable region primers in order to increase the quality of the results; Tremblay et al. 2015, DOI 10.3389/fmicb.2015.00771).

The Abstract (in order to meet the small word limit) and the Introduction (lines 86-89) only clue the reader into a single massive dataset being analyzed, but in fact the paper analyzes several and the majority of the analyses are NOT conducted on the single massive dataset (for obvious reasons just mentioned above). In addition to mentioning it in the Introduction (and to expedite matching up the specific dataset from which a given result is derived), I highly recommend that the Results section begin with an overview that makes clear what each of the datasets encompasses (it might make sense to create a table listing the relevant numbers). I have uploaded an example table based on the numbers I found in the manuscript to illustrate what I mean since it is not possible to include it here; in it I attempt to number the databases for clarity and use those numbers in the rest of this review.

It would also be helpful to clarify the overlaps between these datasets. As written, I assume that V4_P equals V4 (2b) plus sponge and Symbiodiniaceae culture microbiomes but maybe that is not exactly right? The numbers in a table like the mock up I've provided would help clarify those kinds of things too. Is #4 core microbiome derived from 2b (V4 unrarefied)? It should be noted that the Supplemental Materials DO explicitly describe which underlying dataset was used for each of them (thank you!). I appreciate the choice to group the Results topically, rather than by dataset, but without being able to easily indicate when jumping from one dataset to the next, it makes it hard for the reader to keep track. It might be helpful in the Methods section to describe the flow from the largest dataset down to the various sub-datasets.

The current first section of Results (lines 100-114) reads as if those conclusions are derived from dataset #1 (max cnidarians) but then references Figures 2 and 3 which both say they come from dataset #2 (V4). I suspect that both datasets #1 and #2 support these findings but because of the confounding factors involved in #1 (different variable regions, different sequencing platforms, etc.) it was cleaner to create figures using #2. For transparency's sake, it would be helpful to just explain that (or whatever drove those choices if my guess is wrong). The second section of Results (lines 116-143) says it comes from #2 V4 and mentions unrarefied and rarefied datasets, so 2a and 2b). The third section of Results (lines 145-175) begins with V4 but mentions separating into

scleractinian vs not datasets (which I've named 2.1 and 2.2 in my table). Then lines 153-157 come back to dataset V4 2a/2b. Then we switch to V4_p (line 158), but at the end we come back to 2.1 V4_scleractinian subset. Having now made it through the full Results section, I'm now not sure if some of this might not actually involve subsets of #5? The fourth section of Results (lines 177-212), we start with #1 max cnidarians (line 178) and it appears that the rest of this paragraph and all parts of Figure 4 are based on #1, but then lines 186-188 referencing Fig 4b mentions "ASVs identified in cultured Symbiodiniaceae microbiomes were identified in any cnidarian microbiome" which suggests we have jumped to V4_p. The paragraph beginning with line 203 talks about the scleractinian microbiome so it is unclear if this is based on #1 or 2.1 V4_scleractinian subset. Based on the title, one would guess that the fifth section of Results (lines 214-237) and Figure 5 is based on #4 (core microbiome) but that is not specified? Sixth section of Results, line 242 "examined Endozoicomonas ASVs within all libraries; please specify (e.g., 1, 2 and 3? 1-4? 1-5?). Line 261 specifies V4 (2a or 2b?). Seventh section of Results (lines 276-290) begins in #1 (based on ASV numbers). Are the 11,995 unidentifiable ASVs also from #1 or from 2b?

The results support the major conclusions offered:
• That Cnidarians host an incredible diversity of archaeal and bacterial phyla.
• That host phylogeny and ocean body have the largest influence on microbial diversity
• The diversity and geographic/host-specific associations uncovered for Endozoicomonas

I was also particularly struck by some of the minor discoveries; e.g., that Ostreobium was found not only in scleractinians (where I'm used to seeing it, as colored bands growing in the skeleton) but also in Octocorals and black corals.

Within the conclusion about huge diversity and how the majority of ASVs are incredibly rare <1% prevalence), I think the authors have missed (or chosen to avoid) an opportunity that having a large dataset like this could afford them to answer a vexing question within the coral/cnidarian community. Most 16S rRNA amplicon surveys conducted prior to 2018 (and some after that), did not include kit contamination blanks. This makes it difficult or impossible to be certain which of those rare ASVs are genuine associates and which are contamination from the extraction kit or other laboratory reagents. This was initially brought up by Salter et al. (2014) DOI 10.1186/s12915-014-0087-z and expanded by Glassing et al. (2016) DOI 10.1186/s13099-016-0103-7 and de Goffau et al. (2018) DOI 10.1038/s41564-018-0202-y because it can have such a large impact on interpretation of low biomass samples (which can include cnidarian samples). The authors mention this in passing in lines 379-380. I would argue that the V4 dataset likely includes enough studies that are on both sides of this shift in community quality requirements (the inclusion of kit blank controls) that it may be possible to determine (especially with ASVs over OTUs) whether core microbiota such as Pseudoalteromonas, Alteromonas, and Propionibacterium are real or contaminants (or both but discernable by ASV sequence). This might even be as simple as going into the core microbiome dataset (mercifully smaller than the full V4) and flagging which studies included kit blank controls and which did not. If these bacterial groups (or other genera flagged by Salter and Glassing as common contaminants) only show up in studies that did not include controls, that is strongly suggestive that they are only contaminants. If they show up in studies that DID run controls and spot checks reveal the controls did not contain them, then that would data rescue this information even for older studies that lacked controls. Which would be another major conclusion to offer.

In some cases, sampling depth was found to be a significant factor. I noticed in skimming through Supplemental Table 1 that there are a number of studies for which that information was unknown. It would be helpful to know what percentage of the total studies in whichever dataset(s) found depth to be significant contained this information. E.g., depth information was available for 90/80/70% of the total studies in dataset V4. Large numbers are comforting, while an unknown but non-100% number makes it harder to be convinced by the significance (I want to believe!).

While this study of cnidarian microbiomes has been under review, a systematic revision was published that elevated anthozoan sub-classes Octocorallia and Hexacorallia to the rank of Class, replaced the 3 Octocorallia orders with 2 new ones, and revised most of the family-level structure. I would not expect this to impact any of your major conclusions but you will want to at least

acknowledge this somewhere in the paper, much as you have acknowledged the recent (incredibly annoying) microbial taxonomic changes (e.g., Proteobacteria to Pseudomonadota): McFadden et al. 2022, DOI 10.18061/bssb.v1i3.8735.

The manuscript is appropriately referenced, showing a command of the relevant literature. Numbered in-text citations match the bibliography list and correctly support the statements or methods mentioned. There are a couple of parentheticals (author, date) that should be changed to numbered for consistency:
Line 140: Ying et al. 2018
Line 471: Rstudio reference

Figure 1: The legend explains what the pie charts mean and the inset explains the size of the chart and the order each color in the charts represents. Additional text is required to explain what the small colored circles are and what the color-coding means. Presumably each small circle is a study (since there are not 12,010 circles, so they can't be samples) and the colors appear to align with ocean basins?

A rapid skim uncovered incorrect data in Supplemental Table 1, suggesting that you need to do another round of QC, since the host and environmental data you have compiled is one of the key community benefits of this effort:
Row 4879
SRR9176498, SAMN11849804
Listed in table as being Desmphyllum pertusum, deep
But data are actually Porites asteroides from shallow

Row 5025
SRR9176529, SAMN11849790
Listed in table as being Desmphyllum pertusum, upper shallow
But data are actually Porites asteroides from shallow

Rows 10542, 10543, 10544, 10550, 10551, 10552, 10553, 10554, 10555
These all list Primnoa resedaeformis samples collected in January, but that is incorrect. Those collection cruises were all either August/September or May.

Supplemental Figure 7: Panels c and d are switched in order (i.e., c should be middle left and d should be middle right). For panels e and f, which pertain to water body, the color coding is based on an alphabetical list. It would make more sense to have the color codes follow geography (e.g., the Gulf of Mexico should be in the same color family as the Caribbean Sea, Northern Atlantic and Southern Atlantic Ocean, like all shades of green or blue, rather than each of these being a totally different color. Similarly, the Northern Pacific and Western Pacific should be within a color family rather than blue and pink).

Methods: I tried to use the numbers offered to sort out what was happening but typically ended up more confused. E.g.,
Lines 437-438: 8,782+1,431+1,305+ 520 = 12,038 (was expecting 12,010 based on line 431).
Lines 456-457: 12,808+1,216+651 = 14,675 (was expecting 16,012 based on line 28).
Again, I think some version of the table I suggested for the Results section could help with this.

Lines 482-484: Excellent idea!

Line 487: Typo Aquarickettsia is missing the second 'a'

Line 508: "…greatest abundance of sequences (please insert number of sequences here), and the broadest…"

Reviewer #2 (Remarks to the Author):

This study represents a meta-analysis of Cnidarian microbiome studies, collecting and analyzing an

impressive number of 16S rRNA reads and cataloging the diversity and structure of microbiomes across different Cnidaria Taxa. Overall it represents an impressive collection of data and unification of the datasets and obviously a lot of work that the authors should be congratulated about. The comprehensive nature of the task also represents one of the issues for this dataset however with the overall take home messages/outcomes of the study not overly novel or groundbreaking. The conclusion from the studies are that the Cnidarian microbiome are highly diverse and that host and environmental factors shape the microbial diversity, richness and structural diversity. These finding are well established and repeatedly published in many other studies, albeit without the comprehensive datasets backing the conclusions. The undertaking is truly impressive and this work should be published with the approach and methodology appearing robust (apart from some minor comments below). The one major issue is that because the dataset is so large many of the comparisons are very broad across taxa and geographic and spatial patterns. Hence the potential novel and interesting patterns are masked because of the overwhelming number of samples included in the analysis many of which are subject to inherent limitations of sampling bias, processing bias and contamination. Overall I think the manuscript is well done, I just question if the take home messages is strong enough for nature communications.

Specific Points:
The approach overcomes one limitation of dataset integration – but does not address some other limitations for Cnidarian microbiome comparisons
o Microhabitat differentiation. Studies often are a mix of mucus, tissue, skeleton or coelentron, but never distinguished these compartments within most broad scale sequencing approaches. The current study does compare tissue, skeleton and mucus compartments, but these are often inherently a mix with difficulty in sampling tissues without carrying over mucus microbes. Most studies also crush/blast coral samples and hence the compartments are a mix of everything. There is some very nice studies recently that highlight the low diversity/complexity of coral tissue microbiomes. This is the really where Cnidarian microbiome research needs to go, moving away from taxonomic assessments to understand the function of the microbiome within the microhabitats they reside. This current manuscript is therefore focused on broad scale taxonomic assessments.
o The manuscript also highlights in the text (lines 69-) it does not account for a lack of standardized of sampling, storage, extraction approaches for DNA and capability to detect contamination with samples (at extraction and sequencing steps). The meta-analysis approach potentially smooths out many artefacts in the currently published data but it also contributes to the broad patterns reported which are mostly established in Scleractinian/Cnidarian studies.
o Many of the diversity comparisons across Cnidaria orders, or sampling locations or depth (i.e. Supp Figures and text) would be highly influenced by the number of samples included in the study. For example are alpha diversity metrics for Scleractinia so high just because the vast majority of studies are on these species. hence the pool of sequences and generated AVS is just much large than other orders. Though this maybe somewhat be negotiated by rarified data in the analyses, the simple fact that more studies, more samples and more effort will generate many more sequences and artificially inflate diversity metrics. Lines 150-onwards highlights that scleractinian and non-scleractiian samples were separated for some of the comparisons of host phylogeny etc – does it account though for the Alpha diversity parameters?

• The Abstract highlights cnidarian and poriferan samples reprocessed; though how many Symbiodinaceae and environmental samples were included? What are the environmental samples? I know the abstract should be short it just seems odd to report but have little information. Also in the body of the manuscript it is not clear what Porifera samples were included and why these 7 projects highlighted/included? There are like corals there are a multitude of Sponge 16S studies so just some justification for why these. Perhaps it is in Supp Table 1 – but the Table is overwhelming which is a compliment to the Authors for all the work (amazing) – but difficult also to extract information from.

• The abstract highlights the shear breath of 6.5 million sequences included in the analysis and this was used to identify the breath of Cnidarian diversity. However all the major analyses comparing structure of the microbiome across the samples was based on only studies that look at the V4 region of the 16S rRNA gene. It is not clear (unless I missed it) how many studies this included across the Cnidaria Orders, sampling types, oceanic sites, depth etc. This should be

represented somewhere. The abstract highlights the complete 6.5 million reads (which is amazing) but perhaps oversells the relevant number of reads/studies that provided the more interesting community patterns.

Minor Points
• Abstract; are the 86 Archaeal and Bacterial phyla only from Cnidarian or also from the other samples (Porifera, Symbiodiniaceae and other environmental samples). This is confusing.
• Abstract Lines 31: this conclusion is quiet generic and would be expected (i.e. host and environment structure the microbiome). What is surprising I guess is that sample type (i.e. does mucus or tissues or skeleton dictate the structure, diversity and composition?).
• Abstract – what is the core microbiome – as not consistent agreed idea of what represents the core microbiome (stated in methods I know but the core microbiome is always contentious unless defined well).
• Line 50: "dwardfs" – perhaps a little too subjective as there are countless bacterial diversity studies; rephrase
• Line 246 and elsewhere – is 'unique' the correct term here as it is 16S sequences that are retrieved. They may not be present since they were not retrieved/characterized in the dataset (though still could be present in other Orders and hence not unique). Minor point
• Line 306-311: Can it be inferred from this data that certain orders are reservoirs for other Cnidarian orders. There is no direct evidence and sediment/water column could be equally as diverse.
• Line 327: No testing of phylosymbiosis patterns was undertaken however – so this is very speculative. Also Line 330 – there is no strong evidence for coevolution presented in these studies – yes phylosymbiosis but not coevolution which has very strict/restricted requirement of evidence.
• Line 335: are these patterns again just due to sampling effort since far fewer studies at deeper depths.
• Line 377: Synechococcus/Prochlorococcus most likely ruminant seawater cyanobacteria that get trapped in coral mucus samples perhaps.
• Line 446 why these samples? Not clear the justification for these samples?
• Line 540 – what is meant by tissue compartment?
• Figure 1: The colors are hard to match – for example on the world map there a range of colors/dots that I assume are sampling location – what do the colored dots represents?
• Is figure 4e informative? It shows similar abundance of sequences for all bacterial genera for location, depth and order? Just no

Reviewer #3 (Remarks to the Author):

McCauley et al. describe a comprehensive meta-analysis of published 16S data originating from cnidarian and poriferan species worldwide. They included over 12.000 samples from worldwide sampling efforts. McCauley re-analyse these data in the same pipeline to be able to draw general conclusions. They found correlations between host phylogeny, sampling depth and ocean body and the associated microbes. In addition, they focus on one bacterial genus, Endozoicomonas, most prevalent across cnidaria.

Overall, the study is very solid in its analysis and conclusions, but I miss some novelties that have not appeared in other studies, including also the functional perspective of the cnidarian microbiome. The authors concentrate only on 16S data describing phylogenetic diversity, but ignore the functional diversity, e.g by analyzing available metagenoms. In reading the manuscript, it was not clear to me what could be gained from this analysis. I strongly recommend that the authors restructure the manuscript, e.g., combine results and discussion and focus on the most ecologically relevant comparisons (see e.g. https://www.nature.com/articles/s41467-022-32684-4).

Specific points:

Introduction:
I strongly encourage authors to cite the most relevant original papers in addition to the review

articles. The amount of cited review articles should be reduced.

Results:

Table2/3. Did you compare also marine vs. freshwater cnidarians. In my opinion the salinity is one important variable that needs to be considered in addition.

Line 117: How many samples were included in this subset of samples? Why did the authors choose the V4 region, what are the results with the other 16S regions? It would be interesting to analyze how the choice of 16S primers affects the results.

Line 121: Sampling location is not included in the Supp Tables 2-3. Not clear, which measure was used to get the 45% and 33% results.

Paragraph line 123-136. This paragraph is a good example of the message of all analyses not becoming clear. What are the key insight here? The authors should focus on fewer but ecologically relevant comparisons. I completely miss the link to host ecology.
Line 139. The terms "complex" and "robust" lineages need to be explained. These terms are not familiar to a wide readership.

Line 152: All β-diversity analyses are based on the Bray-Curtis measure. Only the abundance data are considered here. Authors should also include binary measures (e.g. Jaccard) and phylogenetic measures (e.g. UniFrac) to assess the robustness of their results.

Line 211: Not clear, what the authors want to show by Figure 4e. Figure 4d needs better explanations.

Line 218 ff: "…..with deeper cnidarians hosting a more individualized microbiome, whereas shallow water cnidaria generally hosted a more abundant core microbiome". This is an interesting ecological association and should be presented in a Figure.

Paragraph 276: no quantitative conclusions should be drawn for nonbacterial members, since the V4 primers most likely recognize only a minority of the members of these groups. Here, rather archaea- or symbiodiniaceae-specific primers have to be considered.

Discussion:
The discussion needs to be better structured, e.g., by introducing subheadings that highlight the main findings of the manuscript.

Line 329. Phylosymbiosis signals are not necessarily indicative of coevolution. (see also https://journals.plos.org/plosbiology/article?id=10.1371/journal.pbio.1002311). Please discuss your results more carefully in this regard.

Reviewer #1 (Remarks to the Author):

- The authors have made a herculean effort to examine a large volume of 16S rRNA gene amplicon data available from cnidarians and sponges via a bioinformatic meta-analysis with the goal of uncovering trends to better understand the microbial symbioses occurring within these holobionts. These data were derived from relevant studies between 2011–2021 and the metadata curated manually from the publications (since in general, metadata are poorly or inconsistently included with raw DNA sequence submissions to databases). Amassing useful metadata as has been done here is a HUGE amount of work and is why these types of reviews are rare.

Thank you very much for the kind words. We hope that the wider community will utilize this sequence database, as well as the accompanying metadata to address many more relevant questions.

- Lines 69-75 correctly brings up the issue of how different protocols (e.g., preservation, extraction, amplification primers, sequencing platform, bioinformatic pipeline, combinations thereof) complicate or confound cross-study comparisons. Of the multiple versions of datasets examined in this manuscript, the largest (which I'll call #1 = 'maximum cnidarians') includes not only different sequencing platforms (e.g., Roche 454, Illumina MiSeq, HiSeq,), but different primers/variable region targets). However, the bulk of the analyses use slightly smaller datasets (V4, V4_p, subsets thereof) which are restricted to one platform (Illumina MiSeq) and one variable region (V4), limiting two of the major factors (a wise and necessary choice). It should be made a little more explicit in the introduction that this happened and why (e.g., totally valid choice to avoid the difficult-to-quantity amounts of methodological variability introduced by different platforms and variable region
primers in order to increase the quality of the results; Tremblay et al. 2015, DOI 10.3389/fmicb.2015.00771).

We have added new text to highlight and detail this choice in both the abstract, introduction, and methods. (Lines 30-31; 97-99, 118-126).

- The Abstract (in order to meet the small word limit) and the Introduction (lines 86-89) only clue the reader into a single massive dataset being analyzed, but in fact the paper analyzes several and the majority of the analyses are NOT conducted on the single massive dataset (for obvious reasons just mentioned above). In addition to mentioning it in the Introduction (and to expedite matching up the specific dataset from which a given result is derived), I highly recommend that the Results section begin with an overview that makes clear what each of the datasets encompasses (it might make sense to create a table listing the relevant numbers). I have uploaded an example table based on the numbers I found in the manuscript to illustrate what I mean since it is not possible to include it here; in it I attempt to number the databases for clarity and use those numbers in the rest of this review.

Thanks for the suggestion, we have created and added a new table to help direct the readers to the different datasets utilised (Table 1) and now refer to it throughout the manuscript. Additionally, we have created a new section at the start of the results review the main datasets before further discussion of them (Lines 116-126).

- It would also be helpful to clarify the overlaps between these datasets. As written, I assume that V4_P equals V4 (2b) plus sponge and Symbiodiniaceae culture microbiomes but maybe that is not exactly right? The numbers in a table like the mock up I've provided would help clarify those kinds of things too.

Is #4 core microbiome derived from 2b (V4 unrarefied)? It should be noted that the Supplemental Materials DO explicitly describe which underlying dataset was used for each of them (thank you!). I appreciate the choice to group the Results topically, rather than by dataset, but without being able to easily indicate when jumping from one dataset to the next, it makes it hard for the reader to keep track. It might be helpful in the Methods section to describe the flow from the largest dataset down to the various sub-datasets.

The new table (Table 1) should help guide the reader to the various datasets used. The results section now begins with a paragraph designed to inform the reader of the various datasets, including potential overlaps between them (Lines 156-158). Additionally, the methods section now follows the flow of largest to smallest datasets (Line 116-356), which mirrors Table 1. The #4 dataset in your table (core microbiome) is actually derived from all of the studies, and is placed at the end of the methods, as it required an entire re-analysis of the original data.

- The current first section of Results (lines 100-114) reads as if those conclusions are derived from dataset #1 (max cnidarians) but then references Figures 2 and 3 which both say they come from dataset #2 (V4). I suspect that both datasets #1 and #2 support these findings but because of the confounding factors involved in #1 (different variable regions, different sequencing platforms, etc.) it was cleaner to create figures using #2. For transparency's sake, it would be helpful to just explain that (or whatever drove those choices if my guess is wrong).

This is correct, they were found for both but visualized with the second dataset. New text has been added to reflect this (Lines 135; 138-139).

- The second section of Results (lines 116-143) says it comes from #2 V4 and mentions unrarefied and rarefied datasets, so 2a and 2b). The third section of Results (lines 145-175) begins with V4 but mentions separating into scleractinian vs not datasets (which I've named 2.1 and 2.2 in my table). Then lines 153-157 come back to dataset V4 2a/2b. Then we switch to V4_p (line 158), but at the end we come back to 2.1 V4_scleractinian
subset.

First, hopefully Table 1 now allows for an easier understanding of the datasets used. Second, the order of the text has been altered to help the flow of these sections. Specifically, instead of jumping from scleractinian samples up to sponge samples and then back to cnidarians, we have combined the cnidarian samples and then discuss Porifera (Lines 195-205).

- Having now made it through the full Results section, I'm now not sure if some of this might not actually involve subsets of #5?

Technically there is no dataset #5, but it is equivalent to the original 2a and 2b in your table (Table 1). I have added extra text to the results so that the readers understand these are the same datasets (Line 120-124).

- The fourth section of Results (lines 177-212), we start with #1 max cnidarians (line 178) and it appears that the rest of this paragraph and all parts of Figure 4 are based on #1, but then lines 186-188 referencing Fig 4b mentions "ASVs identified in cultured Symbiodiniaceae microbiomes were identified in any cnidarian microbiome" which suggests we have jumped to V4_p.

Symbiodiniaceae microbiome samples were included in both the V3 and V4 libraries. Therefore, this sentence is based off the examination of specific Symbiodiniaceae samples, and not any single dataset. New text has been added to highlight this (Line 219).

- The paragraph beginning with line 203 talks about the scleractinian microbiome so it is unclear if this is based on #1 or 2.1 V4_scleractinian subset.

This is combining data from cnidarian samples across all ASV libraries, new text has been added to reflect this (Line 208-211).

- Based on the title, one would guess that the fifth section of Results (lines 214-237) and Figure 5 is based on #4 (core microbiome) but that is not specified?

New text has been added to reflect this dataset (Lines 250-252).

- Sixth section of Results, line 242 "examined Endozoicomonas ASVs within all libraries; please specify (e.g., 1, 2 and 3? 1-4? 1-5?).

New text has been in this paragraph to detail where these come from, in addition to Table 1 (Lines 305-306).

- Line 261 specifies V4 (2a or 2b?).

This would be from the equivalent of the #2 in your table, neither 2a nor 2b. Hopefully the addition of Table 1 makes this easier to follow now.

- Seventh section of Results (lines 276-290) begins in #1 (based on ASV numbers). Are the 11,995 unidentifiable ASVs also from #1 or from 2b?

The first is correct, these are from all cnidarian samples, not 2b. Additional text has been added to address this (Line 341).

The results support the major conclusions offered:
• That Cnidarians host an incredible diversity of archaeal and bacterial phyla.
• That host phylogeny and ocean body have the largest influence on microbial diversity
• The diversity and geographic/host-specific associations uncovered for Endozoicomonas
I was also particularly struck by some of the minor discoveries; e.g., that Ostreobium was found not only in scleractinians (where I'm used to seeing it, as colored bands growing in the skeleton) but also in Octocorals and black corals.

There are a lot of 'smaller' but potentially ecologically important results that are too numerous for this manuscript. Hopefully utilization of this database by the community at large will realize these. With publication, the dataset will be made available publicly at Figshare.

- Within the conclusion about huge diversity and how the majority of ASVs are incredibly rare <1% prevalence), I think the authors have missed (or chosen to avoid) an opportunity that having a large dataset like this could afford them to answer a vexin g question within the coral/cnidarian community. Most 16S rRNA amplicon surveys conducted prior to 2018 (and some after that), did not include kit contamination blanks. This makes it difficult or impossible to be certain which of those rare ASVs are genuine associates and which are contamination from the extraction kit or other laboratory reagents. This was initially brought up by Salter et al. (2014) DOI 10.1186/s12915-014-0087-z and expanded by Glassing et al. (2016) DOI 10.1186/s13099-016-0103-7 and de Goffau et al. (2018) DOI 10.1038/s41564-018-0202-y because it can have such a large impact on interpretation of low biomass samples (which can include cnidarian samples). The authors mention this in passing in lines 379-380. I would argue that the V4 dataset likely includes enough studies that are on both sides of this shift in community quality requirements (the inclusion of kit blank controls) that it may be possible to determine (especially with ASVs over OTUs) whether core microbiota such as Pseudoalteromonas, Alteromonas, and Propionibacterium are real or contaminants (or both but discernable by ASV sequence). This might even be as simple as going into the core microbiome dataset (mercifully smaller than the full V4) and flagging which studies included kit blank controls and which did not. If these bacterial groups (or other genera flagged by Salter and Glassing as common contaminants) only show up in studies that did not include controls, that is strongly suggestive that they are only contaminants. If they show up in studies that DID run controls and spot checks reveal the controls did not contain them, then that would data rescue this information even for older studies that lacked controls. Which would be another major conclusion to offer.

This is a great idea. We have gone back and retested all studies utilized as part of the relative abundance database that included these controls. We found that the removal of these common contaminants, did not prevent the detection of our key core microbiota. New text has been added throughout the manuscript to address this new finding (Lines 107-110; 290-294; 463-465; 660-661).

- In some cases, sampling depth was found to be a significant factor. I noticed in skimming through Supplemental Table 1 that there are a number of studies for which that information was unknown. It would be helpful to know what percentage of the total studies in whichever dataset(s) found depth to be significant contained this information. E.g., depth information was available for 90/80/70% of the total studies in dataset V4. Large numbers are comforting, while an unknown but non-100% number makes it harder to be convinced by the significance (I want to believe!).

Percentages have been provided to support each of these conclusions (Lines 168-170).

- While this study of cnidarian microbiomes has been under review, a systematic revision was published that elevated anthozoan sub-classes Octocorallia and Hexacorallia to the rank of Class, replaced the 3 Octocorallia orders with 2 new ones, and revised most of the family-level structure. I would not expect this to impact any of your major conclusions but you will want to at least acknowledge this somewhere in the paper, much as you have acknowledged the recent (incredibly annoying) microbial taxonomic changes (e.g., Proteobacteria to Pseudomonadota): McFadden et al. 2022, DOI 10.18061/bssb.v1i3.8735.

Text changes have been made to address these new systematic revisions (Line 69-70, 153-154).

- The manuscript is appropriately referenced, showing a command of the relevant literature. Numbered in-

text citations match the bibliography list and correctly support the statements or methods mentioned. There are a couple of parentheticals (author, date) that should be changed to numbered for consistency:
Line 140: Ying et al. 2018
Line 471: Rstudio reference

These citations have been corrected (Lines 171; 570).

- Figure 1: The legend explains what the pie charts mean and the inset explains the size of the chart and the order each color in the charts represents. Additional text is required to explain what the small colored circles are and what the color-coding means. Presumably each small circle is a study (since there are not 12,010 circles, so they can't be samples) and the colors appear to align with ocean basins?

New text has been added to address both concerns (Line 1077-1081). Additionally, ocean body names have been changed within the figure to match the colored circles.

- A rapid skim uncovered incorrect data in Supplemental Table 1, suggesting that you need to do another round of QC, since the host and environmental data you have compiled is one of the key community benefits of this effort:
Row 4879
SRR9176498, SAMN11849804
Listed in table as being Desmphyllum pertusum, deep
But data are actually Porites asteroides from shallow

Row 5025
SRR9176529, SAMN11849790
Listed in table as being Desmphyllum pertusum, upper shallow
But data are actually Porites asteroides from shallow
Rows 10542, 10543, 10544, 10550, 10551, 10552, 10553, 10554, 10555
These all list Primnoa resedaeformis samples collected in January, but that is incorrect. Those collection cruises were all either August/September or May.

Thank you, an additional QC pass has been made on the metadata.

- Supplemental Figure 7: Panels c and d are switched in order (i.e., c should be middle left and d should be middle right). For panels e and f, which pertain to water body, the color coding is based on an alphabetical list. It would make more sense to have the color codes follow geography (e.g., the Gulf of Mexico should be in the same color family as the Caribbean Sea, Northern Atlantic and Southern Atlantic Ocean, like all shades of green or blue, rather than each of these being a totally different color. Similarly, the Northern Pacific and Western Pacific should be within a color family rather than blue and pink).

The incorrect panel labels have been fixed and panels e/f are now color coded geographically (Supp. Fig. 8). Additionally, this geographical coloring has been utilized in the newly created figures (Supp. Figs. 9-10).

- Methods: I tried to use the numbers offered to sort out what was happening but typically ended up more confused. E.g.,
Lines 437-438: 8,782+1,431+1,305+ 520 = 12,038 (was expecting 12,010 based on line 431).

Lines 456-457: 12,808+1,216+651 = 14,675 (was expecting 16,012 based on line 28).
Again, I think some version of the table I suggested for the Results section could help with this.

I have edited the text alongside creating Table 1 to clarify these numbers (Lines 532-534; 554-557).

- Lines 482-484: Excellent idea!

Thanks, this also serves as an additional asset that will be provided and can be used by the community for future analyses.

- Line 487: Typo Aquarickettsia is missing the second 'a'

Thank you very much for finding this spelling mistake. It has been corrected (Line 585).

- Line 508: "…greatest abundance of sequences (please insert number of sequences here), and the broadest…"

The number of sequences has been added (Line 596-598) and is provided by Table 1.

Reviewer #2 (Remarks to the Author):

-This study represents a meta-analysis of Cnidarian microbiome studies, collecting and analyzing an impressive number of 16S rRNA reads and cataloging the diversity and structure of microbiomes across different Cnidaria Taxa. Overall it represents an impressive collection of data and unification of the datasets and obviously a lot of work that the authors should be congratulated about. The comprehensive nature of the task also represents one of the issues for this dataset however with the overall take home messages/outcomes of the study not overly novel or groundbreaking. The conclusion from the studies are that the Cnidarian microbiome are highly diverse and that host and environmental factors shape the microbial diversity, richness and structural diversity. These finding are well established and repeatedly published in many other studies, albeit without the comprehensive datasets backing the conclusions. The undertaking is truly impressive and this work should be published with the approach and methodology appearing robust (apart from some minor comments below). The one major issue is that because the dataset is so large many of the comparisons are very broad across taxa and geographic and spatial patterns. Hence the potential novel and interesting patterns are masked because of the overwhelming number of samples included in the analysis many of which are subject to inherent limitations of sampling bias, processing bias and contamination. Overall I think the manuscript is well done, I just question if the take home messages is strong enough for nature communications.

Thank you very much for the feedback. We appreciate the praise for this first Cnidarian microbiome meta-analysis which is shared by the other reviewers. In our revision we have added new text and reformatted previous text to highlight all of our major findings, not just the host and geographic structuring. Indeed, while previous studies have identified host and geographically structuring, primarily in scleractinian corals, our much larger unified database identifies this for the first time across Cnidaria, including traditionally understudied non-scleractinian species, allowing for a more robust testing of these hypotheses. Unlike many of these previous studies we were able to leverage this database to calculate the relative strength of these factors against many other ecologically and methodological parameters, including depth, hosting Symbiodiniaceae, microhabitat, lifestage, collection time, waterbody, extraction kit etc.

We would like to highlight here additional aspects of our analysis which we believe is of interest and valuable for the scientific community:

Major novel findings already present;

- -An extreme microbial diversity that rivals that found across Earth only a few years ago.
- -Identifying the relative abundances of all cnidarian microbiota, i.e.. transitory v ‘core’.
- -Identifying the primary ecological factors influencing cnidarian core microbiota.
- -Revealing differences in microbial complexity across cnidarian orders.
- -Highlighting potential ecologically important cnidarian microbes.
- -Illustrating the host/geographical presence and fidelity of *Endozoicomonas.*

Smaller novel findings already present;

- -Differences in microbiome between Complex/Robust coral clades.
- -More widespread presence of ecologically important microbiota ie. Corallicolids, *Ostreobium.*
- -Soft corals sharing as many microbiota with other Cnidaria as hard corals.
- -Lack of microbes shared between Symbiodiniaceae and host Cnidarians.

New major findings added with revisions (added to abstract or introduction, and discussion);

- -Testing and relevance of common kit contaminants (Lines 107-110; 290-294; 463-465; 660-661).

-Difference in relative abundances, and common shared microbiota across microhabitats (Lines 33; 105-106; 161-163; 243-247; 260-270; 280-285; 297-300; 451-452; 637) and (Supp. Figs. 14-16).

-Inherent variability/bias of clustering v unique sequence variants across cnidaria and microhabitats (Lines 105-107; 260-270; 474-476; 503-505) and (Supp. Figs. 14-16).

Additionally, all of the data that this manuscript is based on, sequencing database, metadata, and sequencing taxonomic files, will also be released as community tools (Figshare project linked; Line 676-678), which allows anybody to utilize the previous 10 years of cnidarian microbiome data for any question that they're interested in.

- Specific Points:
The approach overcomes one limitation of dataset integration – but does not address some other limitations for Cnidarian microbiome comparisons
o Microhabitat differentiation. Studies often are a mix of mucus, tissue, skeleton or coelentron, but never distinguished these compartments within most broad scale sequencing approaches. The current study does compare tissue, skeleton and mucus compartments, but these are often inherently a mix with difficulty in sampling tissues without carrying over mucus microbes. Most studies also crush/blast coral samples and hence the compartments are a mix of everything.

We agree that this is a problem that is currently present across cnidarian studies. Unfortunately, as this is a review, we have little control of this. We did examine each manuscript closely when collecting the original metadata to identify which microhabitat was extracted (Supp. Table 1). New text has been added to specifically address the concern of mixed compartments, and the need to differentiate microhabitats more carefully in future functional studies (Lines 243-247; 260-270; 503-505).

In response to this comment however, we did further examine differences between the microbial communities present within studies that carefully extracted from mucus, and skeletal samples (Lines 33; 105-106; 161-163; 243-247; 260-270; 280-285; 297-300; 451-452; 637) and (Supp. Figs. 14-16).

- There is some very nice studies recently that highlight the low diversity/complexity of coral tissue microbiomes. This is the really where Cnidarian microbiome research needs to go, moving away from taxonomic assessments to understand the function of the microbiome within the microhabitats they reside. This current manuscript is therefore focused on broad scale taxonomic assessments.

We completely agree with this statement. The concept behind this database/manuscript was to review and synthesize the majority of cnidarian microbiome research to date, which has primarily focused on taxonomic identification (16S rRNA). Our goal is to utilize this information with the target of highlighting important microbes for future functional analyses. This is why specific attention was given towards identifying bacteria that are not only highly abundant and prevalent, but also those that are shared across taxa, in order for future studies to identify their potentially beneficial function within Cnidaria.

Additionally, this is only the first rendition of this database, which took nearly 3 years to build and analyze. The next step will be the inclusion of whole genome shotgun sequencing data, and eventually proteomic and transcriptomic sequences. Each of these will take some time to complete and will be integrated into the freely available community database.

- The manuscript also highlights in the text (lines 69-) it does not account for a lack of standardized of sampling, storage, extraction approaches for DNA and capability to detect contamination with samples (at

extraction and sequencing steps). The meta-analysis approach potentially smooths out many artefacts in the currently published data but it also contributes to the broad patterns reported which are mostly established in Scleractinian/Cnidarian studies.

This is true, the steps taken to reduce data complexity allow for broad questions. While some of these findings have been previously identified in scleractinians, many have not yet been examined in other cnidarians, or are completely novel (see list above).

- Many of the diversity comparisons across Cnidaria orders, or sampling locations or depth (i.e. Supp Figures and text) would be highly influenced by the number of samples included in the study. For example are alpha diversity metrics for Scleractinia so high just because the vast majority of studies are on these species. Hence the pool of sequences and generated AVS is just much large than other orders. Though this maybe somewhat be negotiated by rarified data in the analyses, the simple fact that more studies, more samples and more effort will generate many more sequences and artificially inflate diversity metrics. Lines 150-onwards highlights that scleractinian and non-scleractiian samples were separated for some of the comparisons of host phylogeny etc – does it account though for the Alpha diversity parameters?

The sampling bias towards scleractinian corals is definitely present, and we acknowledge it multiple times in the manuscript. To address this, we have taken multiple steps, including separating the scleractinian and non-scleractinian datasets, in addition to rarefying the data, which does help to address the bias (but not completely remove) for both alpha and beta diversities.

To further address this question, we have now conducted additional analyses into the V4_healthy_cnidarian_unrarefied dataset to examine the most obvious difference amongst alpha diversity, ASV richness. Using a well-established tool for standardizing and extrapolating communities from different samples, we have identified that even with the sampling bias towards scleractinian samples, the vast majority (~90%) have a richness similar to under sampled cnidarians (Supp. Fig 3). A great example can be found with Actiniaria and Antipatharia, whereby Actiniaria has more than double the number of samples than Antipatharia. With this hypothesis we would therefore expect an increase in actiniarian sequences and an artificially inflated ASV richness metric. However, not only is this not the case, as they both host approximately the same number of ASVs, but per sample Actiniaria actually host approximately half the richness of Antipatharia (Fig 3a; Supp. Fig. 3). This pattern is present across many of the cnidarian orders, which are also under sampled compared to each other.

- The Abstract highlights cnidarian and poriferan samples reprocessed; though how many Symbiodinaceae and environmental samples were included? What are the environmental samples? I know the abstract should be short it just seems odd to report but have little information. Also in the body of the manuscript it is not clear what Porifera samples were included and why these 7 projects highlighted/included? There are like corals there are a multitude of Sponge 16S studies so just some justification for why these. Perhaps it is in Supp Table 1 – but the Table is overwhelming which is a compliment to the Authors for all the work (amazing) – but difficult also to extract information from.

With an increased word count in the abstract, we have been able to increase the amount of detail. Additional information has been added to the abstract and results to address this question (Lines 28-29; 116-118). Additional text has been added to specifically identify the inclusion of a previous global assessment of Porifera microbiota, which allowed for the comparison of over 200 different sponge species from many different depth and geographical locations (Lines 541-546).

- The abstract highlights the shear breath of 6.5 million sequences included in the analysis and this was used to identify the breath of Cnidarian diversity. However all the major analyses comparing structure of the microbiome across the samples was based on only studies that look at the V4 region of the 16S rRNA gene. It is not clear (unless I missed it) how many studies this included across the Cnidaria Orders, sampling types, oceanic sites, depth etc. This should be represented somewhere. The abstract highlights the complete 6.5 million reads (which is amazing) but perhaps oversells the relevant number of reads/studies that provided the more interesting community patterns.

This is a great point and to address this we have not only added text throughout the manuscript (Lines 30-31; 34; 95-99; 116-126; 146; 180; 193; 208-211; 250-252; 290-291; 305-306; 341; 597), but also created Table 1 to specify the exact number of studies, samples, species, and sequences that are present in each dataset.

Minor Points

• Abstract; are the 86 Archaeal and Bacterial phyla only from Cnidarian or also from the other samples (Porifera, Symbiodiniaceae and other environmental samples). This is confusing.

Additional text has been added to specify cnidarian microbiota (Line 32).

- Abstract Lines 31: this conclusion is quiet generic and would be expected (i.e. host and environment structure the microbiome). What is surprising I guess is that sample type (i.e. does mucus or tissues or skeleton dictate the structure, diversity and composition?).

While expected, host and geographic influence had not been previously tested on numerous non-scleractinian Cnidaria. However, with additional text allowances by the editor we have expanded this conclusion to reveal the different influences between host and geography, vs. microhabitat and month of sampling (Lines 36-37). Microhabitat is significantly correlated to alpha and beta diversity indices across scleractinia, but to a lesser degree than host and water body. However, in non-scleractinian samples while it significantly influenced beta diversity metrics, it was not correlated to alpha diversity metrics (Supp. Tables 2-3).

- Abstract – what is the core microbiome – as not consistent agreed idea of what represents the core microbiome (stated in methods I know but the core microbiome is always contentious unless defined well).

The term ‘core microbiota’ has been removed from the abstract but defined in the first mention of the introduction (Lines 102-105).

- Line 50: “dwardfs” – perhaps a little too subjective as there are countless bacterial diversity studies; rephrase

This has been replaced with a more suitable word (Line 53).

- Line 246 and elsewhere – is ‘unique’ the correct term here as it is 16S sequences that are retrieved. They

may not be present since they were not retrieved/characterized in the dataset (though still could be present in other Orders and hence not unique). Minor point

When referring to the number of ASVs present the word ‘unique’ has been removed (Lines 208-211; 214-217; 238-239). In the instance where it is used to represent sequence parameters it has been maintained (Lines 107; 260-201-265-268; 503-505).

- Line 306-311: Can it be inferred from this data that certain orders are reservoirs for other Cnidarian orders. There is no direct evidence and sediment/water column could be equally as diverse.

We have edited our text to now only posit the potential ability to serve as reservoirs (Line 374-381). The shared cnidarian microbiota were not identified in the water column samples in this database, nor cultured Symbiodiniaceae, or poriferan samples. It is definitely possible/likely that there are other sources, but that does not prevent these orders from being a possible source.

- Line 327: No testing of phylosymbiosis patterns was undertaken however – so this is very speculative. Also Line 330 – there is no strong evidence for coevolution presented in these studies – yes phylosymbiosis but not coevolution which has very strict/restricted requirement of evidence.

We agree and have removed the discussion of coevolution from the paper. We have edited the text to clearly state hypotheses that we did not test for (Line 401-404).

- Line 335: are these patterns again just due to sampling effort since far fewer studies at deeper depths.

We respectfully disagree and revisited the data to identify. Indeed, this was a consistently strong pattern that was found across multiple depths, irrespective of sampling density and taxa (Supp. Figs. 4-6).

- Line 377: Synechococcus/Prochlorococcus most likely ruminant seawater cyanobacteria that get trapped in coral mucus samples perhaps.

We totally agree with this, and this was initially the intent of including the text “often found in seawater columns”, however additional text has been added to make this very clear (Line 459-461).

- Line 446 why these samples? Not clear the justification for these samples?

Additional text has been added to justify the inclusion of these additional samples (Lines 541-546).

- Line 540 – what is meant by tissue compartment?

This text has been replaced with the more appropriate term “microhabitat” (Line 630).

- Figure 1: The colors are hard to match – for example on the world map there a range of colors/dots that I assume are sampling location – what do the colored dots represents?

New text has been added to address this (Line 1077-1081). Additionally, ocean body names have been changed within the figure to match the colored circles.

- Is figure 4e informative? It shows similar abundance of sequences for all bacterial genera for location, depth and order? Just no

Fig. 4e has a lot of information in it and the text has been edited to reflect this (Line 1095-1102). First it identifies the ASVs that were identified in either a) more than one ocean body, 2) across depth, 3) across Order. Additionally, there is significant overlap in the bacterial genera for these three comparisons. Therefore, 4e reveals a subset of bacterial genera that should be considered highly shared in cnidarians found across the world’s oceans, across depth and across taxa.

Reviewer #3 (Remarks to the Author):

- McCauley et al. describe a comprehensive meta-analysis of published 16S data originating from cnidarian and poriferan species worldwide. They included over 12.000 samples from worldwide sampling efforts. McCauley re-analyse these data in the same pipeline to be able to draw general conclusions. They found correlations between host phylogeny, sampling depth and ocean body and the associated microbes. In addition, they focus on one bacterial genus, Endozoicomonas, most prevalent across cnidaria.

Overall, the study is very solid in its analysis and conclusions, but I miss some novelties that have not appeared in other studies, including also the functional perspective of the cnidarian microbiome.

Thank you very much for the feedback. We appreciate the praise for this first Cnidarian microbiome meta-analysis which is shared by the other reviewers. In our revision, we have added new text and reformatted previous text to highlight all of our major findings, not just the host and geographic structuring. Indeed, while previous studies have identified host and geographically structuring, primarily in scleractinian corals, our much larger unified database identifies this for the first time across Cnidaria, including traditionally understudied non-scleractinian species, allowing for a more robust testing of these hypotheses. Unlike many of these previous studies we were able to leverage this database to calculate the relative strength of these factors against many other ecologically and methodological parameters, including depth, hosting Symbiodiniaceae, microhabitat, lifestage, collection time, waterbody, extraction kit etc.

We would like to highlight here additional aspects of our analysis which we believe is of interest and valuable for the scientific community.

Major novel findings already present;

- -An extreme microbial diversity that rivals that found across Earth only a few years ago.
- -Identifying the relative abundances of all cnidarian microbiota, i.e.. transitory v ‘core’.
- -Identifying the primary ecological factors influencing cnidarian core microbiota.
- -Revealing differences in microbial complexity across cnidarian orders.
- -Highlighting potential ecologically important cnidarian microbes.
- -Illustrating the host/geographical presence and fidelity of *Endozoicomonas.*

Smaller novel findings already present;

- -Differences in microbiome between Complex/Robust coral clades.
- -More widespread presence of ecologically important microbiota ie. Corallicolids, *Ostreobium*.
- -Soft corals sharing as many microbiota with other Cnidaria as hard corals.
- -Lack of microbes shared between Symbiodiniaceae and host Cnidarians.

New major findings added with revisions (added to abstract or introduction, and discussion);

- -Testing and relevance of common kit contaminants (Lines 107-110; 290-294; 463-465; 660-661).
- -Difference in relative abundances, and common shared microbiota across microhabitats (Lines 33; 105-106; 161-163; 243-247; 260-270; 280-285; 297-300; 451-452; 637) and (Supp. Figs. 14-16).
- -Inherent variability/bias of clustering v unique sequence variants across cnidaria and microhabitats (Lines 105-107; 260-270; 474-476; 503-505) and (Supp. Figs. 14-16).

Additionally, all of the data that this manuscript is based on, sequencing database, metadata, and sequencing taxonomic files, will also be released as community tools (Figshare project linked; Line 676-678), which allows anybody to utilize the previous 10 years of cnidarian microbiome data for any question that they’re interested in.

- The authors concentrate only on 16S data describing phylogenetic diversity, but ignore the functional diversity, e.g by analyzing available metagenoms.

The concept behind this database/manuscript was to review and synthesize the majority of cnidarian microbiome research to date, which has primarily focused on taxonomic identification (16S rRNA). Our goal is to utilize this information with the target of highlighting important microbes for future functional analyses. This is why specific attention was given towards identifying bacteria that are not only highly abundant and prevalent, but also those that are shared across taxa, in order for future studies to identify their potentially beneficial function within Cnidaria.

Additionally, this is only the first rendition of this database, which took nearly 3 years to build and analyze. The next step will be the inclusion of whole genome shotgun sequencing data, and eventually proteomic and transcriptomic sequences. Each of these will take some time to complete and will be integrated into the freely available community database.

- In reading the manuscript, it was not clear to me what could be gained from this analysis. I strongly recommend that the authors restructure the manuscript, e.g., combine results and discussion and focus on the most ecologically relevant comparisons (see e.g. https://www.nature.com/articles/s41467-022-32684-4).

This meta-analysis unifies previously published cnidarian microbiome data sets and will be made available to the community to be mined for specific research questions. In our chosen case study, we focused on the diversity and geographic/host-specific associations of *Endozoicomonas* which we believe is of increasing interest to the coral community.

We respectfully disagree with the suggestion to restructure the manuscript. We think of this submission as a review or database and detail specifics of the utilized massive data sets in *Results*, while we use the *Discussion* section for specific applications of the created database.

Specific points:

- Introduction:
I strongly encourage authors to cite the most relevant original papers in addition to the review articles. The amount of cited review articles should be reduced.

Thank you for your feedback. 14 review articles have been removed from the introduction and substituted with 13 relevant original papers, dating back to 1881 (Lines 50-56; 65-66). The reviews have been maintained only in one sentence, which is used to reinforce the argument that there are a large number of review articles in the cnidarian microbiome field (Line 60-64).

Results:

- Table2/3. Did you compare also marine vs. freshwater cnidarians. In my opinion the salinity is one important variable that needs to be considered in addition.

We agree with the statement, however we are bound to the published studies. There were only 14 freshwater samples present in the V4 region, whereas there were 4,229 marine samples. For that reason, marine vs. freshwater was not tested given the bias of sampling. Furthermore, the freshwater samples are split across cnidarian classes, which would further hinder appropriate analysis.

- Line 117: How many samples were included in this subset of samples? Why did the authors choose the V4 region, what are the results with the other 16S regions? It would be interesting to analyze how the choice of 16S primers affects the results.

A new table (Table 1) has been added that details the number of studies, species, samples and sequences for each of the data subsets. The V4 subset was chosen because it provided the widest variability of samples from various cnidarian taxa (11 cnidarian orders) (Lines 596-598). Given the frequency of the amplification of the V4 region in microbial studies, it is commonly chosen as the region used to build microbial catalogues (Thompson et al., 2017).
Given the significant drop off of non-scleractinian samples in regions other than V4, and therefore the increase in scleractinian sampling bias, we decided not to test the additional subsets. We identify in our conclusions that future research into the choice of primer, amongst other methodological choices, is important (Lines 506-507).

- Line 121: Sampling location is not included in the Supp Tables 2-3. Not clear, which measure was used to get the 45% and 33% results.

Sampling location is referred to as Water Body in Supp tables 2-3, text has been added to avoid this confusion (Line 150). These values were taken from ASVs Observed (scleractinian), and Shannon (non-scleractinian).

- Paragraph line 123-136. This paragraph is a good example of the message of all analyses not becoming clear. What are the key insight here? The authors should focus on fewer but ecologically relevant comparisons. I completely miss the link to host ecology.

We appreciate the feedback. Our goal was to stress the generality of the data set and its universal applicability; therefore, we discuss numerous ecological comparisons and differences. With the data base being made freely available, we hope to excite others to mine the data set for their specific interests and questions.

- Line 139. The terms "complex" and "robust" lineages need to be explained. These terms are not familiar to a wide readership.

Additional text has been added to give definitions, although even within the field these are considered somewhat nebulous. (Line 170-173).

- Line 152: All β-diversity analyses are based on the Bray-Curtis measure. Only the abundance data are considered here. Authors should also include binary measures (e.g. Jaccard) and phylogenetic measures (e.g. UniFrac) to assess the robustness of their results.

This is correct. Given the size of the datasets, the computing power, and the time required to process these analyses, additional beta diversity metrics (aside from Bray-Curtis) were not included in the first draft. In response to this request, we have now processed the six different V4_healthy_MiSeq datasets using the Jaccard Index and we are delighted that these additional analyses support the initial findings. Additional text has been added (Lines 239; 244; 797-801), and (Supp. Figs. 9-10), and (Supp. Tables 2-3; 6-9).

Unfortunately, we were not able to add the W-Unifrac phylogenetic metric, due to limitations in computing and manpower (the first and responding author is now on parental leave). We spent the last three months trying to analyze the V4_healthy_MiSeq data sets using a Unifrac metric, but we constantly encountered computational limits, even with the assistance of a coding/computational expert. The sheer size of this these four large datasets is not amendable to these commonly used, but not well scale-able R-packages. Our computational experts estimated that we would require a computer server with a minimum of 620GB of RAM to process just one of these datasets and that it would take almost a month to run. The only way to feasibly conduct these computations would be to rent a high functioning server on UF's HyperGator facility (one of the fastest super computers in the world) for which we would need an account and the service would cost ~$15,500 and take several months to complete.

The global microbiome database specifically identified computational scaling issues with the calculation of Unifrac using large collections of sequences (Thompson et al., 2017). While they used a python-based package to create a workaround, this is currently not compatible with the phyloseq data structure utilised in our database. Additionally, other recent 16S rRNA analyses published within the Nature Group similarly do not include Unifrac analyses (Bowerman et al., 2020; Kurilshikov et al., 2021).

While we agree that phylogenic measure would add to the study, given these monetary and time constraints, we have decided not to include Unifrac analysis. The data will be made freely available, and we hope to inspire other groups with more computational expertise to access the data.

- Line 211: Not clear, what the authors want to show by Figure 4e. Figure 4d needs better explanations.

Additional text has been added to the Figure 4 legend (Line 1095-1102). Additionally, Fig. 4d has been edited for easier understanding. Fig. 4e text has been edited. First it identifies the ASVs that were identified in either a) more than one ocean body, 2) across depth, 3) across Order. Additionally, there is significant overlap in the bacterial genera for these three comparisons. Therefore, Fig. 4e reveals a subset of bacterial genera that should be considered highly shared in cnidarians found across the world's oceans, across depth and across taxa.

- Line 218 ff: "…..with deeper cnidarians hosting a more individualized microbiome, whereas shallow water cnidaria generally hosted a more abundant core microbiome". This is an interesting ecological association and should be presented in a Figure.

Thank you, this is one of the datasets that is represented in Fig. 5. I have added text referring to this figure (Line 257).

- Paragraph 276: no quantitative conclusions should be drawn for nonbacterial members, since the V4 primers most likely recognize only a minority of the members of these groups. Here, rather archaea- or symbiodiniaceae-specific primers have to be considered.

This is a good point and new text has been added to specifically raise this point (Line 474-476).

- Discussion:
The discussion needs to be better structured, e.g., by introducing subheadings that highlight the main findings of the manuscript.

Subheadings have now been added to each paragraph of the discussion (Lines 359-494).

- Line 329. Phylosymbiosis signals are not necessarily indicative of coevolution. (see also https://journals.plos.org/plosbiology/article?id=10.1371/journal.pbio.1002311). Please discuss your results more carefully in this regard.

The discussion of coevolution from these papers has been entirely removed. We have edited the text to clearly indicate our hypotheses, and that we did not test for them (Line 401-404).

Bibliography:

Bowerman, K.L., Rehman, S.F., Vaughan, A., Lachner, N., Budden, K.F., Kim, R.Y., Wood, D.L., Gellatly, S.L., Shukla, S.D., Wood, L.G. and Yang, I.A., 2020. Disease-associated gut microbiome and metabolome changes in patients with chronic obstructive pulmonary disease. *Nature communications*, *11*(1), p.5886.

Thompson, L.R., Sanders, J.G., McDonald, D., Amir, A., Ladau, J., Locey, K.J., Prill, R.J., Tripathi, A., Gibbons, S.M., Ackermann, G. and Navas-Molina, J.A., 2017. A communal catalogue reveals Earth's multiscale microbial diversity. *Nature*, *551*(7681), pp.457-463.

Kurilshikov, A., Medina-Gomez, C., Bacigalupe, R., Radjabzadeh, D., Wang, J., Demirkan, A., Le Roy, C.I., Raygoza Garay, J.A., Finnicum, C.T., Liu, X. and Zhernakova, D.V., 2021. Large-scale association analyses identify host factors influencing human gut microbiome composition. *Nature genetics*, *53*(2), pp.156-165.

REVIEWERS' COMMENTS

Reviewer #1 (Remarks to the Author):

The revised manuscript and figures, particularly due to the inclusion of Table 1 and topical headers in the main sections, is substantially improved and much more readable. The authors clearly made a genuine effort to respond to all review comments and when possible, provided additional analyses. Great job!

Minor copy edit points: Propionibacterium is misspelled in several places (e.g., lines 293, 462, 464).

The manuscript (e.g., line 118) says 16,013 samples, but Slide 19 lists Supplemental Table 1 as having metadata for 16,012 microbial samples.

Reviewer #2 (Remarks to the Author):

This revised manuscript has addressed many concerns from this first submission. The study represents an impressive collection of data and unification of the datasets. Overall I think the manuscript is well done, I still question if the take home messages is strong though accept the study is highly valuable for the field and should be published.

Additional minor points are listed below

- Line 33: what microbes are shared and highly abundant in the Alcyonacea and Actinaria? I know abstracts are brief but important relevant information should be provided, otherwise it reads as just too generic.
- Line 93: should this resource (database/data) not already be freely available for publication purposed and open access requirements. Note: methods indicate the data is available – hence edit sentence to convey this point.
- Line 94: sentence grammar needs correction (…for further exploration..)
- Last two paragraphs of introduction should be combined as both detail objectives and approach of this study.
- Line 108: remove "non-contaminant" – it may have been raised by another reviewer and agree contamination is a major ongoing issue for coral 16S studies; but by nature the abundant taxa reported over multiple studies are not the contaminants (especially Endozoicomonas) which is well documented and visualized in coral studies.
- Line 110; Last sentence of Introduction should be removed as there was not functional exploration of the 16S datasets (which really is not possible/appropriate anyway).
- Line 247: Perhaps reword to avoid confusion here. Instead of stating contamination potential (which indicates spurious sequences) perhaps be more specific; i.e. methods that minimize overlap in microorganisms derived from the mucus, tissue and skeleton microhabitats of the coral holobiont" or something similar.
- Line 287 and 293: Just a side note and no absolute evidence to support this; though the Propionibacteria are a common kit contaminant in historic illumina datasets. I am always suspicious when this group pops up; perhaps worth a check if dominant (despite being a core member). No change required – just an observation. Opps though see discussion point made in line 462.
- Line 340 – this section on nonbacterial members could be removed or moved to Supp material; It is slightly problematic as it is not a true representation of these groups due to inherent and strong bias in all the studies which target the bacterial groups. The last sentence (Line 354 highlights this strong limitation anyway). Hence this is a slight distraction of the focus of the paper on the bacterial community patters and the papr would not suffer if this section is inserted into the supplementary material.

Reviewer #3 (Remarks to the Author):

I have no further requests.

Reviewer #1 (Remarks to the Author):

- The revised manuscript and figures, particularly due to the inclusion of Table 1 and topical headers in the main sections, is substantially improved and much more readable. The authors clearly made a genuine effort to respond to all review comments and when possible, provided additional analyses. Great job!

Thank you for your suggested revisions.

- Minor copy edit points: Propionibacterium is misspelled in several places (e.g., lines 293, 462, 464).

Thank you for picking these up. All have been corrected.

The manuscript (e.g., line 118) says 16,013 samples, but Slide 19 lists Supplemental Table 1 as having metadata for 16,012 microbial samples.

Thank you for noticing this error. Line 118 has been correct to 16,012.

Reviewer #2 (Remarks to the Author):

This revised manuscript has addressed many concerns from this first submission. The study represents an impressive collection of data and unification of the datasets. Overall I think the manuscript is well done, I still question if the take home messages is strong though accept the study is highly valuable for the field and should be published.

Thank you for your suggested revisions and for taking time to review the manuscript again.

Additional minor points are listed below

• Line 33: what microbes are shared and highly abundant in the Alcyonacea and Actinaria? I know abstracts are brief but important relevant information should be provided, otherwise it reads as just too generic.

This line has been edited for edification. This line is to emphasize the overall structure of microbiomes across Cnidaria, specifically highlighting the highly abundant core microbiota of Alcyonacea and Actinaria that is distinct from the more 'individual' microbiome of scleractinians and most deeper cnidarians.

• Line 93: should this resource (database/data) not already be freely available for publication

purposed and open access requirements. Note: methods indicate the data is available – hence edit sentence to convey this point.

This sentence has been edited to highlight the now available data.

• Line 94: sentence grammar needs correction (…for further exploration..)

This sentence has been edited.

• Last two paragraphs of introduction should be combined as both detail objectives and approach of this study.

These two paragraphs have been combined.

• Line 108: remove "non-contaminant" – it may have been raised by another reviewer and agree contamination is a major ongoing issue for coral 16S studies; but by nature the abundant taxa reported over multiple studies are not the contaminants (especially Endozoicomonas) which is well documented and visualized in coral studies.

This phrase has been removed.

• Line 110; Last sentence of Introduction should be removed as there was not functional exploration of the 16S datasets (which really is not possible/appropriate anyway).

This line has been removed.

• Line 247: Perhaps reword to avoid confusion here. Instead of stating contamination potential (which indicates spurious sequences) perhaps be more specific; i.e. methods that minimize overlap in microorganisms derived from the mucus, tissue and skeleton microhabitats of the coral holobiont" or something similar.

Fantastic suggestion, I appreciate the edits that avoid confusion with microhabitat contamination, especially after introducing the discussion of kit contaminants.

• Line 287 and 293: Just a side note and no absolute evidence to support this; though the Propionibacteria are a common kit contaminant in historic illumina datasets. I am always suspicious when this group pops up; perhaps worth a check if dominant (despite being a core member). No change required – just an observation. Opps though see discussion point made in line 462.

Yes, this is something I will be following more closely in the future to determine potential kit contaminants.

• Line 340 – this section on nonbacterial members could be removed or moved to Supp material; It is slightly problematic as it is not a true representation of these groups due to inherent and strong bias in all the studies which target the bacterial groups. The last sentence (Line 354 highlights this strong limitation anyway). Hence this is a slight distraction of the focus of the paper on the bacterial community patters and the papr would not suffer if this section is inserted into the supplementary material.

This section has been highly edited and trimmed to remove discussion of Archaea, and any comparison of the non-bacterial community to the bacterial community. We do not wish to use this section to compare microbiota, as you are correct this is not a true representation of non-bacterial members. However, we believe that we can still use available data to highlight two benefits of this database, the first is to examine the effectiveness of using a cnidarian specific training dataset during sequence processing. The second is the ability to examine previously released datasets for possible identification of non-bacterial members i.e., revealing a much more widespread prevalence of Apicomplexan Related Lineages, and *Ostreobium*.

Reviewer #3 (Remarks to the Author):

I have no further requests.

Thank you for taking your time to review the manuscript again.
